# Redefining cell death: ferroptosis as a game-changer in ophthalmology

**DOI:** 10.3389/fimmu.2025.1709354

**Published:** 2025-11-28

**Authors:** Yuan Wei, Yumeng Lin, Youjiaxi Li, Jiaxuan Liu, Yaqi Yang, Haoran Chen, Zhongyu Han, Ke Wang, Tao Qian, Yuan Ju, Wei Zheng

**Affiliations:** 1 College of Chinese Medicine, Changchun University of Chinese Medicine, Changchun, Jilin, China; 2 Department of Nanjing Tongren Eye Center, Nanjing Tongren Hospital, School of Medicine, Southeast University, Nanjing, China; 3 Ophthalmology Department, Affiliated Hospital of Changchun University of Traditional Chinese Medicine, Changchun,Jilin, China; 4 Chengdu Xinhua Hospital, Chengdu, China; 5 School of Medicine, Southeast University, Nanjing, China; 6 Deyang Hospital, Affiliated Hospital of Chengdu University of Traditional Chinese Medicine, Deyang, China; 7 Department of Thyroid and Breast Surgery, Affiliated Hospital of Intergrated Traditional Chinese and Western Medicine, Nanjing University of Chinese Medicine, Nanjing, China

**Keywords:** ferroptosis, eye, therapy, iron metabolism, TAO

## Abstract

Ferroptosis, recently proposed as a novel type of cell death, is characterized by unique characteristics and recognition functions. It is involved in diverse physiological processes and in the onset and progression of various diseases and is characterized by reactions between reactive oxygen species (ROS) and iron-dependent lipid peroxidation. This process is finely regulated by a variety of metabolic pathways. Ferroptosis fundamentally differs from conventional cell death mechanisms such as apoptosis, necrosis, and autophagy. In recent years, research on ferroptosis in the field of ophthalmology has gradually emerged, and a large amount of evidence has shown that it is closely related to the occurrence and development of ophthalmic diseases such as age-related macular degeneration (AMD), diabetic retinopathy (DR), retinal ischemia–reperfusion injury (RIRI), retinitis pigmentosa, dry eye disease, cataracts, and glaucoma. This paper provides a comprehensive review of the latest advancements in ferroptosis within ophthalmological research and systematically describes the molecular mechanisms and pathophysiological significance of ferroptosis in the pathogenesis and progression of ophthalmic diseases. Exploring the mechanisms of ferroptosis holds promise for the delivery of novel molecular targets and therapeutic approaches to prevent and treat ophthalmic diseases. Additionally, its clinical translational and application are anticipated to surmount current therapeutic limitations and emerge as a significant direction for breakthroughs in the precision medicine era.

## 1 Introduction

Unlike most animal eyes, human eyes do more than just measure the intensity of ambient light (1). Clinical studies have shown that visual deprivation directly leads to impaired plasticity in the cerebral cortex, and this neurodevelopmental deficit is irreversible (2). The World Health Organization (WHO) ranks blinding eye disease, cardiovascular disease, and malignant tumors as the three major disabling diseases worldwide. Some surveys have indicated that eye diseases may even increase the risk of developing depression and reduce life expectancy among patients (3). When eye disease occurs, it undoubtedly affects the development of personal living standards and society, and this harm is more obvious in today’s aging society. The pathology of ocular diseases involves multiple cell death mechanisms, including apoptosis, necrosis, and autophagy (4, 5). Ferroptosis is a novel form of programmed cell death resulting from the accumulation of iron-dependent lipid peroxides (6). Its occurrence depends mainly on increases in reactive oxygen species (ROS), phospholipids containing polyunsaturated fatty acid chains (PUFA-PLs), and iron accumulation (7). Alternatively, intracellular and intercellular signaling, as well as environmental stress, can indirectly influence ferroptosis by modulating cellular metabolic processes as well as ROS levels (8).

In 1980, the membrane protein xCT5, associated with ferroptosis, was discovered by S. Bannai et al. (9). In 2003, erastin, a compound that induces cell death through non-apoptotic pathways, was first discovered and named. It is a compound with selective lethality for Rat Adenosarcoma (RAS) (10). In 2008, RSL3 and RSL5 were also shown to induce non-apoptotic cell death. Deferoxamine (DFO) and antioxidants (e.g., vitamin E) can inhibit this process (11). Ferroptosis, a term that was first used by experts such as Dixon in 2012, is characterized as a unique type of cell death that is not apoptosis and is triggered by the compound erastin, causing cells to die via iron-dependent lipid peroxidation (12). In 2014, Wan Seok Yang et al. reported that glutathione peroxidase 4 (GPX4) plays a crucial role in regulating ferroptosis (13). Since then, studies on ferroptosis have increased. Ferroptosis is not limited to mammals; it has also been detected in plants, protozoa, and mycota (14, 15).

This manuscript provides an overview of the mechanisms underlying ferroptosis, recent advancements in the field, and future directions, particularly in the context of the ocular microenvironment and ophthalmological disorders, such as age-related macular degeneration (AMD), diabetic retinopathy (DR), retinal ischemia–reperfusion injury (RIRI), retinitis pigmentosa (RP), retinoblastoma (Rb), dry eye disease (DED), corneal injury, glaucoma, and cataract. In this manuscript, we explore how ferroptosis is related to ocular pathology, as well as its mutual metabolic effects, to establish a basis for further investigation into the pathogenesis of and methods to prevent ferroptosis in ocular diseases.

## 2 Mechanisms governing ferroptosis

### 2.1 Iron metabolism

**Figure 1:**
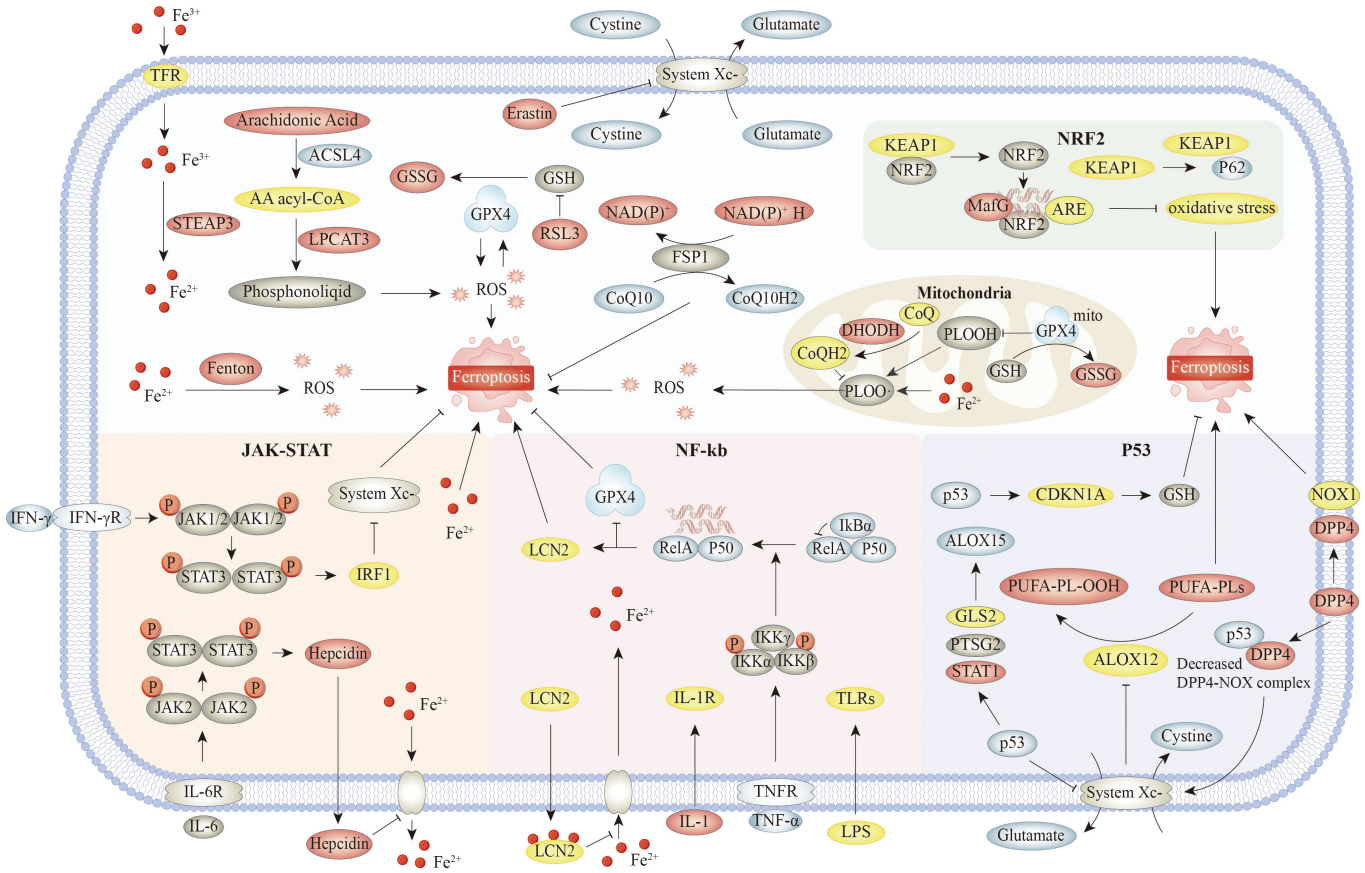
The main mechanisms and signaling pathways of ferroptosis. Specifically, the core mechanism involves accumulation of iron ions, and initiation and amplification of lipid peroxidation, as well as key regulatory pathways such as system Xc^−^ and GPX4. Associated signaling pathways include Nrf2 pathway. Nrf2 inhibits ferroptosis by upregulating the expression of GPX4 and SLC7A11 and inhibiting lipid peroxidation. NF-κB is activated by Toll-like receptor ligands, TNF, IL-1, and other stimuli. IκBα binds to NF-κB dimers and inhibits NF-κB activity under resting conditions. These signaling molecules bind to the corresponding receptors and mediate phosphorylation and subsequent degradation of IκBα. Released NF-κB dimers are transported to the nucleus and regulate transcription of target genes. On the one hand, NF-κB could decrease the transcription of antioxidant molecules such as GPX4, NQO1, and HMOX1, indicating the role of NF-κB pathway in oxidative stress. On the other hand, LIFR deletion enhanced IκBα ubiquitinated degradation and positively regulated NF-κB activation, which in turn promoted LCN2 secretion and sequestrated extracellular iron. In JAK–STAT, IFN-γ promotes ferroptosis by downregulating SLC7A11 and inhibiting system Xc^−^ through the JAK–STAT1–IRF1 axis. IL-6 upregulates hepcidin through the JAK–STAT1 axis, which can promote ferroptosis by maintaining the process outside the iron ion membrane in the cell. In addition, the JAK–STAT pathway can also suppress ferroptosis by upregulating antioxidant genes, such as GPX4, depending on the cell type and environment. On the one hand, P53 can further promote the upregulation of ALOX15 and promote ferroptosis by promoting the expression of GLS2, PTGS2, and STAT1, or can indirectly activate the function of ALOX12 by inhibiting the transcription of SLC7A11, resulting in ALOX12-dependent ferroptosis after reactive oxygen species stress. On the other hand, P53 can also inhibit the generation of lipid reactive oxygen species in cells by competitively binding DPP4 to NOX1. Meanwhile, the P53–DPP4 complex promotes the expression of SLC7A11 and CDKN1A and inhibits ferroptosis. Abbreviations: TFR, transferrin receptor; KEAP1, Kelch-like ECH-associated protein 1; NRF2, nuclear factor erythroid 2-related factor 2; GSSG, glutathione disulfide; ARE, antioxidant response element,; LPCAT3, lysophosphatidylcholine acyltransferase 3; DHODH, dihydroorotate dehydrogenase; PLOOH, phospholipid hydroperoxide; CDKN1A, cyclin-dependent kinase inhibitor 1A; NOX1, NADPH oxidase 1; DPP4, dipeptidyl peptidase-4; GLS2, glutaminase-2; IL-1R, interleukin-1 receptor; TNFR, tumor necrosis factor receptor; TNF-α, tumor necrosis factor alpha; GPX4, glutathione peroxidase 4; NQO1, NAD(P)H quinone oxidoreductase 1.

Iron homeostasis in the human body is delicately balanced; both a lack of iron and an excess of iron can be detrimental to the human body (16). Additionally, iron can readily accept and donate electrons and interconvert between iron (Fe^3+^) and ferrous iron (Fe^2+^) forms. Fe^2+^ is unstable and destroys tissue by promoting the transformation of hydrogen peroxide into free radicals that assault cell membranes, proteins, and DNA (17, 18). Iron absorption, iron transmembrane transport, and iron sequestration can affect ferroptosis (19) (**Figure 1**).

Circulating Fe^3+^ attaches to the transferrin receptor and is transported into the cell via transferrin receptor 1 (TFR1) (20). After entering the cell, Fe^3+^ is reduced and released into the labile iron pool (LIP) of the cytosol (21). Surplus iron is retained in ferritin. In the intracellular LIP, iron mostly exists as Fe^2+^. The instability and high reactivity of Fe^2+^ lead to the generation of hydroxyl radicals from excess iron by means of the Fenton reaction. These radicals can directly interact with polyunsaturated fatty acids in the cell and plasma membranes, generating substantial amounts of lipid ROS that induce cell death (22). Iron can also activate ROS-generating enzymes, such as nicotinamide adenine dinucleotide phosphate oxidase an lipoxygenase (LOX), which promote ROS generation. The overaccumulation of ROS and lipid peroxidation leads to cell membrane breakdown (23).

Iron involvement in the visual cycle was discovered by the characterization of the enzyme RPE65, an iron-dependent isomeric hydrolase critical for vision (24, 25). In the retina, iron is among the most abundant metals, and iron metabolism plays a key role in the retina (26). On the one hand, iron can contribute to the antioxidant balance by stabilizing the retina, scavenging free radicals, and shielding the retina from oxidative harm (22, 27). On the other hand, the accumulation of iron and the decrease in the cellular antioxidant protection system increase the susceptibility of the retina to oxidative stress-related cell death, which may negatively impact AMD (28). Ferroptosis induces cell death in retinal pigment epithelial (RPE) cells, retinal photoreceptor (PR) cells, and retinal ganglion cells (RGCs) and plays a role in the progression of retinal diseases such as AMD, glaucoma, and DR (29, 30). Hence, the equilibrium of iron ions is pivotal for ocular well-being.

### 2.2 Lipid peroxidation

Lipid proteins ensure the stability and normal function of cell membranes, which are destroyed when extensive lipid peroxidation occurs, ultimately leading to cell death (31). Lipid peroxide agglomeration is the core mechanism of ferroptosis (32). Free polyunsaturated fatty acids (PUFAs) function as substrates for lipid peroxidation. Their concentration and cellular distribution directly affect lipid peroxidation and ultimately the intensity of ferroptosis. Enzymes that can bind PUFAs to PLs play a decisive role in ferroptosis. PUFA-PLs are the most easily peroxidized lipids because the allylic carbons in PUFA-PLs are highly susceptible to attack by free radicals, LOX, and O_2_ (33, 34). The production of PUFA-PLs is governed by two crucial enzymes: acyl-coenzyme A synthetase long-chain family member 4 (ACSL4) and lysophosphatidylcholine acyltransferase 3 (LPCAT3) (35). Through the esterification process mediated by ACSL4, PUFAs can bind with CoA to produce PUFA-CoA derivatives. After re-esterification, LPCAT3 integrates these PUFA-CoA intermediates into the phospholipids within the plasma membrane. This process results in the generation of PUFA-PLs, such as arachidonic acid–phosphatidylethanolamine and adrenic acid–phosphatidylethanolamine. ACSL4 phosphorylation at the Thr328 site amplifies the production of PUFA-PLs, thus promoting the buildup of lipid peroxidation byproducts (32, 36). Blocking these enzymes decreases lipid peroxidation, decreasing the risk of ferroptosis.

Ferroptosis can be significantly improved by inhibiting ACSL4 and LPCAT3. For instance, liproxstatin-1 suppresses ferroptosis by lowering ACSL4 levels in the RPE–Bruch’s membrane–choroid complex, effectively halting DR progression (37, 38). PUFAs, such as docosahexaenoic acid (DHA) and eicosapentaenoic acid (EPA), are crucial for the establishment of vision and the maintenance of retinal function. DHA is the most abundant omega-3 fatty acid in PR cells and is critical for maintaining the structural integrity and functional capacity of these cells. EPA exerts anti-inflammatory effects, which can reduce the risk of AMD and other ocular diseases. Additionally, omeg-3 PUFAs protect the retina from light-induced damage by reducing oxidative stress in ocular tissues via antioxidant actions (39, 40).

### 2.3 GPX4-dependent regulatory pathway

During ferroptosis, system Xc^−^ and GPX4 can coregulate lipid oxides (41). The system Xc^−^ cystine/glutamate exchanger is a dimeric structure with a light subunit (SLC7A11) and a heavy subunit (SLC3A2) connected via disulfide linkages (42). Cystine is transported into cells through the system Xc^−^ transporter located on the membrane surface. Once inside, it undergoes reduction to form cysteine. This cysteine then serves as a substrate for two key enzymes, glutamate–cysteine ligase (GCLC) and glutathione synthetase (GSS), which work in sequence to produce the essential antioxidant glutathione (GSH). The glutathione peroxidase (GPX) family encompasses various isoforms, with GPX4 being a selenium-containing protein vital for mitigating intracellular lipid peroxidation damage in humans (43). Specifically, GPX4 can reduce PUFA-PL hydroperoxides (PUFA-PL-OOHs) to non-toxic PUFA-PL alcohols (PUFA-PL-OHs) using GSH, thereby playing a role in resistance to cellular ferroptosis (19).

Erastin and RSL3 are distinct types of agents that induce ferroptosis. Erastin blocks cystine absorption by suppressing system Xc^−^, leading to intracellular cysteine depletion, which in turn causes membrane damage and subsequent cell death (44). Notably, erastin can also activate the tumor suppressor p53, thereby inhibiting SLC7A11 and indirectly promoting the development of ferroptosis (45). RSL3 acts by inhibiting GPX4 to accumulate peroxidized phospholipids, which in turn induces the development of cellular ferroptosis (13). Cells with low GPX4 levels are more susceptible to ferroptosis than those with elevated GPX4 levels (11). GPX4 can maintain the healthy state of retinal cells by protecting cell membranes from oxidative damage (46). In GPX4-overexpressing transgenic mice, retinal integrity and function are preserved across multiple oxidative stress-induced degeneration models (47, 48). Under ocular conditions such as those of DR, a reduction in GPX4 levels can result in increased oxidative stress, consequently impacting the functionality and viability of PR cells (49). GPX4 maintains redox homeostasis and protects RPE cells (RPECs), photoreceptors, and RGCs against glutamate-induced cytotoxicity. Collectively, these findings indicate that GPX4 overexpression markedly attenuates oxidative stress-driven retinal degeneration and preserves photoreceptor outer-segment architecture. Boosting GPX4 activity, therefore, holds promise as a precise therapeutic strategy to delay or even reverse AMD progression.

### 2.4 GPX4-independent regulatory pathway

Although GPX4 acts as a central suppressor of ferroptosis, three other ways to inhibit ferroptosis have been found, irrespective of GPX4. Ferroptosis inhibitor protein 1 (FSP1) is an effective antiferroptosis factor and a coenzyme Q (CoQ) oxidoreductase (50). Mechanistically, FSP1 exhibits nicotinamide adenine dinucleotide + hydrogen (NADH) ubiquinone reductase activity. This mechanism facilitates the conversion of ubiquinone (CoQ10) into its reduced form, ubiquinol (CoQ10H_2_), which effectively curbs the production of lipid free radicals. Another pathway involves enhancing vitamin E regeneration, which in turn suppresses lipid peroxidation and prevents ferroptosis. The FSP1–CoQ10–NAD(P)H axis serves as a key cellular defense against oxidative stress and an alternative pathway for inhibiting ferroptosis (51).

In 2019, Bersuker et al. reported that tumor cells lose resistance to the ferroptosis inducer RAS-selective lethal 3 (RSL3) when they are depleted of FSP1, thereby becoming more susceptible to ferroptosis (52). FSP1 is crucial for corneal and retinal repair, enhancing tissue regeneration through cell cohesion and facilitating movement (53). Moreover, FSP1 supports RPE cells by ensuring that they remain robust and well-aligned, which are crucial for maintaining photoreceptor functionality. Furthermore, FSP1 could play a role in controlling abnormal vascular growth and scar formation in the context of vascular diseases such as DR (54). FSP1 is a vital player in fixing both the cornea and the retina. When the cornea becomes injured, FSP1 forms a temporary framework that supports the regeneration of the outer layer, which speeds up the healing process. Moreover, it is crucial in the protection of RPECs. In cases where blood vessels malfunction, like in DR, FSP1 may play a role in controlling the growth of extra blood vessels and the formation of tough scar tissue by communicating with growth factors and the cell surface receptors, which in turn affects how the extracellular matrix changes and the series of events that lead to new blood vessel formation. Furthermore, FSP1 may also be a factor in AMD by influencing the growth of abnormal blood vessels behind the retina and the development of scar tissue.

The dihydroorotate dehydrogenase (DHODH) pathway generates reduced coenzyme Q (CoQH_2_) within the inner mitochondrial membrane, which plays a key role in suppressing ferroptosis. By functioning as a potent radical-trapping antioxidant, CoQH_2_ effectively blocks lipid peroxidation—a critical mechanism that halts the progression of ferroptosis. In this process, DHODH functions concurrently with the mitochondrial GPX4, separate from the cytoplasmic GPX4 and FSP1 (55). In an experiment in which a stable DHODH knockout human corneal epithelial cell (HCEC) line was established, the balance of expression between DHODH and GPX4 closely regulated cellular ferroptosis homeostasis (56). Recently, KIO-101 ophthalmic solution, a topical DHODH inhibitor, has been shown to be safe and effective at reducing conjunctival hyperemia at low and medium doses over a 12-day period in patients with herpesvirus (HV) infection and conjunctival hyperemia (57). The enzyme DHODH acts as a safeguard against ferroptosis in eye tissues like the cornea and retina without relying on GPX4, but when this protective pathway is diminished, particularly when disrupted by oxidative stress, it could lead to damage of corneal and retinal cells by facilitating the progress of ferroptosis. In addition, researchers have deciphered the link between DHODH’s activity and several serious eye conditions, including AMD, DR, and abnormal blood vessel growth in the cornea.

Guanosine-5′-triphosphate (GTP) cyclohydrolase-1 (GCH1), a key biosynthetic enzyme, regulates tetrahydrobiopterin (BH4) production. BH4 serves as a coenzyme for critical neurotransmitter (e.g., dopamine) and nitric oxide synthesis pathways (58) and coregulates ferroptosis through two mechanisms. First, GCH1 produces the lipophilic antioxidant BH4, which functions similarly to CoQ10 to prevent lipid peroxidation. Second, GCH1 remodels the lipid membrane environment by promoting the uptake of PUFA-PL, an inducer of ferroptosis, while increasing CoQ10 (CoQ10H_2_) levels, thereby counteracting lipid peroxidation. In ophthalmology, the role of GCH1 could be crucial for maintaining retinal integrity and eliminating specific eye disorders, such as AMD and other retinal degenerative diseases (59).

### 2.5 JAK–STAT

Janus kinases (JAKs) are intracellular non-receptor tyrosine kinases that include JAK1, JAK2, JAK3, and TYK2. These kinases are widely distributed across various tissues and cells. Signal transducers and activators of transcription (STATs) act as substrates for JAKs and function as transcription factors. The STAT family includes genes such as STAT1, STAT2, and STAT3. Following phosphorylation by JAKs, STATs translocate to the nucleus to modulate gene transcription. The JAK–STAT signaling pathway is defined by these key components and is distinguished by their diverse interactions (60). When IL-6 acts on its receptor, it causes the phosphorylation of JAK2, leading to the phosphorylation of STAT3 and resulting in an increase in hepcidin expression. When excess hepcidin is transferred to the extracellular space, it inhibits the release of Fe^2+^ from the cell, ultimately leading to ferroptosis. IFN-γ acts on the IFN-γ receptor, resulting in the phosphorylation of JAK1/2, which triggers the phosphorylation of STAT1 and leads to an increase in interferon regulatory factor 1 (IRF1) expression, which inhibits the function of SLC7A11 with SLC3A2 (system Xc^−^), ultimately leading to ferroptosis (61).

In ophthalmology, corticosteroids (CSs) are a common treatment for patients with non-infectious uveitis (NIU). Potentially severe side effects may result from prolonged CS use. JAK/STAT inhibitors can provide additional therapeutic options for patients with NIU. Primary Sjögren’s syndrome (pSS) is a systemic autoimmune disorder that mainly affects exocrine glands and contributes to DED development (62). JAK1 may influence pSS progression, offering therapeutic potential (63).

### 2.6 NF-κB

The classical transcription factor NF-κB was identified more than three decades ago (64). A growing body of research has indicated that ferroptosis is linked to the NF-κB signaling pathway. At rest, IκBα suppresses NF-κB by binding to its dimers. Upon external stimulation, signaling molecules bind to their receptors, leading to the activation of the IKK complex (via phosphorylation of IKKβ), which phosphorylates IκBα, causing its degradation and dissociation from NF-κB dimers. In certain pathological contexts, the nuclear translocation of NF-κB dimers can suppress the expression of antioxidant-related genes such as GPX4, thereby promoting ferroptosis (61).

Wen-Jing Liang et al. speculated that high-mobility group box 1 (HMGB1) could trigger apoptosis in retinal endothelial cells via the NF-κB pathway, leading to ischemic regions and subsequently triggering compensatory blood vessel growth and enhanced new vessel formation (65). In the mammalian retina, NF-κB signaling promotes glial reactivity and inhibits glia-mediated neuronal regeneration (66). Kaiwen Jiang et al. found that fosinopril (FOS), a TLR4 inhibitor, can inhibit NF-κB signaling in both *in vivo* and *in vitro* models. This inhibition can regulate diabetic DED, providing new therapeutic options for diabetic DED (67). Increased high-temperature requirement for A serine peptidase 1 (HTRA1) levels activate NF-κB protein synthesis, whereas HTRA1 knockdown downregulates NF-κB protein expression. Elevated NF-κB expression is a known risk factor for AMD (68).

### 2.7 Nrf2

With respect to nuclear transcription factors, such as Nrf2, Sun et al. reported the role of the p62–Keap1–Nrf2 antioxidant signaling cascade in safeguarding hepatoma cells against ferroptosis (69). First, the p62-mediated degradation of Keap1 activates Nrf2. Activated Nrf2 translocates to the nucleus, where it triggers the expression of antioxidants such as glutathione redox system components, enzymes that regulate iron metabolism, and other pertinent molecules, which can inhibit ferroptosis. Nrf2 cannot undergo the above manipulations when it is degraded by ubiquitination. Second, the antioxidant response element (ARE) acts as a pivotal modulator of Nrf2-driven SLC7A11 activation, with its relationship to Nrf2 occurring irrespective of p53 involvement (70). Activating the p62–Keap1–Nrf2 signaling pathway increases systemic Xc^−^ levels, thereby mitigating lipid peroxide accumulation and inhibiting ferroptosis (71).

The eye serves as a key marker of oxidative damage (72, 73). Oxidative stress is involved in numerous eye diseases. The Keap1–Nrf2–ARE pathway serves as an antioxidant pathway. Many studies have utilized Nrf2-activating drugs to evaluate the cytoprotective effects of Nrf2 in retinal tissue, particularly in rescuing RPECs from oxidation-induced injury and death. Xu et al. enhanced bioavailability by combining quercetin with phospholipids. This approach resulted in a nearly 80% increase in the proliferation of RPECs, decreased ROS and malondialdehyde (MDA) levels, and inhibited apoptosis. The observed outcomes were facilitated through increased Nrf2 protein transport and the activation of its target genes, including heme oxygenase-1 (HO-1) and NAD(P)H quinone oxidoreductase 1 (NQO1) (74). Nrf2–Keap1 signaling is central to the complex pathology of DR (75). Empirical research has demonstrated that elevated glucose levels in diabetic mice blunt Nrf2-mediated protection; indeed, compared with wild-type mice, Nrf2-deficient diabetic rodents exhibited significantly greater retinal superoxide levels after 5 weeks of diabetic modeling (75). von Otter et al. reported that Nrf2 gene mutations may promote cataract progression but do not invariably increase the likelihood of cataract onset (76). The gene loci of Nrf2 and Keap1 were analyzed in 489 cataract patients of European ancestry. One Nrf2 haplotype, GAAAA, was associated with the progression of cataract formation. Specifically, this haplotype was significantly linked to an earlier onset of cataracts by approximately 4 years. However, the GAAGAGGC haplotype of the Nrf2 gene delayed the need for cataract surgery by 4 years (77). This undoubtedly provides new research directions for addressing the challenges posed by an aging population and the increasing demand for cataract surgery. Activating Nrf2 not only scavenges ROS but also inhibits inflammation and promotes epithelial repair, providing an ideal target for treating DED (78).

### 2.8 p53

The p53 gene was discovered in 1979, and studies have shown that p53 serves dual functions in ferroptosis regulation (79). On the one hand, it enhances ALOX15 expression by suppressing SLC7A11 expression, thereby deactivating ALOX12. Furthermore, it upregulates the expression of metabolic genes such as SAT1 and glutaminase-2 (GLS2); their combined activity increases lipid–ROS production and GSH turnover, thereby sensitizing cells to ferroptosis (80, 81). On the other hand, p53 suppresses ROS generation in cellular lipids by competitively binding to dipeptidyl peptidase-4 (DPP4), thereby preventing NOX1 activation. Moreover, SLC7A11 and cyclin-dependent kinase inhibitor 1A (CDKN1A) gene expression is stimulated by the p53–DPP4 interaction, thereby inhibiting ferroptosis (82). p53 activation does not noticeably affect GPX4 activity, suggesting that it does not induce ferroptosis via GPX4 (71). Recent research has indicated that wild-type p53 can promote ferroptosis in certain contexts (e.g., by repressing SLC7A11), whereas selected gain-of-function mutants may also sensitize tumor cells to ferroptosis, although many mutants confer resistance (70).

p53 can be rapidly activated to induce cell cycle arrest when retinal cells are subjected to photodamage, hypoxia, or metabolic stress, enabling DNA repair (83). When injury is extreme, p53 triggers programmed cell death to prevent the growth of damaged cells and mitigate tumor development. Wai Kit Chu presented evidence that targeting the MDM2–p53 pathway could help elucidate the pathogenesis of pterygium and develop new treatments to reduce the postoperative recurrence rate (84). Ying Chen et al. revealed a mechanism by which p53 increases FoxO3a ubiquitination levels through ubiquitin-conjugating enzyme E2 L6 (UBE2L6) and promotes aging in diabetic retinal endothelial cells, suggesting a novel therapeutic focus for the mitigation and treatment of DR (85). Moreover, p53 plays a role in modulating cellular metabolism and influences the progression of ocular disorders such as DR through its influence on blood glucose regulation and fatty acid metabolism (86).

### 2.9 AMPK

Energy stress is a metabolic condition characterized by ATP depletion and elevated AMP levels. Energy stress triggers the activation of AMP-activated protein kinase (AMPK). AMPK phosphorylates downstream targets to promote ATP production (87). The inhibition of ATP depletion restores energy balance. AMPK acts as a core regulator of ATP balance in cells and can have diametrically opposite effects on substrate-dependent ferroptosis. AMPK-induced BECN1 phosphorylation promotes ferroptosis by either suppressing SLC7A11 function or triggering autophagy (88, 89). In contrast, mitochondrial energy stress may suppress ferroptosis through AMPK-mediated acetyl-CoA carboxylase alpha (ACACA) phosphorylation. Experimental results have revealed a lack of significant ferroptosis when glucose concentration was insufficient, even when erastin was added, demonstrating that the energy stress-mediated AMPK pathway could inhibit ferroptosis (87).

AMPK also plays a role in controlling mitochondrial formation and inflammation. It promotes mitochondrial quality control, helping to replace dysfunctional mitochondria, which is essential for maintaining the health of eye cells. Additionally, AMPK can control the progression of ocular diseases by inhibiting the mTORC1 pathway (90, 91). Yuli Guo et al. reported that the AMPK agonist metformin could ameliorate hyperglycemia-induced meibomian gland dysfunction (MGD), demonstrating that AMPK may be a therapeutic target for diabetes-induced MGD (92). Fangli Peng et al. found that AMPK/MFF signaling plays a key role in DED progression by actively promoting mitochondrial fission and mitophagy. Suppressing excessive mitochondrial fragmentation helps mitigate oxidative stress and inflammation associated with DED, offering promising therapeutic targets for clinical intervention. These findings establish a scientific foundation for the development of novel DED treatment strategies (93).

### 2.10 HSPs

Heat shock proteins (HSPs) are popular representative protein families of chaperones and have traditionally been divided into nine subfamilies according to molecular weight (94). They mitigate cellular stress, provide antioxidant protection, and steer immune activity toward an anti-inflammatory state, collectively constituting key mechanisms against ferroptosis (95). The phosphorylation of heat shock protein B1 (HSPB1), also referred to as HSP25 or HSP27, mediated by protein kinase C (PKC), limits cytoskeleton-mediated iron uptake. This action restricts the death of iron-tropic cancer cells by reducing iron uptake, thereby decreasing ferroptosis (96). In addition, the phosphorylation of HSPB1 by PKC triggers the increased expression of ferritin light chain (FTL) and ferritin heavy polypeptide 1 (FTH1), both of which are important components associated with ferritin. Increased ferritin expression decreases cellular iron levels and mitigates the formation of ROS in lipids. HSPB1 also regulates TFR1 expression.

Activating the expression of heat shock protein family A (HSP70) member 5 (HSPA5) can prevent erastin-induced GPX4 degradation through the formation of HSPA5–GPX4 protein complexes, thereby inhibiting ferroptosis (97). In contrast, heat shock protein 90 can induce ferroptosis by phosphorylating receptor-interacting protein 1 (RIP1), a key regulator of necroptosis, and inhibiting GPX4 (98).

Heat shock proteins play a protective role in ocular diseases under oxidative, inflammatory, thermal, UV, or metabolic stress. T-cell reactions targeted at heat shock proteins contribute to glaucomatous neuronal degeneration (99, 100). In cases of glaucoma and neurodegenerative eye disorders, HSPs serve as a protective barrier for RGCs against mechanical stress and neurotoxicity associated with these diseases (101, 102). HSPB1 expression increases sharply when the retina is subjected to injury, such as ischemia, oxidative stress, trauma, or ocular hypertension. On the one hand, it maintains cell structural integrity; on the other hand, it inhibits apoptotic signal amplification, thereby increasing the survival rate of retinal cells under toxic stimuli (103). However, Alyce Alven et al. reported increased levels of HSPB11 and HSP60 in the tear film of DED patients after acute exposure to dry conditions, a trend that was consistently noted (104).

### 2.11 NADPH

NADPH plays a crucial role in maintaining cellular redox balance, and each NADPH molecule provides two electrons to ensure the integrity of antioxidant defense mechanisms (105). Glutathione reductase (GR), FSP1, NAD(P)H quinone dehydrogenase 1 (NQO1), and thioredoxin reductase (TR) maintain the reduced state of retinol, GSH, CoQ10, and vitamin K molecules through the electron supply of NADPH, effectively inhibiting the occurrence of phospholipid peroxidation (106, 107).

NADPH promotes both phospholipid synthesis and the function of heme-dependent NADPH oxidases (NOXs). These specialized enzymes transfer electrons from cytoplasmic NADPH to generate ROS that initiate lipid peroxidation. Concurrently, the same pool of NADPH fuels the GPX4, thioredoxin reductase, and lipid-remodeling pathways, both neutralizing peroxides and synthesizing protective phospholipids (PUFA-OHs) to counteract ferroptosis. Under steady-state conditions, NADPH is recruited primarily for ferroptosis defense, thereby potentially inhibiting its support of the pro-ferroptosis pathway (107). These findings indicate that NADPH plays a critical role in ferroptosis-related chemical processes (108).

NADPH functions in biosynthetic reactions, as well as maintains a reducing environment within the cell, and is crucial for the dark-phase reactions of photosynthesis in mitigating oxidative stress. In ophthalmology, ensuring NADPH levels and functionality is crucial for safeguarding ocular health and managing eye disorders (109). NADPH oxidase 4 (NOX4) contributes to DED progression by modulating IL-1β, NLRP3, and MUC5AC expression; inhibiting NOX4 using drugs and inhibitors may improve DES (110). The targeted suppression or blockage of NADPH oxidase 2 (NOX2) function significantly alleviates oxidative damage in the retina, corrects immune system imbalances, prevents damage to the inner blood–retinal barrier (iBRB), and mitigates neurovascular unit (NVU) impairment. Additionally, it reduces RGC death and optic nerve (ON) axon deterioration caused by high intraocular pressure (H-IOP). These findings suggest a promising therapeutic strategy for managing glaucoma (111).

There are many ferroptosis inhibitors. AA-861, zileuton, and PD-146176 inhibit ferroptosis by reducing the generation of LOX-induced lipid peroxidation products. ATV regulates iron content, ROS levels, and GSH to reduce lipid peroxidation. CoQ10 acts on FSP1 and GPX4 to reduce cholesterol metabolism disorders and inhibit ferroptosis. Deferoxamine and other iron chelators work by reducing iron content. Ferrostatin-1 (Fer-1) regulates ROS and the GSH/GPX4 axis to reduce lipid peroxidation. Lipstatin-1 (Lip-1) inhibits ferroptosis by suppressing the activation of lipid metabolism. Naringenin increases cellular sensitivity to ferroptosis by enhancing the SIRT1/FOXO3a signaling pathway. SAK increases the expression of antioxidant genes by activating the NRF2 signaling pathway. Selenium, sodium, and selenite increase the expression of antioxidant genes by acting on GPX4 and system Xc^−^. Tangeretin reduces lipid peroxidation by acting on GPX4 and NRF2. Taurine reduces iron content by regulating the OGT/GPX4 signaling pathway. Trolox and VitE reduce lipid peroxidation by scavenging hydroxyl radicals and regulating GPX4 (**Table 1**).

**Table 1:** The inhibitors of the ferroptosis pathway.

Reagent	Ferroptosis-related targets	Mechanism	References
AA-861, zileuton, PD-146176	LOX-induced lipid peroxidation	Decreasing the generation of lipid peroxides	(112)
ATV	Iron content, ROS levels, GSH	Decreasing lipid peroxidation	(113)
CoQ10	FSP1, GPX4	Decreasing dysregulation of cholesterol metabolism	(114)
Deferoxamine, deferiprone, piroctone olamine	Iron chelator	Decreasing iron levels	(115)
Fer-1	Cytosolic and lipid ROS, GSH/GPX4 axis	Decreasing lipid peroxidation	(116)
Lip-1	Lipid peroxidation	Decreasing the activation of lipid metabolism	(117)
Naringenin	The SIRT1/FOXO3a signaling pathway	Increasing cellular susceptibility to ferroptosis	(118)
SAK	The NRF2 pathway	Increasing the expression of antioxidant genes	(119)
Selenium, sodium, selenite	GPX4, system Xc^−^	Increasing the expression of antioxidant genes	(120)
Tangeretin	GPX4, NRF2	Decreasing lipid peroxidation	(121)
Taurine	OGT/GPX4 signaling	Decreasing iron levels	(122)
Trolox	Hydroxyl radicals	Decreasing lipid peroxidation	(123)
VitE	Hydroxyl radicals, GPX4	Decreasing lipid peroxidation	(124)

Fer-1, Ferrostatin-1; Lip-1, liproxstatin-1; ATV, atorvastatin; VitE, vitamin E; SAK, sakuranetin; GSH, glutathione; CoQ10, coenzyme Q10; FSP1, ferroptosis suppressor protein 1; System Xc^−^ (xCT), cystine/glutamate antiporter; LOX, lipoxygenase; ROS, reactive oxygen species; GPX4, glutathione peroxidase 4.

## 3 Ferroptosis and other forms of cell death

Ferroptosis represents an iron-driven cell elimination process that is distinct from apoptosis, marked by the excessive buildup of lipid peroxides (125). This biological mechanism plays a pivotal role in the development and progression of numerous diseases. When it comes to cellular characteristics, ferroptosis causes mitochondria to shrink, with their cristae either diminishing or completely vanishing, while also increasing membrane density. Additionally, the cell membrane becomes fragmented and develops blebbing, although the nucleus remains largely unchanged in appearance (126).

### 3.1 Ferroptosis and apoptosis

Apoptosis serves as a natural, programmed mechanism in the course of development, effectively eliminating unnecessary cells for an organism to function well. This biological process is key to maintaining balance within the body by selectively removing cells as needed. Additionally, it acts as a safeguard, eliminating compromised, infected, or potentially cancerous cells, and it plays a vital part in shaping and sustaining a robust immune system (127). During programmed cell death, cells systematically contract and develop blebs, bulbous protrusions resembling bubbles along their outer membrane. Concurrently, nuclear DNA disintegrates while certain internal structures, including the endoplasmic reticulum, fragment into smaller components. The cell ultimately divides into numerous membrane-encased packets known as apoptotic bodies, which are subsequently cleared by phagocytic cells like macrophages without triggering any inflammatory response (128).

Cancer treatment has recently capitalized on the combined impact of ferroptosis and apoptosis, with researchers successfully engineering a two-dimensional catalytic nanozyme known as Cu_2_Mo_3_O_8_ nanosheets (CMO) NSs. This innovative compound not only boosts ROS production to stimulate ferroptosis but also mimics glucose oxidase activity, essentially cutting off the tumor’s nutrient supply by breaking down glucose and generating H_2_O_2_. In addition, CMO NSs facilitate calcium release alongside ROS-induced buildup of endogenous calcium, causing calcium overload that leads to mitochondrial malfunction and apoptosis. This multi-pronged approach makes CMO NSs a promising candidate for antitumor therapy (129). Under specific conditions, ferroptosis and apoptosis can also achieve mutual transition (130). Harnessing the synergy between ferroptosis and apoptotic pathways holds immense potential for advancing cancer treatment strategies. By focusing on this interconnected cell death network, researchers are working toward a more holistic blueprint to curb tumor progression and potentially tackle other conditions linked to these mechanisms. This exciting frontier continues to be a focus of scientific exploration, with investigations delving deeper into its therapeutic possibilities (131).

In the eye field, 24 hours after retinal ischemia–reperfusion, key indicators of ferroptosis, such as lipid peroxidation and the downregulation of GPX4, rise in tandem with apoptosis markers, including cleaved caspase-3 and TUNEL-positive cells (132). Treatment with the ferroptosis inhibitor ferrostatin-1 can simultaneously reduce both types of cell death, indicating that they jointly mediate acute damage. In a chronic ocular hypertension mouse model, RGCs exhibit both ferroptosis (characterized by the upregulation of ACSL4 and increased lipid ROS) and apoptosis (marked by Bax/Bcl-2 imbalance and the release of cytochrome *c*). The iron chelator deferoxamine or the GPX4 activator RSL3 can concurrently reduce both types of death and decrease RGC loss by approximately 40%. In corneal epithelial cells stimulated by hyperosmosis or cigarette smoke extract (CSE), both ferroptosis and apoptosis occur simultaneously (133). The combined inhibition of Fer-1 and z-VAD more significantly restores cell viability than either agent alone. Overall, ferroptosis and apoptosis form a connection through an interplay involving ROS, mitochondrial dysfunction, and inflammation. Targeting both ferroptosis and apoptosis together has shown superior efficacy compared to single-pathway intervention, providing a new and precise therapeutic strategy for the treatment of ocular diseases.

### 3.2 Ferroptosis and pyroptosis

Pyroptosis is a cell death mode in which cell membrane Gasdermin family proteins form pores that lead to the release of cell contents and cell rupture (134). Pyroptosis is an important defense mechanism in the immune system that can clear pathogen-infected cells and trigger inflammatory responses. Inflammasomes and GasderminD (GSDMD) play critical roles in pyroptosis (135).

Programmed cell death (PCD) manifests in various guises, each with its own distinctive features shaped by unique molecular pathways, and these forms often cross paths in complex ways (136). Interestingly, ferroptosis and pyroptosis may be somehow connected, although the exact nature of their relationship remains to be elucidated. Recent experiments, however, have revealed their antagonistic relationship by tracking how HMGCR shifts its position during cell death and how BRCC36 controls both processes by removing ubiquitin from HMGCR. Moving forward, diving deeper into the precise mechanics of how ferroptosis and pyroptosis interact could pave the way for more targeted and effective disease treatments (137).

Inflammasomes are an important marker of pyroptosis. In recent years, the intricate relationship between ferroptosis and inflammasomes has been progressively unveiled by a growing body of research. Specifically, iron overload serves as a catalyst for lipid peroxidation, which subsequently triggers a substantial surge in the production of ROS. This surge in ROS acts as a driving force that propels the assembly and activation of inflammasomes, such as NLRP3 (138). Once activated, these inflammasomes orchestrate the release of proinflammatory cytokines IL-1β and IL-18 (139). Moreover, the formation of GSDMD pores, which are also induced by the activated inflammasomes, further exacerbates the accumulation of iron and oxidative damage within the cells. Collectively, these events culminate in a cascade amplification of cell death and inflammatory response. In the context of RPECs, which play a pivotal role in maintaining retinal homeostasis, the targeted knockdown of cGAS or STING has been shown to effectively disrupt a critical sequence of events (140). This sequence includes iron accumulation, leakage of mitochondrial DNA, and subsequent NLRP3 activation. By interrupting this pathway, the levels of ferroptosis and inflammatory factors are significantly reduced by more than 50% in photochemical injury models. This reduction underscores the potential of targeting this intersection to simultaneously block cell death and the inflammatory storm, thereby offering a novel and combined intervention strategy for retinal degenerative diseases (141).

### 3.3 Ferroptosis and necroptosis

Historically, apoptosis was widely regarded as the sole mechanism of programmed cell death, with necrosis dismissed as a chaotic and unregulated phenomenon. The tide began to turn in 1988 when research revealed that TNF-α could trigger both apoptotic and necrotic cell death, suggesting that necrosis may not be a mere accident but a controlled process. A pivotal study by Holler’s team later identified receptor-interacting serine/threonine-protein kinase 1 (RIPK1) as a key player in Fas-mediated cell death. The discovery of necrostatin-1 (NEC-1), a specific inhibitor of RIPK1 kinase activity, which effectively halts death receptor-induced necrosis, further cemented the idea that this type of cell demise is under strict regulation. Dubbed “necroptosis”, this programmed necrotic pathway is marked by plasma membrane disruption and shares upstream components, such as RIPK1, with caspase-8-dependent apoptosis. Its morphological hallmarks include cell swelling and ballooning, bubble-like protrusions, and rupture of the plasma membrane (142).

Necroptosis and ferroptosis differ in morphological characteristics and regulatory mechanisms. These two modes of cell death can coexist in diseases and jointly drive pathological progression (143). Studies have shown that the simultaneous inhibition of both forms of cell death can enhance therapeutic efficacy against complex necrosis-related disorders. However, targeting both types with a single compound remains challenging because they involve distinct molecular pathways.

High-level ROS not only act as a “catalyst” for ferroptosis but also directly phosphorylate RIPK3, thereby triggering necroptosis. Once the RIPK3–MLKL pores are formed, they further promote the Ca^2+^/Na^2^ influx and a mitochondrial ROS burst, which in turn exacerbates ferroptosis (144). In ARPE-19 cells, the ferroptosis inducer RSL3 suppresses GPX4 and simultaneously significantly increases RIPK3 phosphorylation. Conversely, the necroptosis inducer shikonin induces mild lipid peroxidation, indicating that the two pathways converge at RIPK3. Treatment with the RIPK1 inhibitor Nec-1 concurrently blocks both necroptosis and ferroptosis, and its protective effect is significantly superior to that of ferrostatin-1 or z-VAD alone (145). In a retinal ischemia–reperfusion model, the intravitreal administration of Nec-1 or the iron chelator deferoxamine increases RGC survival by 45% and 38%, respectively. However, their combination raises survival to 62%, suggesting synergistic protection through dual-pathway inhibition (132). In a dry-eye corneal epithelial model, hyperosmotic stress triggers both ferroptosis and RIPK3–MLKL phosphorylation, which is associated with necroptosis. Ferrostatin-1 + Nec-1 combined inhibitors restore more than 80% of epithelial integrity, significantly outperforming either agent alone. Ferroptosis and necroptosis thus form a self-amplifying, reciprocal loop mediated by ROS, RIPK3, and lipid peroxidation in ocular diseases, with RIPK3 serving as the molecular intersection (146). Combined blockade of necroptosis and ferroptosis provides synergistic protection and offers a novel multi-target intervention strategy for retinal degenerative diseases and corneal epithelial injury (132).

## 4 Ferroptosis in the ocular microenvironment

**Figure 2:**
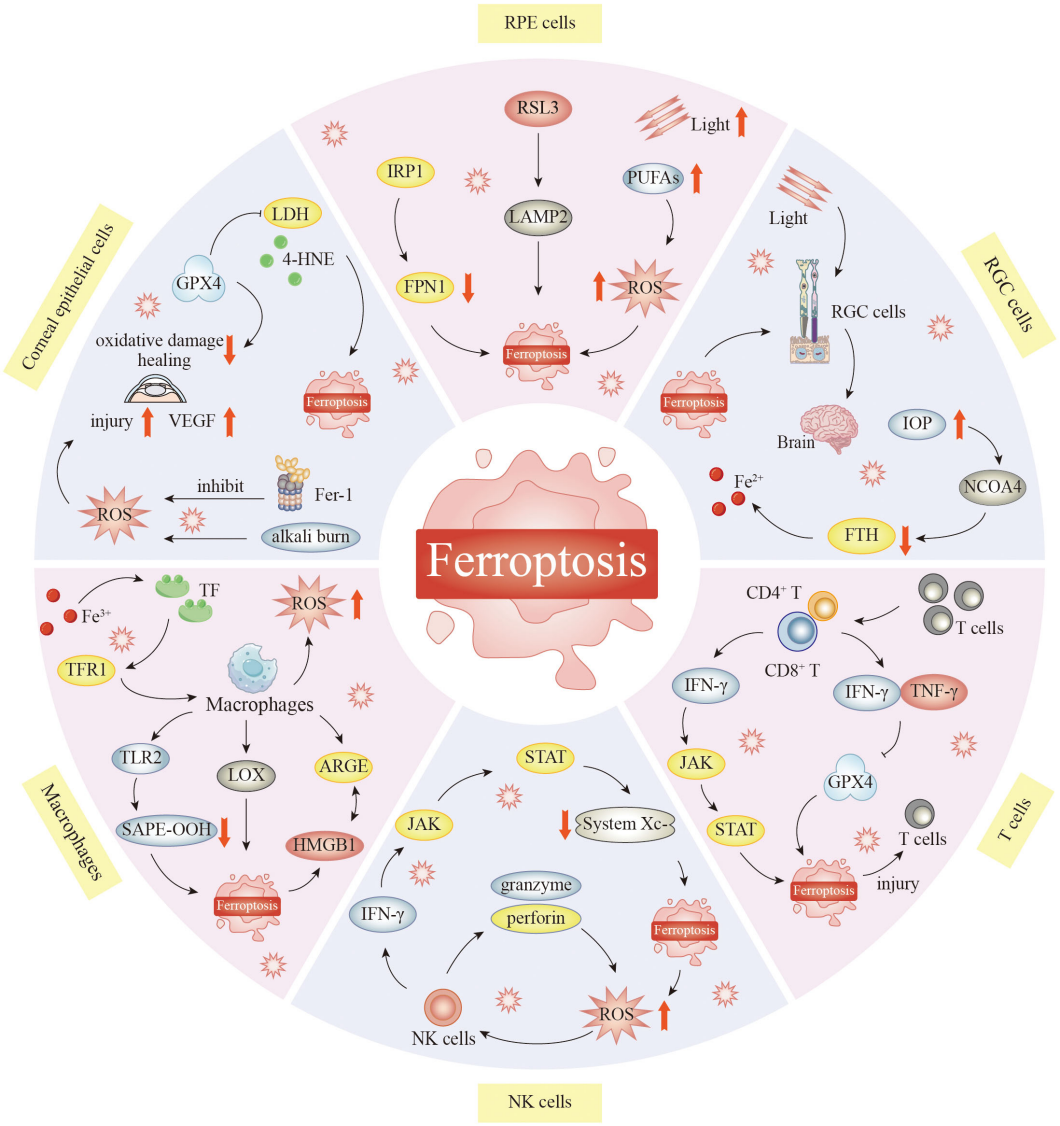
The role of ferroptosis in the ocular microenvironment. Ferroptosis plays an important role in keratocyte injury. Inhibition of ferroptosis can protect corneal epithelial cells from oxidative stress-induced cell death in the absence of GPX4. Corneal alkali burn activates ferroptosis. Reactive oxygen species attack mitochondria and jointly promote the occurrence of ferroptosis in corneal tissue. In RPECs, GPX4 inhibitors 1s and RSL3 can elevate LAMP2, resulting in ferroptosis. Supplementation with cysteine and glutamine restored GSH function, thereby inhibiting ROS-induced death in LAMP2 knockout RPEC. Reduction of NCOA4 leads to increased degradation of FTH1 and increased Fe^2+^ content in the retina. This significantly increases iron ion levels, leading to RGC damage. Macrophages can clear cells that undergo ferroptosis. HMGB1 released from ferroptosis cells can interact with AGRE on macrophages and mediate inflammatory responses in macrophages. In addition, TLR2 on macrophages recognizes and binds SAPE-OOH on the surface of ferroptosis cells to help clear ferroptosis cells. Macrophages are central to the regulation of iron homeostasis, and normally, the body can maintain the stability of iron content. When this stabilization is broken, abnormal iron metabolism may oversupply the active form of iron, ultimately leading to the development of ferroptosis. Iron accumulation and excess can lead to increased oxidative stress in natural killer cells, which triggers ferroptosis. T cells themselves may also develop ferroptosis, which may attenuate their immune response. T cells lacking GPX4 rapidly accumulate membrane lipid peroxides, leading to ferroptosis, and CD8^+^ cytotoxic T cells can eliminate tumor cells by inducing ferroptosis, which may enhance the effect of cancer immunotherapy. Abbreviations: RSL3, RAS-selective lethal 3; IRP1, iron-regulatory protein 1; PUFAs, polyunsaturated fatty acids; LDH, lactate dehydrogenase; LAMP2, lysosome-associated membrane protein 2; 4-HNE, 4-hydroxynonenal; VEGF, vascular endothelial growth factor; IOP, intraocular pressure; NCOA4, nuclear receptor coactivator 4; CD4^2^ T, CD4-positive T lymphocytes; CD8^2^ T, CD8-positive T lymphocytes; TLR2, Toll-like receptor 2; SAPE-OOH, 1-stearoyl-2-arachidonoyl-sn-glycero-3-phosphoethanolamine-hydroperoxide; HMGB1, high-mobility group box 1; NK cells, natural killer cells; GPX4, glutathione peroxidase 4; RPECs, retinal pigment epithelial cells; GSH, glutathione; ROS, reactive oxygen species; FTH1, ferritin heavy polypeptide 1; RGC, retinal ganglion cell.

In the ophthalmic microenvironment, ferroptosis affects both structural and immune cells (**Figure 2**). Corneal epithelial cells lose the GPX4 shield under hyperosmotic conditions, and lipid ROS rapidly break through the cell membrane and induce corneal opacity. RPECs experience a rapid iron surge from ingesting outer photoreceptor segments high in PUFAs, leading to geographic atrophy. Once GSH is depleted, RGCs undergo apoptosis and signal transduction to amplify retinal degeneration in glaucoma or ischemia–reperfusion scenarios because of the dual effects of mitochondrial iron deposition and hypoxia. Moreover, infiltrating T cells upregulate Nox4 in the inflammatory microenvironment and produce a large number of extracellular ROS, such that they themselves undergo ferroptosis, thereby weakening immune surveillance. The activity of the natural killer cell-dependent and iron-dependent perforin–granzyme pathway is blocked by iron overload, thereby decreasing the killing function of these cells. After M1 polarization, macrophages release large amounts of Fe^2+^ via ferritin autophagy mediated by nuclear receptor coactivator 4 (NCOA4), which not only promotes ferroptosis but also causes the further release of ferroptosis-promoting factors into the cornea, RPE, and RGCs, forming a vicious cycle in the ocular microenvironment. Targeted blockade of this transcellular ferroptotic network is emerging as an innovative approach for safeguarding the visual ecosystem.

### 4.1 Ferroptosis in corneal epithelial cells

The cornea is part of the optometric pathway, and corneal damage occurs when the protective function and structural integrity of the corneal epithelium are compromised (147). This disruption can result in partial or complete loss of corneal epithelial cells. Patients can present with widespread punctate lesions or surface erosions of the cornea, as well as persistent detachment and epithelial damage, and inflammatory reactions, ultimately endangering vision (148, 149). Corneal epithelial cells face greater susceptibility to oxidative damage (150).

GPX4 serves as a pivotal regulator of ferroptosis-driven processes. Nonetheless, it is a crucial antioxidant enzyme that is integral to maintaining the balance of redox reactions and fostering the repair of corneal epithelial cells. GPX4 does this by transforming harmful lipid peroxides into harmless lipid alcohols, thereby accelerating the healing process (151). Lower GPX4 levels trigger oxidative damage and cell toxicity, resulting in reduced corneal epithelial cell survival and impaired wound healing ability. Sakai introduced specific siRNAs against catalase, GPX4, superoxide dismutase 1 (SOD1), and SOD2 into HCECs (150) and found that lactate dehydrogenase (LDH) release was significantly elevated in the GPX4-, SOD1-, SOD2-, and CAT-knockdown groups, with the greatest increase observed in the GPX4-knockdown group compared with the SOD1-knockdown group. Further studies have revealed delayed wound recovery in GPX4 siRNA-treated cells 2 days after wounding. However, α-tocopherol alleviated the delay in wound healing caused by GPX4 deficiency. This study demonstrated that a decrease in GPX4 levels in HCECs resulted in elevated levels of lipid peroxidation markers such as LDH and 4-hydroxynonenal (4-HNE), which triggered ferroptosis (150). In addition, the application of the ferroptosis inhibitor Fer-1 could mitigate the decreased cell viability and elevated LDH associated with GPX4 gene knockout (152). Research has indicated that compared with their wild-type counterparts, mice with partial GPX4 deficiency exhibit delayed corneal wound healing after epithelial damage, highlighting the critical role of GPX4 in postinjury tissue repair. These findings suggest that blocking ferroptosis may shield corneal epithelial cells from oxidative stress-related cell death when GPX4 activity is compromised (153).

Corneal alkali burns have severe clinical manifestations because of their ability to dissolve proteins. Such injuries frequently result in corneal scarring, neovascular growth, and, in advanced cases, loss of vision (153). Existing therapies are limited in terms of efficacy and possible adverse reactions (154, 155). Alkali burns elevate ROS levels, upregulating the expression of genes such as Nox2, Nox4, vascular endothelial growth factor (VEGF), and matrix metalloproteinase (MMP). This upregulation exacerbates corneal injury by triggering an inflammatory response and driving the growth of new blood vessels, resulting in significant corneal damage through neovascularization (155, 156). New blood vessel growth may cause lipid leakage and buildup in the cornea (157). Elevated ROS levels induce lipid peroxidation, resulting in ferroptosis (158). Research has indicated that the ferroptosis inhibitor Fer-1 shows promise for treating conditions linked to ferroptosis. In a murine model of alkali-induced corneal injury, Fer-1 administration significantly reduced corneal opacity and abnormal neovascularization. These findings strongly implicate ferroptosis as a key mechanism in alkali burn-related corneal damage. Further studies have shown that alkali burns activate ferroptosis and that ROS attack mitochondria and jointly promote the development of ferroptosis in corneal tissue. The efficacy of Fer-1 treatment suggests that ferroptosis may be a potential target for the treatment of corneal alkali burns. Furthermore, compared with free Fer-1, Fer-1 encapsulated in liposomes was more effective at healing corneal alkali burns, and safety assessments conducted both *in vitro* and *in vivo* revealed no significant adverse effects from Fer-1 liposomes (159, 160).

Corneal injury caused by inflammation, trauma, surgery, or infection also results in the excessive production of ROS and reactive nitrogen species (RNS) (161). These compounds stimulate iron ion release and lipid oxidation, inducing ferroptosis (162). Corneal conjunctival lesions and corneal ulcers can occur in severe cases of DED, and recent studies have shown that AKR1C1 safeguards corneal epithelial cells against damage caused by oxidative stress in DED (163). Excessive oxidative stress triggers ferroptosis, which contributes to DED progression in a mouse *in vivo* DED model and an immortalized HCEC line. In addition, Nrf2 can trigger AKR1C1 expression to attenuate iron toxicity-induced cell injury and inflammation in HCECs.

In summary, ferroptosis is key to keratocyte damage. Thoroughly examining how central players contribute to ferroptosis and keratocyte damage and designing potent ferroptosis inhibitors could open the way for novel treatments for corneal injury.

### 4.2 Ferroptosis in retinal pigment epithelial cells

The RPE serves as a cornerstone of retinal structure and function, playing an indispensable part in vision. On its external side, it borders Bruch’s membrane and the choroid, whereas internally, it makes direct contact with PR outer segments. The basal surface exhibits intricate infoldings that dramatically boost cellular surface area, paving the way for streamlined material transport. Acting as the linchpin of the outer blood–retinal barrier, RPECs establish tight junctions that deliver glucose, oxygen, and vital nutrients to PRs while simultaneously clearing away their metabolic waste, keeping PRs’ visual capabilities optimal. Therefore, when ferroptosis strikes RPECs, it can wreak havoc on PRs, causing damage or even their demise, which ultimately sets the stage for the development and advancement of vision-impairing disorders. PRs detect light through specialized organelles called outer segments, where photopigments embedded in membranous discs absorb photons and initiate phototransduction (164). Blinding eye diseases such as RP and DR arise when PRs degenerate. One contributing mechanism is the ferroptosis of RPECs (151). Essential to the function of the blood–retinal barrier, RPECs play a pivotal part in sustaining PR by delivering glucose, oxygen, and a plethora of nutrients, all while removing metabolic byproducts from PRs. This dynamic exchange is paramount for preserving the visual acuity of PRs. Consequently, the onset of ferroptosis within RPECs can trigger PR damage, potentially culminating in their death. This, in turn, is a major factor in the onset and escalation of blinding eye disorders (165).

PR outer-segment membranes are highly enriched in PUFAs, particularly DHA. These PUFAs are susceptible to lipid peroxidation when excessive light exposure or mitochondrial dysfunction generates ROS, thereby amplifying oxidative stress and predisposing photoreceptors to ferroptosis. In the same manner as ROS cause PR damage, iron overload can cause lipid peroxidation and oxidative stress, leading to ferroptosis and subsequent PR damage (166). PRs are exceptionally prone to oxidative damage because of their susceptibility to lipid peroxidation, which can severely compromise their functionality. Antioxidants such as vitamin E and the enzyme GPX4 play crucial roles in shielding these cells by mitigating lipid peroxidation and bolstering their resistance to oxidative stress (167). However, the efficiency of the retinal redox system decreases significantly with age, increasing vulnerability to oxidative damage over time. Current key routes linked to RPEC ferroptosis include amino acid uptake, lipid metabolism, iron regulation, and inflammatory signaling pathways.

System Xc^−^, GPX4, and GSH are canonical factors involved in ferroptosis. In RPECs, GPX4 inhibitors such as 1s and 3R-RSL3 (RSL3) increase lysosome-associated membrane protein 2 (LAMP2) levels, leading to ferroptosis. Supplementation with cysteine and glutamine can restore GSH function, thereby inhibiting ROS-induced death in LAMP2-knockout RPECs (168). Elevated oxygen consumption, prolonged exposure to light, and excessive intake of PUFAs and photosensitizers may cause retinal ROS buildup and induce oxidative stress. Additionally, the excessive metabolism of iron can accelerate this process, ultimately leading to ferroptosis in RPECs (169).

HFE/Fe^3+^–transferrin (TF)–transferrin receptor (TFR) uptake occurs in the basolateral membrane of the RPE, and the distribution and levels of HFE and TFR are essential for iron balance in RPECs. A lack of HFE can lead to iron overload (170). Gnana-Prakasam and colleagues reported that RPECs with suppressed HFE presented traits akin to those of tumor cells, including diminished cell aging, improved motility, and increased glucose consumption (171). RPEC hypertrophy occurs in mice with iron overload, resulting in impaired RPE function.

The protein divalent metal transporter 1 (DMT1) is an endosomal membrane transporter for protons and Fe^2+^ (172) and is present in RPECs. Recent research has indicated that the elevated expression of the iron-regulatory protein IRP1 increases DMT1 levels and simultaneously reduces ferroportin-1 (FPN1) levels. This downregulation of FPN1 can lead to iron accumulation (173). Eventually, FPN1 downregulation promotes the accumulation of Fe^2+^ and induces ferroptosis in RPECs (166). In addition, DMT1 polymorphisms may serve as environmental risk markers for AMD (174).

RPE injury and inflammatory damage are associated with RPEC ferroptosis, and numerous studies have shown that ROS can cause retinal cell damage, leading to retinal disease. ROS can stimulate the generation of LOX metabolites, which then interact with ROS to trigger lipid peroxidation—a key driver of ferroptosis. Research has shown that NaIO_3_-triggered damage to RPECs, along with associated inflammation, occurs through ferroptotic pathways. Notably, the 5-LOX inhibitor zileuton has been shown to mitigate retinal damage and RPEC degeneration caused by NaIO_3_ by suppressing ferroptotic mechanisms (175).

Oxidative stress and free radical-induced damage, particularly in RPECs, drive the progression of AMD. Recent studies have shown that elevated iron levels in the RPE and Bruch’s membrane correlate with advanced AMD stages (176). The retina and RPE are rich in GSH, a vital component for safeguarding RPECs (176). GSH depletion causes cellular death. Research has shown that GSH depletion induces RPEC death via ferroptosis and autophagic mechanisms within a typical *in vitro* setup for AMD. In addition, the results revealed that autophagy contributes to ferroptosis triggered by GSH depletion (177). Totsuka et al. demonstrated that ferroptosis contributes to the death of RPECs (178). Through both *in vivo* and *in vitro* studies, Wei et al. highlighted the significance of ferroptosis in AMD (179). Animal studies have demonstrated that the NaIO_3_-induced AMD model has ferroptosis-related characteristics. This effect can be mitigated by the application of ferroptosis antagonists. Nutritional supplementation with antioxidants may help mitigate the progression of AMD. This safeguarding effect is believed to correlate with the ability of these nutrients to reduce the sensitivity of RPECs to ferroptosis and protect retinal neurons.

In high-glucose environments, the retina, a tissue with significant oxygen demand, is especially susceptible to injury. Nrf2 plays a vital role in antioxidant defense, with studies showing that its activation safeguards retinal tissue in DR models (180). Many studies in diabetic models have demonstrated that increased Nrf2 levels shield these organs from damage by inhibiting ferroptosis. Recent studies have identified initial indicators of nerve cell death and astrocyte activation in DR. Neuronal apoptosis and degeneration are associated and marked by irregular tau protein phosphorylation, a feature that is also present in Alzheimer’s disease. Research has indicated that increased tau expression and phosphorylation may induce ferroptosis in neural tissue. In addition, research has indicated that reducing GPX4 leads to decreases in the numbers of hippocampal neurons and astrocytes in adult mouse models of Alzheimer’s disease. This cell death mechanism aligns with the characteristics of ferroptosis (181, 182).

### 4.3 Ferroptosis in retinal ganglion cells

RGCs constitute neuronal populations situated in the inner retina that are responsible for converting light signals into neural pulses and transmitting them to the visual center of the brain (183). RGCs are neuroretinal elements that link visual sensors to the cerebral cortex to create a visual network. Several visual system disorders cause functional and structural alterations in RGCs (i.e., DR, glaucoma, demyelinating optic neuritis, and ischemic optic neuritis) (184). Ferroptosis contributes to the progression of these illnesses by compromising retinal ganglion cell functionality.

In addition, ferroptosis can induce RIRI and glaucoma by affecting RGCs. The mechanism underlying RIRI involves an initial phase of acute hypoxia (ischemia) caused by blood flow interruption, followed by a second phase of reperfusion injury when blood flow is restored, which exacerbates tissue damage. This damage leads to retinal structural alterations, RGC degeneration, and eventual functional impairment. Reperfusion leads to an overload of ROS and inflammatory cytokines, as well as RGC apoptosis. Glaucoma involves progressive optic nerve damage, leading to distinct structural alterations in the optic disc and retinal nerve fibers, accompanied by progressive RGC death, which will be specifically discussed later.

### 4.4 Ferroptosis in macrophages

Macrophages are white blood cells located within tissues and are derived from precursor cells in the bone marrow. Their main function is to phagocytose and digest cellular debris and pathogens in vertebrates, as well as to activate lymphocytes or other immune cells to combat pathogens. Macrophages are widely distributed in various tissues and have multiple functions, such as secreting cytokines and producing ROS. They mediate inflammatory responses; modulate iron, lipid, and amino acid metabolic processes; and significantly contribute to tissue equilibrium (185). Macrophages stimulated by different microenvironments differentiate into different subtypes, the most common of which are M1 and M2. M1 macrophages amplify inflammation, eliminate pathogens, and inflict tissue damage by secreting a repertoire of proinflammatory mediators, whereas M2 macrophages are involved mainly in tissue repair (fibrosis and neovascularization) (186). Increasing evidence suggests a strong connection between macrophages and ferroptosis.

Macrophages can clear cells that undergo ferroptosis. HMGB1 released from cells undergoing ferroptosis binds to the advanced glycation end-product receptor (AGER) on macrophages and mediates the inflammatory response in macrophages. Several molecules, such as monocyte chemoattractant protein-1 (CCL2) and macrophage inflammatory protein-1α (CCL7), can initiate macrophage recruitment and chemotaxis to increase the magnitude of immune responses. Moreover, macrophage Toll-like receptor 2 (TLR2) identifies and interacts with SAPE-OOH at the membrane of cells undergoing ferroptosis, enhancing phagocytosis and helping to clear ferroptotic cells (187). Macrophages play a key role in controlling iron balance from two main sources. First, macrophages produce iron by engulfing senescent erythrocytes and are a major source of available iron in the body. Second, extracellular iron (Fe^3+^) binds to TF and can enter macrophages via TFR1. Normally, the body can maintain the stability of iron content (188).

When this stabilization is broken, abnormal iron metabolism may oversupply the active form of iron, inducing ferroptosis. Moreover, cytokines secreted by macrophages modulate the activity of intracellular LOX, thereby inducing ferroptosis. Studies have shown that ferroptosis induces iron overload in macrophages, drives M1 polarization, increases inflammatory cytokine release, and impairs tissue repair and immune modulation (189). M1 and M2 macrophages differ in terms of both iron metabolism and ferroptosis susceptibility. M1 macrophages exhibit reduced susceptibility to RSL3-triggered ferroptosis because of the elevated levels of nitric oxide radicals, which inhibit lipid peroxidation.

Some investigators have developed a non-pharmacological biohybrid approach to target ferritin for synergistic ferroptotic immunotherapy by utilizing M1 macrophage microvesicles combined with HKN15-modified Prussian blue nanoparticles (190). In contrast, M2 macrophages present decreased iNOS expression and heightened susceptibility to RSL3-induced ferroptosis. Inflammatory macrophages upregulate SLC7A11 and increase cystine uptake and GSH synthesis via the NF-κB pathway, thereby maintaining intracellular redox homeostasis and resisting ferroptosis (191). According to previous studies (192), when M2 macrophages infiltrate the tumor microenvironment, they can suppress ferroptosis in neighboring cancer cells through paracrine signaling, despite being intrinsically sensitive to ferroptosis.

As people age, the number of macrophages in normal eyes gradually increases. Macrophages contribute to the production of VEGF, which can induce angiogenesis and provide routes of nutritional supply and metastasis for tumors. Because ocular melanoma is located in the eye, which lacks a lymphatic system, tumor cells must escape into the bloodstream to metastasize to other organs, such as the liver. In this process, macrophages facilitate tumor cell escape and metastasis by stimulating angiogenesis. Thus, macrophages play a role as disruptors in ocular melanoma. Experimental evidence has shown that excessive iron accumulation prompts macrophages to shift toward the M1 phenotype, triggering a cascade of inflammatory mediators. This inflammatory response affects both ocular surface structures and lacrimal gland tissue, ultimately fueling the advancement of dry eye disease pathology (193). Liposomal clodronate attenuates iron overload-induced DED by reducing the number of macrophages, particularly M1 macrophages, regulating macrophage polarization, and suppressing inflammatory responses.

### 4.5 Ferroptosis in natural killer cells

After T cells and B cells, natural killer (NK) cells are the third most prevalent type of lymphocyte. They are associated with antitumor activity, antiviral defense, and immune modulation, and also contribute to hypersensitivity and autoimmune disorders. NK cells originate from hematopoietic stem cells in the bone marrow and are among the core cells of the innate immune system, accounting for approximately 5%–15% of all immune cells in the blood. The majority of NK cells exhibit antitumor cytotoxicity. They produce lytic granules comprising a large number of molecules (perforin, granzyme, and human granulysin) that induce cell death in stressed cells. Macrophages can also be called by NK cells to address pathogens and damaged cells in the body, as well as mediate antibody-dependent cellular cytotoxicity (ADCC) (194). This is also an important mechanism through which common antibody drugs exert clinical effects. Owing to the broad anticancer activity of NK cells, the range of NK-cell types used for cancer treatment has become increasingly diverse in recent years (195, 196). One study showed that immune cells, mainly NK cells, eliminate senescent cells and increase animal lifespan by 20%–30% (197).

Recent research has indicated that NK cells associated with tumors exhibit characteristics of ferroptosis, including lipid peroxidation and oxidative stress. These features are associated with the inhibition of NK-cell metabolic activity in the tumor microenvironment, resulting in NK-cell impairment. In contrast, Nrf2 activation modulates the expression of multiple antioxidant molecules, thereby rescuing this dysfunction (198). In tumor-bearing mice, liproxstatin-1 enhances NK-cell viability and antitumor activity by pharmacologically inhibiting ferroptosis. These findings indicate that blocking ferroptosis improves NK-cell survival and antitumor function, suggesting that ferroptosis limits the longevity of NK cells in the tumor microenvironment (199). In ophthalmology, whether the induction of ferroptosis could be exploited as a novel therapeutic strategy against the orbital involvement of highly aggressive nasal-type extranodal NK/T-cell lymphoma remains to be investigated.

neovascular age-related macular degeneration (nvAMD) features choroidal neovascularization (CNV) development, which affects 10% of AMD patients and is the predominant factor leading to vision impairment in AMD patients (200). These irregular blood vessels penetrate the subretinal area, leading to leakage, swelling, and bleeding, which in turn cause a rapid decrease in central vision. NK cells accumulate in the diseased choroid and inhibit pathological changes in nvAMD by promoting NETosis and NET production by neutrophils (201, 202). Rituximab clears B cells through an NK-mediated lytic pathway and is indicated for B cell-driven immunoreactive thyroid eye disease (203, 204).

### 4.6 Ferroptosis in T cells

T lymphocytes arise from lymphoid progenitors in the bone marrow and subsequently differentiate, develop, and mature in the thymus. After receiving orderly and standardized “training”, T cells enter the blood and migrate to lymphoid tissues in the periphery. After receiving antigen stimulation, mature naive T cells develop into effector or memory cells and contribute to adaptive immune responses (205). T cells consist of two main functionally separate types: CD4^+^ helper T cells (Th cells) and CD8^+^ cytotoxic T lymphocytes (CTLs). CTLs, which are characterized by the surface expression of CD8^+^, eliminate infected cells and are commonly referred to as “killer T cells”. Th cells express CD4 as a surface marker and orchestrate adaptive immune responses by secreting cytokines and providing costimulatory signals (206).

In response to immunotherapy, CD8^2^ T cells release IFN-γ, which targets system Xc^−^, curtails cystine uptake, and thereby sensitizes tumor cells to ferroptosis (207). Some researchers have reported that PCIF1 diminishes ferroptosis in CD8^+^ T cells predominantly through the upregulation of genes that regulate ferroptosis, including FTH1 and SLC3A2 (208). GPX4 is essential for protecting T cells from lipid peroxidation and ferroptosis. Notably, while T-cell growth *in vitro* requires system Xc^−^, system Xc^−^ does not appear to be necessary for T-cell proliferation or primary and memory immune responses to tumors *in vivo* (209). Selenium supplementation increases GPX4 expression in follicular helper T cells (TFH cells) and decreases ferroptosis susceptibility, thus enhancing antibody responses in mice vaccinated against influenza. In accordance with these findings, mice with GPX4 deficiency specifically in T cells were unable to fight acute lymphoblastic cerebrospinal meningitis virus or *Leishmania* infection. This immunodeficiency could be avoided through the administration of high-dose supplements with the fat-soluble antioxidant vitamin E, which prevented ferroptosis in GPX4−/− T cells.

The eyes produce suppressed immune responses to avoid inflammation that can hinder vision. It is commonly believed that there are no T cells in the cornea; however, long-lived memory T cells reside in the cornea and can “patrol” and fight viral infections. In an experiment on corneal herpes simplex virus (HSV) infection, a team used multiphoton microscopy to obtain real-time images of living, intact biological tissue to study keratocytes in mice infected with HSV. Images revealed that long-term surviving memory T cells were generated in the eyes of the mice to fight infection. After the virus was cleared, memory T cells remained in the cornea to prevent future reinfection (210). Recent studies have provided evidence that DED is an autoimmune disorder driven by T cells (211). The retinal expression of the glycolysis-related gene LDHA markedly increased in mice with experimental autoimmune uveitis and promoted the migration of effector T cells (Teff cells). The results of these experiments revealed that LDHA inhibition could inhibit the migration of CXCR4-positive pathogenic T cells into retinal tissue to prevent the development of uveitis. Importantly, in Vogt–Koyanagi–Harada disease (VKH), the upregulation of LDHA is increased in CD4-positive T cells, and the inhibition of LDHA reduces the proliferation of CD4-positive T cells to prevent diseases (212).

## 5 Links between ferroptosis and ophthalmology

Ferroptosis, a lipid peroxidation-mediated, iron-dependent form of programmed cell death, has swiftly become a leading focus in ophthalmic disease research in just a few years, as it plays important roles in cataracts, glaucoma, DED, corneal injury, and thyroid-associated ophthalmopathy (TAO), among others. Ferroptosis provides not only a unified explanation for the common terminal damage associated with a variety of eye diseases but also a new window for personalized, mechanism-oriented treatment. By delving into the regulatory processes of ferroptosis—such as iron ion metabolism, ROS generation, and the equilibrium of antioxidant defenses—we can gain clearer insights into its involvement in eye diseases.

### 5.1 Ferroptosis in AMD

The macula, which is located at the core of the retina, is essential for focused sight (213). AMD is a multifaceted age-related eye condition marked by a decline in the architecture and functionality of the macula (214). This disease is the leading cause of blindness in individuals aged 50+ (215). The worldwide incidence of AMD is anticipated to increase from 196 million cases in 2020 to 288 million cases by 2040 (216). In addition, as the global population ages, the societal and economic impact of age-related macular degeneration is poised to increase.

Early macular degeneration is characterized by macular cysts and pigment changes. Advanced AMD is categorized into dry and neovascular (nvAMD) forms (217). Dry AMD features permanent damage to the RPE and PRs, leading to the atrophy of retinal tissue comprising supportive cells under PRs. The degeneration or dysregulation of retinal tissue is a pathogenic marker of AMD. nvAMD, also known as exudative AMD, is characterized by abnormal neovascularization, including CNV and retinal hemangiomatous hyperplasia (RAP) (218, 219), which can lead to macular leakage.

**Figure 3:**
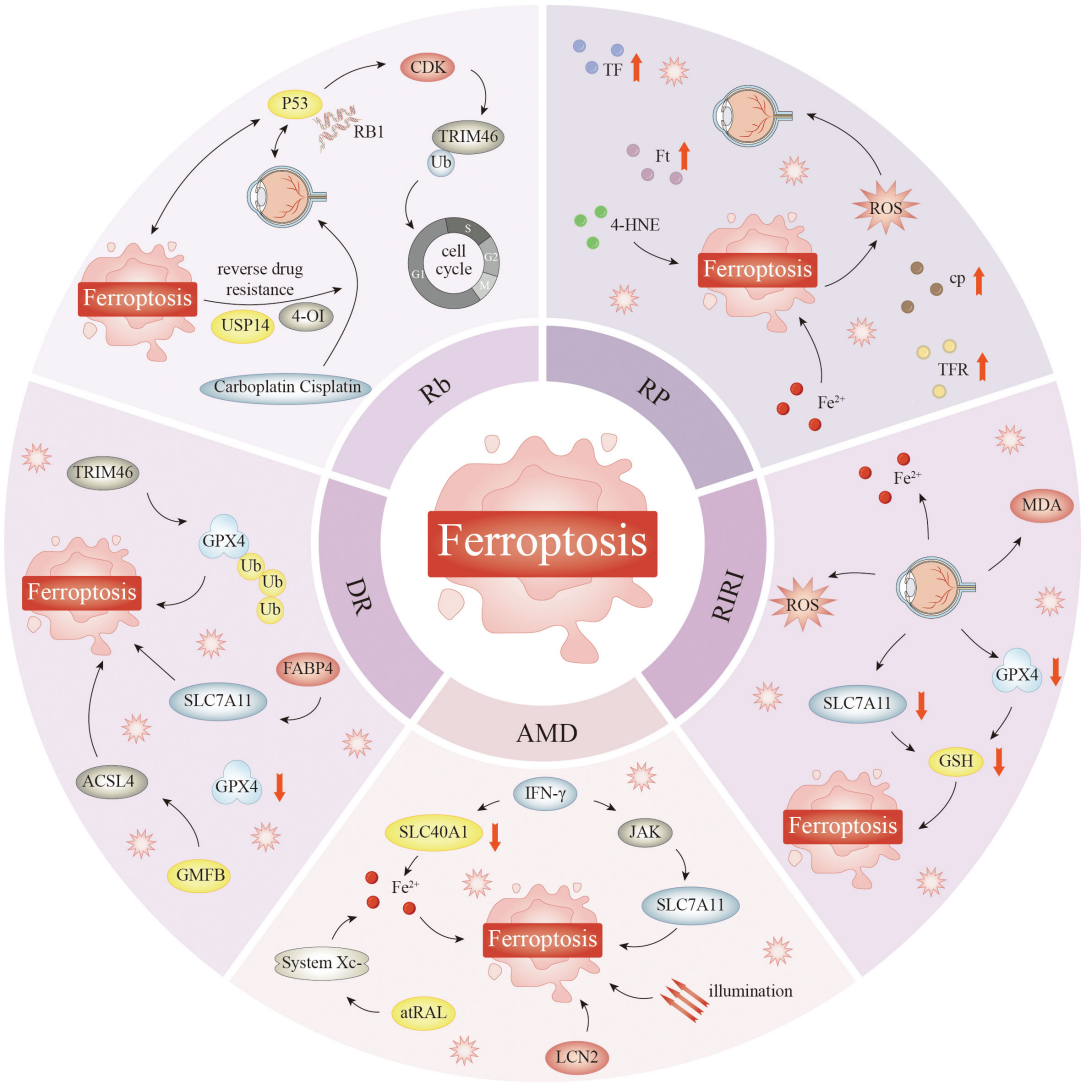
Ferroptosis mediates diseases such as AMD, DR, Rb, RP, and RIRI. Iron overload-induced oxidative stress triggers ferroptosis in RPECs, causing photoreceptor degeneration, a central pathway in dry AMD. Elevated LCN2 exacerbates this iron-dependent cell death by impairing iron export. In patients with AMD, excess iron accumulates in the macula, RPE, Bruch’s membrane, and drusen, while aging retinas show increased lipofuscin, 4-HNE, and MDA, lipid peroxidation markers that mirror the biochemical signature of ferroptosis. IFN-γ can also trigger ferroptosis of RPECs and promote AMD progression. Ferroptosis plays a key role in DR progression, and it promotes disease development by impairing the integrity of capillary endothelial cells. In DR, ferroptosis fuels disease progression by impairing capillary endothelial function. Under high-glucose conditions, TRIM46 is upregulated, triggering ferroptosis that destabilizes the iBRB and damages RPECs, thereby accelerating DR. Ischemia–reperfusion injury significantly downregulates SLC7A11 and GPX4 expression, leading to GSH depletion, which is the core antioxidant axis regulating ferroptosis, and dysfunction is an important mechanism of cell death in ischemia–reperfusion injury. During ischemia–reperfusion injury, ferroptosis occurs in almost all types of retinal cells. RIRI causes retinal accumulation of Fe^2+^, ROS, and MDA. Various studies have demonstrated that ferroptosis is a key factor leading to RP. Experimental models have established ferroptosis as a critical driver of RP. Overexpression or intraperitoneal delivery of human transferrin effectively prevents photoreceptor cell death, and iron chelators likewise rescue retinal degeneration. HO-1 is a key regulator of this process; its inhibition reduces free-iron accumulation, thereby protecting both retinal pigment epithelial cells and photoreceptors from ferroptosis-mediated degeneration. Mutations in the p53 gene and deletion of the RB1 gene have been identified to play an important role in Rb pathogenesis. Both genes have also been shown to be involved in the process of ferroptosis. 4-OI-induced ferritin degradation or USP14-targeted enhancement of ferroptosis efficiently eliminates multidrug-resistant Rb cells, offering a novel strategy to overcome chemoresistance in Rb. Abbreviations: CDK, cyclin-dependent kinase; P53, tumor protein p53; TRIM46, tripartite motif-containing protein 46; Ub, ubiquitin; USP14, ubiquitin-specific protease 14; FABP4, fatty acid-binding protein 4; AMD, age-related macular degeneration; SLC40A1, solute carrier family 40 member 1; GMFB, glia maturation factor beta; atRAL, all-*trans* retinol; DR, diabetic retinopathy; Rb, retinoblastoma; RP, retinitis pigmentosa; RIRI, retinal ischemia–reperfusion injury; RPECs, retinal pigment epithelial cells; 4-HNE, 4-hydroxynonenal; MDA, malondialdehyde; iBRB, inner blood–retinal barrier; GPX4, glutathione peroxidase 4; ROS, reactive oxygen species.

The progression of AMD is associated with oxidative stress, as well as genetic, environmental, and aging-related influences. High levels of PUFAs are located in the outer photoreceptor segment and produce substantial amounts of intracellular ROS, making the retina especially susceptible to damage from oxidative stress (166) (**Figure 3**). Oxidative stress can lead to RPEC loss, causing photoreceptor degeneration in AMD (220); this degeneration is also a causative factor of dry AMD. Recent research has highlighted the pivotal involvement of ferroptosis in the pathophysiology of AMD, notably through the Xc^−^ and GPX4 pathways, and mitochondrial metabolism (221, 222).

The intraperitoneal administration of an SLC7A11 inhibitor in a laser-generated CNV model increased lipid peroxidation and triggered ferroptosis, leading to the expansion of the CNV area in ARPE-19 cells. In contrast, Fer-1 and Lip-1 rescued RPE ferroptosis and VEGF production (223). One study revealed that mice with system Xc^−^ knockdown showed rapidly advancing retinal aging, increased retinal strain, elevated ROS levels, and compromised mitochondrial activity, providing a beneficial framework for investigating retinal aging and AMD (224). Iron, a key producer of free radicals, is closely associated with the pathogenesis of AMD (225, 226). Compared with that in young adults, the retinal iron content in older adults is significantly greater, as iron tends to accumulate in the retina as people age (225, 227). In the retinas of patients with AMD, excessive iron accumulates within the macula, RPE, Bruch’s membrane, drusen, and other structures. Aging retinas show elevated levels of lipid peroxidation-derived products (e.g., lipofuscin, 4-HNE, and MDA) that are consistent with but not exclusive to ferroptosis, which is associated with inflammation and features of AMD (228, 229). The accumulation of iron is a major cause of excess free radical generation in the RPE and is strongly linked to the development of AMD (22). Iron levels in the RPE are markedly elevated in patients with dry AMD (225). In addition, iron-binding agents such as deferiprone (DFP), DFO, and deferasirox (DFX) prevent the Fenton reaction by binding to surplus iron ions, and research has indicated that these agents serve as safeguards against retinal degeneration in various experimental setups (230, 231). GPX4 maintains redox homeostasis and protects RPECs, photoreceptors, and RGCs from glutamate-induced cytotoxicity (232). In multiple oxidative stress models of retinal degeneration, elevated GPX4 expression has been shown to protect photoreceptors and RPECs, suggesting that increasing GPX4 expression may represent a potential gene therapy approach for patients with AMD (47, 48).

Researchers have reported that mitochondrial destruction, fragmentation, and destruction occur in the RPE of patients with AMD (233). Numerous reports have shown that the degree of mtDNA damage and the number of breaks in RPECs are positively correlated with the severity of AMD (234, 235). mtDNA damage can induce ferroptosis and exacerbate both RPE degeneration and the progression of AMD (236). NAIO_3_, H_2_O_2_, and UV light treatment can induce a decrease in the mitochondrial membrane potential and an increase in ROS levels, leading to ferroptosis (237, 238). Ferroptosis in RPECs can be inhibited by the use of mitochondrial ROS quenchers, which can prevent the accumulation of mitochondrial ROS and lipid oxidation. Mitochondrial ROS play a crucial role in ferroptosis.

Chronic inflammation associated with aging promotes the onset and progression of AMD (239). Research has indicated a positive correlation between inflammatory responses and iron buildup. In addition, multiple inflammatory signaling cascades, including the JAK–STAT pathway, participate in ferroptosis–inflammation interactions (61). Proinflammatory factors such as IFN-γ suppress GSH production through the JAK1/2–STAT1–SLC7A11 axis, inducing ferroptosis and RPEC degeneration *in vivo* (179). Interferon-γ (IFN-γ) can also trigger ferroptosis in RPECs and promote the progression of AMD (179). Iron overload can lead to retinal inflammation through the downregulation of cholesterol efflux transporters, leading to increased cholesterol content in the retina (240). Notably, the activation of iron-regulated inflammasomes ultimately leads to oxidative stress and ferroptosis in RPECs (241).

Elevated iron levels increase the expression and activation of complement C3, a key inflammatory regulator (242). *In vivo* studies have indicated that intravenous iron administration resulted in the presence of complement C3 on the basal surface of the RPE, thereby promoting pathological changes (243). Liu and colleagues analyzed a cell model of AMD induced by sodium iodate (SI) and reported that SI can consume intracellular GSH, liberate Fe^2+^ from liposomes, increase cellular ROS levels, and increase lipid accumulation, leading to ferroptosis in ARPE-19 cells (244). Lee et al. reported that SI also stimulates mitochondrial ROS production and promotes ferroptosis in RPECs (175). Tang et al. found that the process of ferroptosis in RPECs triggered by SI is linked to the Nrf2/SLC7A11/HO-1 pathway. Specifically, they reported that higher levels of HO-1 expression correlate with an increase in TFR expression and a decrease in SLC40A1 expression, ultimately leading to iron accumulation (245). Liposomal accumulation of Fe^2+^ may increase HO-1 levels and sustain ferroptosis. Blocking HO-1 or using ZnPP can alleviate the harmful effects on the morphology and function of RPECs and PRs (246).

Ferroptosis in PRs also significantly influences the development of AMD, particularly dry AMD (247). The recycling of 11-*cis*-retinal (atRAL) between photoreceptors and the RPE is essential for maintaining vision (248). ATP-binding cassette transporter A4 (ABCA4) facilitates the movement of atRAL from the photoreceptor outer segment disk to the cytoplasm, whereas RDH8 enzymes are responsible for converting atRAL back into all-*trans* retinol (249). In rodents with genetic deletions involving the Abca4 and Rdh8 genes, photoreceptor outer-segment disc clearance is defective. After light exposure, the PR layer becomes thinner, and A2E accumulates within the retina. Additionally, GSH levels decrease, and the expression of lipid metabolism-related proteins becomes abnormal. These alterations coincide with elevated lipid peroxidation levels (221). Subsequent studies have confirmed that atRAL can trigger ferroptosis in 661W neuronal cell cultures by hindering system Xc^−^, increasing Fe^2+^ levels, and amplifying mitochondrial ROS production. Tang et al. (33) confirmed these findings, as light accelerated PR degeneration via ferroptosis in 661W cells and male Sprague–Dawley rats. Light exposure significantly diminished photoreceptor cell viability and triggered early ferroptosis markers, including iron buildup, mitochondrial shrinkage, reduced GSH levels, elevated MDA levels, and decreased SLC7A11 and GPX4 protein expression.

The transcription factor-driven reprogramming of induced RPE (iRPE) cells has been identified as a robust method to create cells that are resistant to ferroptosis. This finding not only validates the crucial role of phosphoethanolamine/phosphocholine phosphatase 1 (PHOSPHO1) as a master regulator of ferroptosis resistance but also reveals a twofold approach for combating this process. Primarily, PHOSPHO1 curtails the progression of ferroptosis by dampening PE levels within the endoplasmic reticulum (ER), effectively inhibiting lipid peroxidation. Additionally, PHOSPHO1 reduces autophagy and ferritin uptake, consequently decreasing the intracellular accumulation of free iron. *In vivo* rat model experiments verified that PHOSPHO1 effectively protected RPECs from ferroptosis. These findings provide a new theoretical basis and potential target for AMD treatment (250).

ZIP8 functions as a metal ion transporter crucial to the ferroptosis-driven degeneration of RPECs. ZIP8 levels are elevated in patients with AMD, as demonstrated by transcriptomic analysis. The upregulation of ZIP8 expression has also been detected in RPECs and AMD mouse models under conditions of oxidative stress. Notably, this study revealed that ZIP8 knockdown significantly inhibited the oxidative stress response to NaIO_3_ and the development of ferroptosis in RPECs. Specific antibody intervention effectively alleviated the ZIP8-induced obstruction of RPEC degeneration, restored retinal function, and improved visual loss in mice with NaIO_3_-induced AMD. The critical role of ZIP8 in RPEC ferroptosis provides a potential target for the treatment of diseases associated with retinal degeneration, such as AMD (246).

The iron content in the retinal pigment epithelium and Bruch’s membrane is significantly greater in patients with AMD than in people of the same age without AMD. However, the iron concentration in the anterior chamber fluid of patients with dry AMD was more than twice as high as the A2E concentration, and exposure to blue light revealed a role of ferroptosis in the degeneration of RPECs. Via transcriptomic sequencing, Liu et al. found that ARPE-19 RPECs showed ferroptosis-related changes after treatment with SI, and these effects could be blocked by ferrostatin-1 and DFO. Tang et al. further conducted high-throughput sequencing on ARPE-19 cells treated with SI and reported that these cells exhibited ferroptosis. They also found that zinc protoporphyrin IX, a selective heme oxygenase-1 inhibitor, could prevent ferroptosis in RPECs, thereby offering a novel therapeutic target for managing dry AMD. Elevated LCN2 in the RPE exacerbates dry AMD by suppressing autophagy, disrupting iron balance, and triggering inflammasome activity, oxidative damage, and ferroptosis in RPECs (241). Additionally, the activation of genes implicated in ferroptosis is significantly upregulated in retinal tissue in spontaneous dry age-related macular degeneration (SD-AMD), suggesting that the disruption of iron homeostasis and ferroptosis could significantly influence disease onset and development (251).

### 5.2 Ferroptosis in DR

DR is a unique microvascular complication of diabetes and is the leading cause of visual impairment among the working-age adult population. Approximately half of all diabetic patients will eventually develop DR (252). The incidence and speed of DR progression correlate with age in diabetic patients. One study revealed that the incidence of DR was one in five individuals in a younger-onset group and one in two individuals in an older-onset group (253). The currently employed treatment predominantly involves panretinal photocoagulation, anti-VEGF treatments, intraocular steroid administration, and vitrectomy procedures.

In the current study, increased iron content, along with higher values of Cp and TF, was observed in the retinas of DR patients (254), and increased vitreous iron levels correlated with the onset of proliferative diabetic retinopathy (PDR) (255). The reasons may be threefold: first, hyperglycemia leads to heme degradation in hemoglobin and myoglobin, resulting in the detachment of iron ions from heme. Another possibility is that retinal or vitreous hemorrhage increases iron accumulation in patients with proliferative DR. Another possibility is that elevated angiotensin II (Ang-II) levels in DR patients could upregulate iron metabolism-related genes, thereby increasing iron uptake. These studies have indicated that iron ions can contribute to the progression of DR. Iron accumulation can induce oxidative damage and neovascularization.

The aging retina becomes particularly susceptible to oxidative stress, largely because age-related changes cause a surge in the generation of ROS and RNS. In DR, capillary occlusion leads to severe hypoxia, activating hypoxia-inducible factor (HIF) and promoting neovascularization (256). Iron is involved in the HIF-mediated activation of angiogenesis-related genes. In addition, HIF plays a critical role in regulating iron metabolism by regulating the expression of iron-related proteins such as DMT1, Fpn, and TFR.

Oxidative stress is closely linked to the development and progression of DR. Recent research has shown that oxidative stress and photoreceptor impairment may occur before the initial vascular pathology in diabetes (257). PRs are rich in DHA, which is composed of long-chain PUFAs. Excess ROS can attack these lipid targets, and oxidative stress may harm the endothelial cells of retinal blood vessels, leading to vascular leakage and vitreous hemorrhage. As a tissue with high oxygen demand, the retina is especially susceptible to damage under high glucose conditions.

The researchers induced diabetes observed in iron deficiency (HFE) knockout mice, a genetic representation of iron overload. Moreover, findings from these studies have indicated that diabetes increased the degree of vascular injury and BRB leakage in HFE knockout mice. Iron overload triggers the G protein-coupled receptor 91 (GPR91) signaling cascade, increasing renin secretion. GPR91 signaling can mediate vascular growth in PDR, and renin can increase Ang-II expression (258). Ang-II activates VEGF receptors to increase vascular permeability and neovascularization (259). Excess iron-induced oxidative stress can further exacerbate diabetic macrovascular issues, such as atherosclerosis and heart disease (260). In DR, these alterations can impair retinal microvascular function.

Recent research has highlighted ferroptosis as pivotal in DR progression by damaging capillary endothelial cell function. TRIM46 gene expression is increased in diabetic patients (261). Under high-glucose conditions, TRIM46 expression increases in human retinal capillary endothelial cells (HRCECs) and promotes the ubiquitination of GPX4, which leads to ferroptosis (262). One study revealed higher levels of biomarkers associated with iron intoxication in a non-proliferative diabetic retinopathy (NPDR) group than in a PDR group. In NPDR patients, the fundus is primarily defined by capillary leakage and bleeding, and the BRB is disrupted (263). The BRB consists of inner (iBRB) and outer (oBRB) components.

Elevated glucose levels trigger ferroptosis in endothelial cells, compromising iBRB stability (264). Elevated glucose levels impair RPEC viability and function, accelerating DR progression. Investigators have shown that the expression of glial maturation factor-beta (GMFB), a neurodegenerative mediator, is elevated in DR. These researchers injected chemically synthesized GMFB into the vitreous bodies of Sprague–Dawley (SD) rats, impairing RPEC function (37). Laboratory tests have revealed that extracellular GMFB can decrease the number of RPECs and their cell–cell connections and increase ACSL4 expression (35). It promotes the generation of cytotoxic lipids in ARPE-19 cells and triggers ferroptosis. RPE ferroptosis disrupts the oBRB, resulting in macular edema, the primary factor contributing to vision loss in DR.

In addition to GMFB expression, fatty acid-binding protein 4 (FABP4) expression is positively correlated with NPDR/PDR grade and can be used as an independent prognostic biomarker (265). FABP4 drives ferroptosis by inhibiting PPARγ, and recent studies have identified early signs of neuronal apoptosis and reactive gliosis in DR (266) (267) (182).

### 5.3 Ferroptosis in retinal ischemia–reperfusion injury

RIRI can lead to permanent vision loss. The mechanism involves an initial phase of acute hypoxia (ischemia) due to disrupted blood circulation, followed by a second phase of reperfusion injury when blood flow is restored, which exacerbates tissue damage (268). RIRI contributes to glaucoma, retinal artery occlusion, and DR pathogenesis (269, 270). This damage leads to retinal structural changes, RGC death, and functional loss. In particular, reperfusion leads to the overproduction of ROS and inflammatory cytokines and the apoptosis of RGCs (271), causing great damage to retinal function (272, 273).

The results of previous studies confirm the involvement of ferroptosis in retinal ischemic injury in the RIRI model mice. In one study, RIRI induced the accumulation of Fe^2+^, ROS, MDA, and GSH; increased transferrin expression; and reduced the levels of ferritin, SLC7A11, and GPX4 in rat retinal tissue (274). Moreover, ischemia–reperfusion injury significantly inhibits SLC7A11 and GPX4 expression, causing GSH depletion. The GPX4 pathway is a central antioxidant axis that regulates ferroptosis, and its dysfunction is an important mechanism of cell death in ischemia–reperfusion injury (275). The application of the iron chelators deferoxamine and baicalin can ameliorate RIRI (276). Another study revealed that ferroptosis occurred across numerous retinal cell types during ischemia–reperfusion damage (277).

Fer-1 markedly decreased RGC death in both living organisms and laboratory cultures, thereby alleviating ischemia–reperfusion-induced retinal structural and functional damage to the retina. In summary, ferroptosis contributes to retinal ischemia–reperfusion injury, and blocking ferroptosis reduces RGC loss and inflammation. Researchers have reported that the number and diversity of retinal cells are decreased following ischemia–reperfusion-induced damage in mice. In contrast, the numbers of all types of immune cells were notably increased. A significant number of blood-dwelling immune cells invade the retinal tissue, causing blood–eye barrier damage (278). These results indicate that bone marrow cells could be key targets in RIRI.

Some researchers have developed supramolecular nanoparticles that combine the phosphodiesterase 4 (PDE4) inhibitor crisaborole and the ferroptosis inhibitor DFO. These nanoparticles can alleviate retinal IR injury by targeting the ferroptosis pathway and oxidative stress. These compounds also suppress the release of inflammatory mediators and signaling proteins while dampening glial cell activation—a hallmark pathological feature of RIRI (279).

In a mouse model of RIRI, researchers reported that Lycium barbarum polysaccharide-based nanoparticles (PLBP), a nanoparticle derived from goji polysaccharides, directly protects RGCs from ferroptosis. This protection is achieved by activating Nrf2 signaling, reducing intracellular ROS levels, and increasing the antioxidant capacity of RGCs, thereby helping to preserve visual function after ischemia–reperfusion injury. Moreover, PLBP mitigated neuroinflammation and offered indirect protection to RGCs by inhibiting microglial phagocytosis and migration and the release of proinflammatory cytokines and by activating the NF-κB pathway. These combined effects lead to notable vision restoration in mice following retinal injury. This recovery is evidenced by enhanced optomotor responses, improved dark/light preference, and stronger pupillary light reflexes (280).

### 5.4 Ferroptosis in retinitis pigmentosa

RP refers to a group of inherited retinal diseases whose main feature is the slow degradation of PR cells, leading to a gradual decline in the patient’s vision. In RP patients, typical characteristics of the fundus are mainly the waxy yellow color of the optic nerve, retinal vascular stenosis, and osteoid pigment dispersion. The main clinical manifestations are night blindness, reduced peripheral and central vision, disturbed color perception, and a sensation of glare. Currently, no treatment exists for this condition, and treatment is focused mainly on delaying disease progression and improving quality of life (265). RP results from the degeneration of rod and cone photoreceptors, with secondary loss of RPE cells.

In two rodent models of RP, iron overload was observed in the retina. Additionally, in rd10 mice, the levels of iron metabolism-related proteins, including TF, TFR, ferritin (Ft), and ceruloplasmin (Cp), were also elevated. Overexpression or intraperitoneal injection of human transferrin effectively inhibited PR cell death. Moreover, iron chelators have been shown to effectively reverse retinal degeneration (281, 282). Moreover, iron-chelating agents have been shown to be successful at reversing retinal atrophy (283, 284). These findings demonstrate that iron causes photoreceptor degeneration through oxidative stress, with cones being more susceptible than rods (285).

In addition, the levels of 4-HNE, which are indicative of lipid oxidation, were notably increased in the retinal tissues of RP model mice (281). Injecting Fe^2+^ into the eyes of mice killed photoreceptor cells and resulted in the production of superoxide free radicals, causing lipid peroxidation. It also increased 4-hydroxynonenal levels and decreased GPX4 levels, which led to ferroptosis. SOD1 is an important component of the antioxidant defense system of the retina. In genetically modified mice, if more SOD1 is present, the retina can be protected from oxidative damage; however, if not enough SOD1 is present, the oxidative damage to the retina is more severe, and the reduction in SOD1 and GPX4 levels accelerates cone cell function loss, as shown in the RP mouse model.

Liu established an SI-induced RP cell model in RPECs. After the ARPE-19 cells were treated with SI, intracellular free iron levels increased 40-fold, and lipid peroxidation increased significantly; however, GPX4 expression did not change, and the expression of GSH/cysteine was downregulated. The incorporation of ferroptosis inhibitors such as DFO mesylate and Fer-1 inhibited SI-induced cell death. Further mechanistic studies have shown that SI can deplete GSH levels, increase ROS levels, promote the release of iron chelated by iron storage proteins, and subsequently promote lipid damage, ultimately leading to ferroptosis in RPECs. The iron chelator DFO retards photoreceptor degeneration in animal models of RP, supporting a role for ferroptosis in RP progression (244). Similarly, Tang and colleagues demonstrated that ferroptosis is a pivotal cause of RP and suggested that HO-1 is a critical modulator of this process. Inhibiting HO-1 expression could prevent the degeneration of RPE and photoreceptor cells by decreasing Fe^2+^ accumulation.

### 5.5 Ferroptosis in retinoblastoma

Rb is the primary intraocular cancer in infants and children and can metastasize and be life-threatening if left untreated. Rb accounts for approximately 2% to 3% of pediatric tumors (286). Annually, there are approximately 8,000 cases worldwide, with approximately one-third of affected children having Rb that affects both eyes. A white pupil is the most common first symptom of Rb, and other complications include strabismus or more severe symptoms such as red eye pain. The progression of Rb is strongly linked to the mutation of the RB1 gene, the first identified human tumor suppressor gene (287, 288). The disease is characterized into stages A–E. The main clinical treatment is eye-protecting treatment, and the success rate of eye-protecting treatment in stages D and E is significantly reduced. At present, exploring the treatment of this disease is important.

Both p53 gene mutation and RB1 gene deletion are key factors in Rb development and are implicated in triggering ferroptosis. The RB1 gene is considered a key factor in liver cancer, and studies have shown that sorafenib, as the only first-line treatment for advanced liver cancer, can induce ferroptosis in liver cancer. The significant downregulation of the Rb protein increases the vulnerability of hepatocellular carcinoma (HCC) cells to ferroptosis and the efficacy of sorafenib, indicating that the loss of the Rb gene accelerates the progression of ferroptosis (289). These findings suggest that elevated p53 levels cause ferroptosis, whereas p21 can act as an inhibitor of ferroptosis. Furthermore, E2F overexpression inhibited ferroptosis in a p21-dependent manner, which aligns with prior findings that E2F activates p21 transcription.

Carboplatin serves as the primary first-line chemotherapy for treating human Rb; however, acquired multidrug resistance (MDR) has emerged following chronic carboplatin therapy. In these carboplatin-resistant Rb cells, the expression of proteins linked to ferroptosis was elevated, whereas the activity of necroptosis-related genes (e.g., RIPK1 and MLKL) did not significantly change. Multiple ferroptosis inducers efficiently cleared these resistant cells, suggesting that the ferroptosis mechanism could be exploited to overcome the chemoresistance of Rb (290). The investigators assessed the efficacy and safety of 4-Octyl itaconate in inducing ferroptosis in MDR Rb cells. 4-Octyl itaconate (4-OI) is a metabolite capable of inducing ferritin-dependent ferroptosis (291).

It significantly induced ferroptosis in Rb cells and increased intracellular free iron and lipid ROS levels, thereby effectively killing resistant cells. Future studies could investigate whether 4-OI induces ferroptosis in a ferritin-dependent manner in Rb cells. In addition, USP14 is one of three deubiquitinating enzymes (USP14, Uch37, and Rpn11) that bind to the proteasome. It can increase the sensitivity of Rb cells to cisplatin by mediating ferroptosis, which may represent an important target for overcoming resistance to cisplatin in Rb (292).

### 5.6 Ferroptosis and corneal disorders

**Figure 4:**
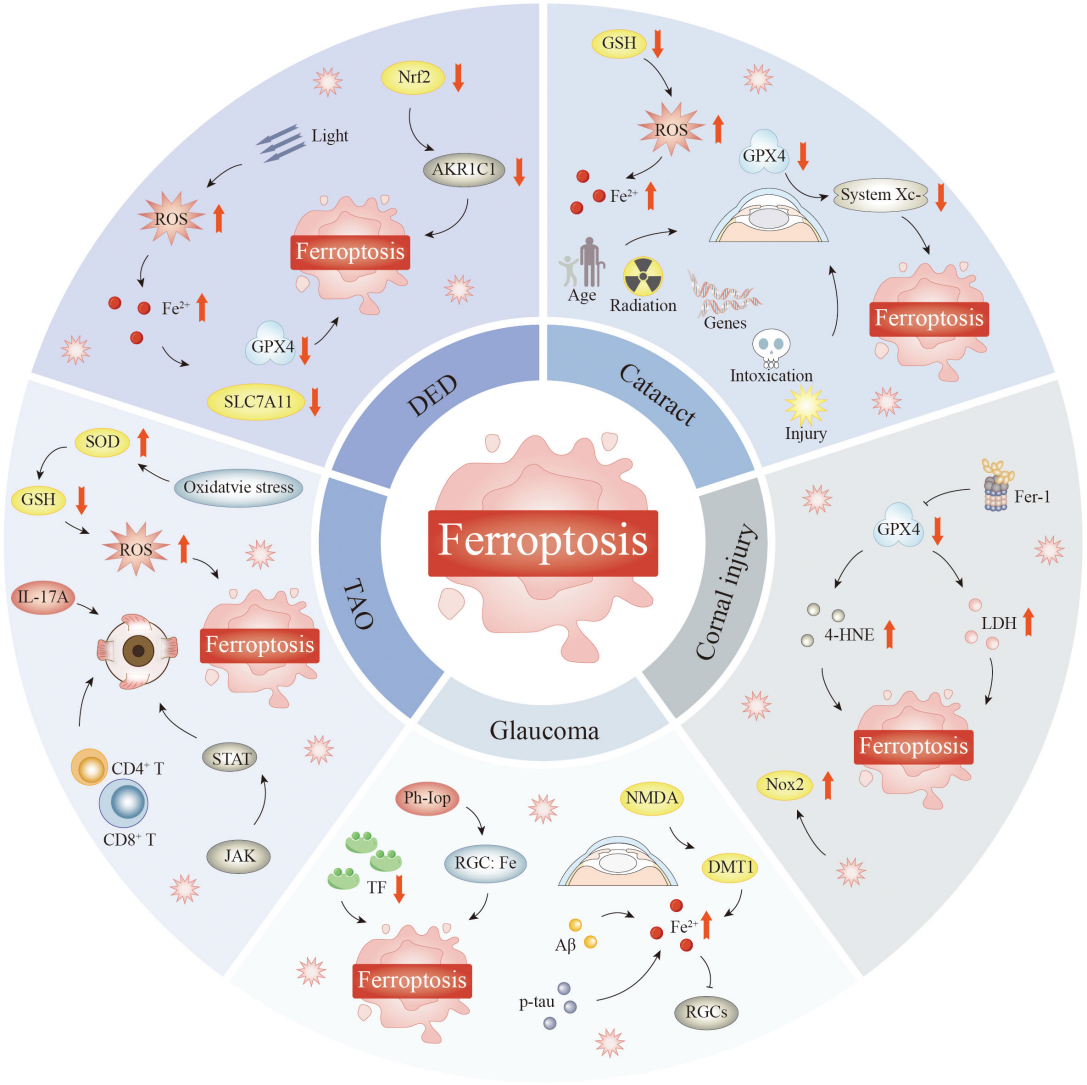
Ferroptosis mediates diseases such as TAO, DED, cataracts, corneal injury, and glaucoma. Inhibition of ferroptosis protects corneal epithelial cells from oxidative stress-induced cell death in the absence of GPX4, which is involved in the course of corneal alkali burns and plays a role in keratitis. Experiments in HCEC revealed that activation of the transcription factor Nrf2 upregulated AKR1C1; when AKR1C1 was knocked down, cell viability was reduced, along with increased lipid peroxidation; excessive oxidative stress-induced ferroptosis was involved in DED pathogenesis. Iron overload was found around retinal epithelial cells, RGCs, and optic nerve axons in glaucoma patients; iron was reduced in cones; TF expression levels were increased. TF could interfere with different cell death mechanisms during the pathogenesis of glaucoma, indicating that iron metabolism would affect glaucomatous neuropathy to some extent. Accumulation of reactive oxygen species and disturbance of redox homeostasis in the lens play critical roles in the development and treatment of diseases. The ability of GSH to synthesize new enzymes decreased, and patients showed impaired lens redox homeostasis, decreased GSH levels, disrupted GPX activity, increased iron redox content, and increased lipid oxidation, which suggests that age-related cataracts are closely related to ferroptosis. ROS generated during the inflammatory response can induce cell damage and play a key role in activating ferroptosis. Markers of oxidative stress are elevated in orbital tissue of TAO patients, suggesting that reactive oxygen species accumulation may trigger orbital fibroblast ferroptosis. Abbreviations: GSH, glutathione; AKR1C1, aldo-keto reductase family 1 member C1; SOD, superoxide dismutase; IL-17A, interleukin-17A; TAO, thyroid-associated orbitopathy; DMT1, divalent metal transporter 1; p-tau, phosphorylated tau protein; RGCs, retinal ganglion cells; DED, dry eye disease; GPX4, glutathione peroxidase 4; HCEC, human corneal epithelial cell; TF, transferrin receptor; ROS, reactive oxygen species.

The inhibition of ferroptosis prevents oxidative stress-induced death of corneal epithelial cells in the absence of GPX4 (153). Ferroptosis contributes to the progression of corneal alkali burns (**Figure 4**). Research has indicated that the specific ferroptosis inhibitor Fer-1 can ameliorate the condition of mice suffering from corneal alkali burns (158). In an alkali burn mouse corneal model, phytic acid alleviated corneal structural damage and promoted corneal function. Researchers have also reported that PA can protect limbal epithelial cells (LECs) from oxidative damage. Moreover, PA can also inhibit the induction of ferroptosis in LECs during oxidative stress *in vitro* (293). Keratoconus (KC) is a prevalent corneal thinning disorder and is a primary reason for transplant surgery worldwide (294, 295), but its underlying mechanisms remain unclear. ACSL4 is the most promising biomarker for keratoconus and is expressed predominantly in corneal stromal cells. Laboratory studies have additionally validated the pivotal involvement of ACSL4 in the progression of KC. ACSL4 plays an important role in the process of ferroptosis, and the results of a previous study provide ideas for the future use of ferroptosis to regulate ACSL4 to treat KC (296).

Ferroptosis also plays an important role in keratitis. Severe eye infection and bacterial keratitis (BK) can lead to pronounced inflammation and corneal damage, resulting in decreased vision (297, 298). One study was performed using a mouse model of keratitis caused by *Pseudomonas aeruginosa*. Mice were infected with *P. aeruginosa* on Day 0 and subsequently treated from Day 1 to Day 7 with either levofloxacin (LEV) alone or LEV in combination with the ferroptosis inhibitor Fer-1. In the BK mouse model, the LEV + Fer-1 group presented lower levels of inflammatory factors, reduced corneal scarring, and lower Fe^3+^ levels. Notably, GPX4 and SLC7A11 levels were elevated in the LEV + Fer-1 group. *In vitro* experiments revealed that Fer-1 treatment effectively reversed the LPS-induced alterations in ROS, Fe^2+^, GPX4, and SLC7A11 levels in corneal stromal stem cells (CSSCs).

These findings indicate that ferroptosis is significantly involved in the pathogenesis of BK and that the inhibition of ferroptosis may help reduce inflammation and diminish corneal opacity, thereby improving BK patient outcomes (299). *Fusarium* is a primary species responsible for fungal keratitis, a prevalent cause of blindness. Oxidative stress is widely recognized as a significant factor in the development of keratitis caused by *Fusarium* species, and some investigators have concluded that ferroptosis can significantly affect the progression of *Fusarium*-induced keratitis through data collection analysis (162).

### 5.7 Ferroptosis and dry eye disease

DED is a multifactorial condition characterized by the loss of tear film homeostasis, which leads to a self-perpetuating cycle of ocular surface inflammation and damage (300, 301). Dysfunction of ocular structures and various systemic diseases, environmental factors, and living habits, such as long-term wearing of contact lenses, can lead to the occurrence and development of DED (302). The risks of DED, including instability, ocular surface damage, nerve paresthesia, and inflammation, have long been overlooked (303).

DED often manifests through telltale signs such as hazy vision, persistent dryness, and heightened sensitivity to light. In extreme cases, patients may develop corneal and conjunctival damage, filamentary keratitis, or even ulceration—complications that can progress to vision loss if left untreated. These debilitating effects not just compromise ocular health but also disrupt everyday activities and professional productivity, underscoring the condition’s far-reaching impact on quality of life (163). DED is also known as incurable terminal disease, for which the standard treatments include artificial tears and medications, although these may not work for all patients. Therefore, exploring the etiology of DED and developing new treatment options are very important.

Some researchers have used subcutaneous injection of scopolamine combined with a dry environment to establish DED mouse models, and immortalized human HCEC-2 cells were cultured under hypertonic conditions to establish hypertonic cell injury models and measure the levels of intracellular Fe^2+^, cellular ROS, and lipid peroxidation. The findings revealed significant changes in the levels of ferroptosis-related markers, intracellular iron, and lipid peroxidation in corneal epithelial cells in a hyperosmotic injury model.

Ferroptosis in hyperosmolarity-induced HCECs was suppressed when excessive oxidative stress was mitigated. In addition, the activation of the transcription factor Nrf2 in HCECs upregulated AKR1C1 under hyperosmotic conditions. When AKR1C1 was knocked down, cell viability was reduced, and lipid peroxidation was increased. Conversely, the overexpression of AKR1C1 had the opposite effect. Corneal defects enhanced inflammatory responses following the inhibition of AKR1C1 in mice. These findings indicate that ferroptosis triggered by high oxidative stress contributes to DED development. Nrf2-mediated expression of the transcription factor AKR1C1 reduced ferroptosis-induced cell injury and attenuated inflammatory responses (304).

Oxidative stress in the tear film and ocular surface increases considerably among DED patients (305). Therefore, ferroptosis can significantly affect DED development. In a previous study, researchers designed adhesive liposomes (Cyclosporine A & Fer-1 loaded adhesive liposomes decorated with chitosan and fibronectin (CF@SNPs)) that codeliver cyclosporine A (CsA) and the ferroptosis inhibitor Fer-1 to achieve synergistic anti-inflammatory and anti-ferroptotic effects on ocular tissues. First, the researchers developed an *in vitro* model simulating DED by exposing HCECs to hydrogen peroxide (H_2_O_2_) and a hypertonic solution (HS) to mimic a dry eye environment. The experimental findings indicated that Fer-1 markedly ameliorated cell damage in the DED model, suggesting that ferroptosis plays a pivotal role in DED and that Fer-1 may inhibit ferroptosis through the p53-SLC7A11 signaling pathway.

In addition, through RNA sequencing analysis, researchers revealed the effects of CF@SNP treatment on the expression of ferroptosis-related genes and inflammation in a mouse model of DED. They reported that CF@SNPs significantly downregulated the expression of genes related to both ferroptosis and inflammation. Immunofluorescence analysis revealed that CF@SNP treatment significantly reduced ROS levels, iron ion levels, and lipid peroxidation while decreasing inflammatory factor expression. These findings confirm the efficacy of CF@SNPs in mitigating the pathology of DED. This study systematically evaluated the potential of CF@SNPs in experiments assessing DED via both *in vitro* and *in vivo* methodologies, establishing a scientific foundation for the development of novel DED therapies (306).

Under high osmotic pressure, the small-molecule compound D609 significantly restored GPX4 expression and reduced intracellular iron ion accumulation. This activity inhibited cellular ferroptosis and protected corneal epithelial cell activity. Through combined transcriptomic and proteomic analysis, they reported that D609 inhibited hyperosmolarity-induced oxidative stress and ferroptosis by increasing melatonin (MT) protein expression and modulating the Nrf2 pathway. PAA-CD-DA increased the ocular bioavailability of D609, enabling targeted DED treatment (307).

### 5.8 Ferroptosis in glaucoma

Glaucoma is the number one cause of permanent vision loss worldwide and is characterized by long-term and persistent deterioration of the optic nerve. This disease causes specific changes in the optic nerve head and retinal nerve fiber layer and is accompanied by the progressive death of RGCs and visual field defects. Intraocular pressure is a key modifiable factor (308). The current primary therapeutic objective for glaucoma is to delay disease progression and maintain visual function and quality of life (309). The selective and irreversible loss of RGCs, the sole central afferent neurons of the retina, is the underlying pathological mechanism in glaucoma (310).

Compared with that in the retinas of healthy individuals, the iron distribution in the retinas of glaucoma patients varies. In glaucoma patients, iron overload is observed around RPECs, RGCs, and optic nerve fibers, whereas iron levels in cone photoreceptors are reduced (311). The levels of the iron-regulating protein TF are elevated in the retinas of monkeys and humans with glaucoma. Elevated TF levels may help protect RGCs from ocular hypertension by regulating iron homeostasis, which in turn can influence various cell death pathways, including apoptosis and ferroptosis. These findings suggest that iron metabolism can affect glaucomatous neuropathy to some extent. A recent study revealed that serum iron levels are higher in patients with acute primary angle-closure glaucoma (PACG) than in healthy people, indicating that iron metabolism may be involved in the regulation of retinal ganglion cell damage in the case of elevated intraocular pressure [pathological intraocular pressure (ph-IOP)] (312).

Ph-IOP is a key glaucoma characteristic and a major factor leading to RGC loss in patients with glaucoma (313). However, the sole management of the IOP does not entirely avert the depletion of RGCs in glaucoma. Yao et al. reported that elevated ph-IOP disrupts retinal iron balance, causing Fe^2+^ overload, particularly in the RGC layer (314). Elevated iron levels impair retinal redox equilibrium and induce RGC ferroptosis.

NCOA4-mediated degradation of FTH1 is important in retinal iron metabolism disorders after ph-IOP injury (314). The inhibition of NCOA4 resulted in decreased degradation of FTH1 and decreased bivalent iron content in the retina. This significantly reduces iron ion levels and alleviates RGC damage (315). When glutamate stimulates *N*-methyl-d-aspartate (NMDA) receptors on the surface, it activates a GTP-binding protein called Dexras1, which in turn stimulates DMT1, allowing cells to absorb more iron (316, 317). Sakamoto et al. reported increased Fe^2+^ levels and apoptosis in retinal ganglion cells following the intravitreal injection of NMDA (318). Iron-binding agents safeguard retinal ganglion cells against NMDA-induced excitotoxicity by decreasing intracellular iron levels and oxidative stress in rodents (318, 319).

In glaucoma, the increased levels of Aβ, p-tau, and amyloid precursor protein initiate an inflammatory response, leading to cytokine secretion and iron overload. Iron activates inflammatory factors via the Fenton reaction (320). Therefore, by further increasing the production and accumulation of Aβ, the redox state of RGCs forms a positive feedback loop with iron ions, exacerbating the loss of RGCs in glaucoma. Iron overload can damage mitochondria. The mitochondria in RGCs from glaucoma subjects and rodent glaucoma models were smaller, rounder, and more fragmented, suggesting a link between glaucoma, iron levels, and mitochondrial dysfunction (321). Mitochondrial impairment is pivotal in the degeneration of RGCs in glaucoma (322). By protecting mitochondrial function, ferroptosis inhibitors can improve the survival of RGCs and help us maintain the structure of the retina (323).

### 5.9 Ferroptosis in cataracts

Cataracts refer to degenerative changes in optical quality resulting from reduced transparency or color changes in the lens. The pathogenesis of cataract is complex. The lens resides in an intraocular fluid environment. Any factor that affects this intraocular environment, such as aging, genetics, metabolic abnormalities, trauma, radiation, poisoning, local nutritional disorders, and certain systemic metabolic or immune diseases, can directly or indirectly cause the lens to become opaque. Epidemiological studies have shown that ultraviolet irradiation, diabetes, hypertension, cardiovascular disease, body trauma, excessive alcohol consumption, and smoking are associated with the formation of cataracts (324). Cataracts account for 50% of global blindness and one-third of all vision impairment cases. As the global population continues to grow older, this disease continues to be a major public health burden, and further research on this disease is needed (325).

Research on age-related cataracts has revealed that the accumulation of reactive oxygen species and the destruction of the redox balance of the lens are core factors that lead to the development of diseases and affect treatment methods. In patients, the ability to synthesize GSH in the lens decreases, and patients exhibit disrupted lens redox balance, reduced GSH concentrations, impaired GPX function, elevated iron redox levels, and increased lipid peroxidation, which indicates that age-related cataracts are closely related to ferroptosis (326, 327). Research by Reddy and his collaborators revealed that mice lacking the GPX1 gene had significantly increased light scattering in the lens nuclei of their eyes (328). The mice developed age-associated cataracts over time. These extensive findings of heightened lipid peroxidation and reduced GPX activity and the accumulation of redox-active iron in aging and cataractous human lenses strongly support the occurrence of ferroptosis during lens aging and cataractogenesis. In both *in vitro* and *ex vivo* models, Wei et al. reported that the system Xc^−^ inhibitor erastin and the GPX4 inhibitor RSL3 increased the susceptibility of lens epithelial cells to ferroptosis (329). Age-related ferroptosis sensitivity increases in both human LECs and mouse lens epithelium.

An important link in ferroptosis is lipid peroxidation, and increasing evidence suggests that lipid peroxidation contributes to cataractogenesis in the early phases. Babizhayev reported the induction of posterior subcapsular cataracts in rabbits via intravitreal phospholipid lipoperoxide application in a model study. In addition, iron overload can induce cataract formation (330). Ocular siderosis, resulting from elevated iron accumulation in eye tissues due to foreign bodies containing iron, can lead to the formation of cataracts (331). TSTA3 is a key enzyme in GDP-fucose synthesis and regulates intracellular fucosylation levels. TSTA3 overexpression or knockdown directly affects the glycosylation status of lens epithelial cells, which in turn alters their function and metabolic pathways. TSTA3-regulated fucosylation may influence ferroptosis development by regulating key nodes of these metabolic pathways, such as GPX4 expression. Researchers have reported that ferroptosis is a significant factor in high myopic cataracts (HMCs). They reported that the overexpression of discoid domain receptor tyrosine kinase 2 (DDR2) activates the Src-Hippo pathway, which in turn downregulates GPX4. This ultimately increases the ferroptosis sensitivity of the lens epithelium in highly myopic eyes, thereby promoting the formation of nuclear cataracts (332). *In vivo*, the injection of RSL3 into the anterior chamber of a mouse model of high myopia induced more severe lens nucleus opacification, which was partially ameliorated by ferroptosis and DDR2 inhibitors. Researchers have revealed that CD82 is strongly associated with dexamethasone (DEX)-triggered ferroptosis in lens epithelial cells. DEX induced ferroptosis via CD82-P53-induced suppression of SLC7A11 and GPX4 expression. The inhibition of iron release by Lip-1 reduces the development of glucocorticoid-induced posterior subcapsular cataracts in a DEX-treated rat model. Glucocorticoid-induced posterior subcapsular cataract (GIC) is strongly linked to ferroptosis enhancement via the CD82-P53-GPX4/SLC7A11 axis (333).

### 5.10 Ferroptosis in TAO

TAO is a condition characterized by an organ-selective autoimmune reaction that affects mainly the orbital muscles and surrounding structures (334). One of these antibodies reacts with thyroid cells and orbital fibroblasts, triggering inflammation in the orbital muscles, connective tissues, adipose deposits, and tear-producing glands. The disease features the infiltration of polymorphous cells with enhanced glycosaminoglycan excretion. TAO is characterized by an increase in orbital contents that can cause the orbit to expand to even eight times the normal size, with a risk of secondary intraocular pressure increase, optic nerve compression, subsequent myofiber degeneration leading to fibrosis, and restraint effects on the affected muscle leading to restrictive myopathy and diplopia (335). TAO is less prevalent in men than in women. In a previous study, most patients in a TAO cohort had mild TAO, whereas 5%–6% of patients had moderate to severe TAO (336).

Selenium is a recognized suppressor of ferroptosis, and selenium supplementation has been shown to improve the quality of life in individuals with mild TAO (337, 338). Teprotumumab, a human anti-IGF1R monoclonal antibody, has been demonstrated to be detrimental to the glycolytic phenotype in experiments with graves’ orbitopathy orbital fibroblasts (GO OFs) from patients with TAO. This leads to ferroptosis in GO OFs (339). This medication was recently approved by the Food and Drug Administration (FDA) for active, moderate-to-severe TAO, suggesting that ferroptosis may contribute to TAO progression and may become a new therapeutic target.

Oxidative stress contributes to ferroptosis and influences TAO pathogenesis. In patients with TAO, the oxidative stress response is characterized by elevated levels of SOD and lipid peroxides, whereas GSH concentrations decrease within the orbital connective tissue (340). In addition, the regulation of the Nrf2/ERK/HO-1 signaling pathway alleviates oxidative stress in orbital fibroblasts (341).

ROS produced during inflammatory reactions can incite cellular harm and significantly trigger ferroptosis. Elevated oxidative stress markers in TAO orbital tissue imply that ROS accumulation may induce ferroptosis in fibroblasts (342). At the onset of TAO, CD4^+^ T cells can activate and multiply orbital fibroblasts and fat cells (343). In addition, B cells, macrophages, and dendritic cells increase tissue swelling and scarring. This dysregulation of immune cells and cytokines establishes a proinflammatory environment that drives the pathogenesis of TAO. Tea-derived polyphenols have been shown to reduce inflammation triggered by lipopolysaccharides through the modulation of the NF-κB/NLR family pyrin domain 3 (NLRP3) pathway (25). In addition, the JAK–STAT axis is crucial for modulating orbital inflammation in TAO (344). Concurrently, it is pivotal in mediating ferroptosis.

Ferroptosis is also important in the fight against fibrosis. For example, triptolide reduces liver fibrosis through the HO-1 signaling pathway (345). However, excessive iron accumulation and ferroptosis in the liver were found to aggravate acetaminophen-induced fibrosis in a mouse model (346). Subsequent experiments revealed that ferroptosis may affect tissue fibrosis by modulating related signaling pathways and increasing interactions between cells. These findings provide a potential new direction for the development of antifibrotic treatments for TAO.

TAO is a complex autoimmune disease that affects structures in the orbit, such as extraocular muscles, orbital fat, and the lacrimal gland. Studies have shown that older TAO patients are more likely to experience posterior thickening of extraocular muscles. This thickening can lead to double vision and limited eye movements (347). It has been shown that cysteine deprivation and treatment with erastin induce ferroptosis and subsequently lead to orbital fibroblast proliferation (348). In TAO patients, increased ferroptosis of orbital adipose tissue is observed. Lacrimal gland tissue plays a vital role in preserving the integrity of the ocular surface. An investigation of the T-cell immunophenotype in the lacrimal glands of TAO patients revealed that one of the hallmarks of lacrimal gland inflammation is the marked infiltration of interleukin-17A (IL-17A)-producing T helper 17 (Th17) cells. IL-17A can promote the differentiation of lacrimal duct fibroblasts into myofibroblasts or adipocytes (349). In addition, a study revealed that corneal nerve damage increased ferroptosis in lacrimal tissue, which resulted in decreased tear production (350). In TAO patients, orbital fat expansion and extraocular muscle hypertrophy together lead to inflammatory infiltration and pyroptosis, and TAO-related lacrimal gland injury contributes to DED. In summary, ferroptosis clearly leads to orbital tissue injury in TAO patients and exacerbates existing orbital lesions among such individuals.

## 6 Targeting ferroptosis to treat eye diseases

### 6.1 AMD

Several inhibitors of ferroptosis exhibit protective effects on retinal neurons and hinder the progression of age-related macular degeneration. Recent research has revealed that the tumor suppressor protein p53 plays a key role in triggering ferroptosis by promoting reactive oxygen species generation while decreasing SLC7A11 expression. A groundbreaking study by Yang and colleagues demonstrated that an extract derived from Fructus Lycii and *Salvia miltiorrhiza* (FSE) effectively prevents photoreceptor cell death through ferroptosis. This protective mechanism occurs via the regulation of the P53/SLC7A11 pathway in 661 W cells exposed to hydrogen peroxide-induced oxidative stress (247). Research has indicated that a higher intake of omega-3 long-chain polyunsaturated fatty acids (*n*−3 LC-PUFAs), a component of fish, may be beneficial for patients with AMD. *n*−3 LC-PUFAs are essential for retinal function, notably contributing to the phototransduction process and resistance to oxidative stress, inflammation, and vascular damage—key contributors to AMD. Animal research in both laboratory and live settings has demonstrated that *n*−3 LC-PUFAs have the potential to maintain retinal integrity in AMD patients (351). LOXs are a family of enzymes that metabolize arachidonic acid and PUFAs, contribute to lipid peroxidation, and induce ferroptosis. Zileuton-mediated inhibition of 5-LOX reduces ROS-induced lipid peroxidation, mitochondrial damage, DNA damage, and iron-related toxicity, as verified by *in vitro* experiments and animal models of dry AMD.

Researchers have confirmed that ferroptosis leads to RPEC loss, disrupts mitochondrial function, and increases lipid peroxidation in ferric ammonium citrate (FAC)-treated ARPE-19 cells and mouse models. The characteristic lesions of dry AMD were highly consistent with the pathological findings of the ferroptosis experiments. These two points highlight the critical effect of ferroptosis on dry AMD, and in this study, *Rhodiola rosea* activated the Nrf2 pathway. The expression of the antioxidant proteins Nrf2, SLC7A11 (cystine transporter), and GPX4 (lipid peroxidase) was upregulated, and iron ion accumulation and ROS generation were inhibited. *R. rosea* also reduced macrophage infiltration and alleviated pathological damage in RPE cells (352). Other researchers have designed melanin-like nanoparticles called Concanavalin A-decorated melanin-like nanoparticles (ConA-MelNPs) as novel ferroptosis inhibitors. ConA-MelNPs chelated iron ions, significantly reduced mitochondrial injury triggered by oxidative stress, and prevented NaIO_3_-induced ferroptosis in RPECs (353).

### 6.2 DR

Almost all patients with diabetes develop DR. Drug therapy, such as anti-VEGF therapy, has led to drug resistance in clinical practice. The treatment of DR with drugs is still under investigation. Ferroptosis plays an important role in the pathogenesis of DR. Targeting ferroptosis to treat DR has become a new research direction.

The two key factors involved in preventing ferroptosis, namely, iron activity and lipid peroxidation, have been shown to be beneficial in DR. DFO significantly contributes to mitigating iron overload conditions. In a murine disease model, vitreous injection of DFO significantly decreased retinal iron content and reduced vascular leakage. However, the ability of DFO to penetrate the BRB is limited (354). Lactoferrin (LF) is an iron-binding glycoprotein derived from mucous cells and neutrophils. LF has a unique ability to break through the BRB (355). LF not only captures reactive iron in the retinal environment and reduces ROS production but also directly removes ROS (356). PMX500FI, an A-lipoic acid derivative studied by Moos et al., can pass through the BRB while also catching iron in the retina. It activates the Nrf2 signaling pathway and inhibits oxidative stress (357, 358). Nrf2 acts as a vital *in vivo* antioxidant protector, with research indicating that Nrf2 pathway activation safeguards retinal tissue in patients with diabetes (359, 360). Similarly, numerous investigations into diabetic cell targets have indicated that increasing Nrf2 levels safeguards against ferroptosis, thereby mitigating organ damage.

The inhibition of lipid peroxidation is another important strategy for preventing ferroptosis. Numerous cellular and animal studies have validated the effectiveness of ELABELA (ELA) and ferrostatin-1 in regulating the iron attenuation process in the retina through the system Xc^−^GPX4 pathway activation (361). The alleviation of ER stress is important for slowing DR progression (362). The alleviation of DR stress may be achieved by ameliorating oxidative stress and inflammation in cells, restoring intracellular iron homeostasis, and attenuating membrane lipid peroxidation. Wang et al. reported that blueberry anthocyanin extract inhibits ER stress, thereby providing relief for DR patients (363). In addition, the compound endoplasmic reticulum was shown to reduce ER stress and decrease inflammatory factor expression. This intervention showed promising efficacy in lowering lipid peroxidation, subsequently halting the intricate interactions among the processes that are associated with oxidative stress, endoplasmic reticulum stress, and inflammation (364).

Among natural products, quercetin inhibited TFR1, upregulated FPN, and decreased free iron levels; 50 μM quercetin reduced the percentage of ferroptotic cells from 42% to 12% in a high glucose-induced Müller cell model. Resveratrol can inhibit ferroptosis through stimulation of the Nrf2/HO-1 pathway and suppression of the p53-SLC7A11 axis. In mice with Streptozotocin (STZ)-induced diabetes, 8 weeks of oral resveratrol treatment reduced the retinal vascular leakage area by 38% and restored GPX4 expression to 1.7 times the baseline level. In addition, Tang and colleagues provided significant observations (365). Their findings revealed that astragaloside IV (AS-IV) increased Sirt1/Nrf2 activity, ultimately resulting in increased GPX4 expression, which decreased retinal lipid peroxidation.

### 6.3 Glaucoma

Recent studies have shown that DFO is a unique compound that can penetrate the blood–retinal barrier with ease. Following ph-IOP-induced damage, it effectively binds to excess divalent iron in retinal tissue, preventing toxic accumulation. By mitigating iron overload, DFO safeguards retinal ganglion cells from ferroptosis and helps preserve normal vision. This protective mechanism highlights the potential of DFO as a therapeutic agent for retinal neuroprotection. Currently, multifunctional nanoparticles (NPsLip-1) have been developed and applied to manage acute glaucoma. These nanoparticles can significantly reduce lipid peroxidation products and restore mitochondrial function, thereby inhibiting ferroptosis. Ultimately, they increase the survival rate of RGCs in glaucoma models by 5.1-fold. Compared with traditional iron chelators (such as DFO), NPsLip-1 address issues related to rapid metabolism, poor targeting, and systemic toxicity (366).

Glutamate-induced cell surface *N*-methyl-d-aspartate receptors (NMDARs) stimulate cellular iron absorption by inducing the expression of Dexras1, a GTP-binding protein that increases DMT1-mediated iron uptake (316, 317). Sakamoto et al. reported that NMDA-induced intravitreal injections caused Fe^2+^ buildup and cellular death within RGCs (318). Iron-chelating agents protected RGCs from NMDA excitotoxicity-mediated cell death by reducing intracellular iron levels and alleviating oxidative stress in rodents (318, 319). Suppressing NCOA4 led to reduced FTH1 breakdown and decreased bivalent iron levels in the retina. This significantly reduced iron ion levels and alleviated RGC damage (315).

Glaucoma involves cytokine release driven by inflammation triggered by abnormal processing of amyloid precursor protein and subsequent deposition of Aβ and p-Tau in retinal ganglion cells, which disrupts iron homeostasis and leads to retinal iron overload. Iron triggers inflammation via the Fenton reaction, accelerating the formation and accumulation of amyloid-beta (Aβ). Therefore, the redox balance of RGCs interacts with iron ions in a self-amplifying feedback loop, exacerbating glaucoma-induced RGC death (320). Iron overload can damage mitochondria. Mitochondria in RGCs from individuals with glaucoma and murine glaucoma models were smaller, rounder, and more fragmented, suggesting a link between glaucoma, iron levels, and mitochondrial dysfunction (321). Mitochondrial dysfunction plays a critical role in RGC loss in the context of glaucoma (322). Ferroptosis inhibitors can promote RGC survival and protect retinal structure by maintaining mitochondrial function (323).

### 6.4 TAO

Ferroptosis inhibitors may be effective agents for the treatment of TAO, as they have been shown to prevent cell death by alleviating oxidative stress, having anti-fibrotic effects, and inhibiting lipid peroxidation. For example, sulfasalazine, a clinically used ferroptosis inducer, is very effective against thyroid cancer cells, indicating its possible use in TAO (367, 368). Selenium serves as a vital ferroptosis regulator and an effective therapy for TAO, as it can reduce lipid hydroperoxides and inhibit lipoxygenase (369). These inhibitors have the potential to mitigate orbital inflammation and tissue remodeling, both of which are pivotal indicators of TAO. They achieve this by scavenging free radicals produced via lipid peroxidation and inhibiting the buildup of harmful lipid peroxides. The IGF1R inhibitor teprotumumab has received FDA approval for managing active, moderate-to-severe TAO.

Studies on thyroid cancer cells have shown that modulating ferroptosis may have effects on cell survival and oxidative stress. Anaplastic thyroid carcinoma cells were found to be resistant to ferroptosis (370). Targeting the ferroptosis pathway may be a viable strategy for addressing TAO, a condition in which oxidative stress is a pivotal factor. Ferroptosis inducers such as erasin, in addition to iron-binding agents, have demonstrated potential in mitigating oxidative stress-induced damage and increasing cellular viability in preclinical studies (371). Ferroptosis plays a role in TAO pathogenesis. The overexpression of SIRT6 in thyroid cancer has been shown to increase sensitivity to ferroptosis through the autophagic degradation of ferritin dependent on nuclear receptor coactivator 4, suggesting that TAO may involve a similar mechanism (372). Ferroptosis-associated lncRNAs, such as LINC01140 and ZFHX4-AS1, have been found to be differentially expressed in TAO patients (373).

### 6.5 Corneal disorders

Studies have demonstrated that decreased levels of GPX4 in HCECs can lead to increased LDH levels, which can trigger ferroptosis (150). The application of the ferroptosis inhibitor Fer-1 could mitigate ferroptosis mediated by this pathway (152). Research has indicated that compared with wild-type mice, GPX4-deficient mice exhibit delayed corneal wound healing following epithelial injury, highlighting the role of GPX4 in corneal wound healing. The inhibition of ferroptosis protects against oxidative stress-related mortality in corneal epithelial cells when GPX4 is absent (153).

Studies have shown that the ferroptosis antagonist Fer-1 is promising for the treatment of multiple disorders linked to ferroptosis and that Fer-1 treatment can mitigate corneal opacity and neovascularization in alkali burn mouse models. Further studies have demonstrated that alkali burns trigger ferroptosis activation and the attack of ROS on mitochondria, which together facilitate the onset of ferroptosis within corneal tissue. The effectiveness of Fer-1 indicates that ferroptosis could be a therapeutic target in corneal alkali burn management. The experiment also showed that Fer-1 liposomes were significantly more effective than free Fer-1 at managing corneal alkali injury. In addition, *in vitro* and *in vivo* safety evaluations of Fer-1 liposomes were performed, and the results revealed that they had no significant toxic side effects (159, 160). Recent studies have revealed the role of AKR1C1 in protecting corneal epithelial cells against oxidative injury in DED (163). Ferroptosis triggered by high oxidative stress contributes to DED progression in a mouse *in vivo* DED model and immortalized HCECs. In addition, Nrf2 triggered AKR1C1 expression to attenuate iron toxicity-induced cell injury and inflammation in HCECs.

In a BK mouse model, the LEV + Fer-1 group presented lower levels of inflammatory factors, reduced corneal scarring, and lower Fe^3+^ levels. Significantly, the LEV + Fer-1 group exhibited elevated levels of GPX4 and SLC7A11. *In vitro* experiments revealed that Fer-1 administration reversed the LPS-mediated alterations in ROS, Fe^2+^, GPX4, and SLC7A11 levels in CSSCs. These findings indicate that ferroptosis significantly contributes to bacterial keratitis and that the inhibition of ferroptosis may help reduce inflammation, minimize corneal damage, and improve BK patient outcomes (299).

### 6.6 Others

Ferroptosis is a crucial mechanism of cell death in RIRI and affects cell types such as RGCs, PRs, and RPEs. This process involves the increased expression of iron-related genes (e.g., Acsl4 and Hmox1) and decreased GPX4 and SLC7A11 levels, along with Fe^2+^ and ROS buildup during the pathogenesis of RIRI (374). The inhibition of ferroptosis reduces both inflammatory factors and gliosis and protects retinal structures (375). Fer-1 can reduce RGC death by 60% and significantly increase the a/b-wave amplitude according to an electroretinogram in a RIRI mouse model. In addition, MT inhibited RIR-induced ferroptosis by decreasing ROS and Fe^2+^ levels and increasing GSH levels through the regulation of the p53/Slc7a11/Alox12 signaling pathway (375). DFO reduces the symptoms of RIRI and has been used clinically to treat iron overload disorders, and electroretinography (ERG) and visual evoked potentials (VEP) tests have been performed to confirm that retinal visual function improves in subjects after DFO treatment (274). Supramolecular DFO–crisaborole nanoparticles, which increase GPX4 levels, downregulate the expression of ferroptosis-related genes (Acsl4 and Hmox10), reduce the expression of inflammatory markers, and reduce ROS levels (279). It has been experimentally confirmed that ferroptosis plays an indispensable role in RP, and research on RP models has indicated that iron homeostasis regulators inhibit photoreceptor ferroptosis and prevent degeneration. Drugs such as zinc deferoxamine (283); iron chelators VK28, VAR10303, and DFO (230); and hepatostatin-1, along with the ferroptosis inhibitor DFO, effectively inhibit ferroptosis. For example, DFO decreased retinal iron deposition by 50% and increased photoreceptor survival by 1.8-fold in rd10 mice, and Fer-1 induced RGC and photoreceptor death by 60% and ERG b-wave amplitude recovery in rd1 mice. Qi-Shen-Tang (QST) slows RP progression in rd10 mice. It improves retinal tissue structure and performance, increases blood flow to the tissue, and decreases iron and MDA levels while increasing SOD and GSH concentrations. Additionally, QST (**Table 2**) contributes to antioxidant defense and protects against ferroptosis. Also, it safeguards retinal cells by activating the Nrf2/GPX4 signaling cascade (46).

**Table 2:** Ferroptosis-targeting agents for ocular diseases.

Ophthalmic diseases	Drug name	Target	Clinical phase	Mechanism	References
AMD	PEDF	GPX4/FTH1	ARPE-19/C57BL/6 mice	Upregulating the expression of GPX4 and FTH1	(376)
	ZnPP	Nrf2–SLC7A11–HO-1	ARPE-19/C57BL/6 mice	The specific inhibition of HO-1 overexpression has been determined to significantly block RPE ferroptosis	(245)
	Ferrostatin-1	GSH–GPX4 and FSP1–CoQ10–NADH	ARPE-19/C57BL/6J male	Restoring the impaired GSH–GPX4 and FSP1–CoQ10–NADH signaling in SIO-injured RPE	(53)
	Melatonin	PI3K/AKT/MDM2/P53 pathway	ARPE-19/male C57BL/6 strain mice	Suppressing cell death by ferroptosis in RPE via the PI3K/AKT/MDM2/P53 pathway	(377)
	Salidroside	Nrf2	ARPE-19/C57BL/6 mice	Exerting therapeutic effects by triggering Nrf2/SLC7A11/GPX4 signaling axis	(352)
	Deferiprone	Iron content	ARPE-19/C57BL/6 J mice	Suppressing Fe^2+^ significantly protected RPE cells against A2E–blue light-induced ferroptosis	(378)
	CircSPECC1	Oxidative stress	ARPE-19/C57BL/6J mice	Resisting oxidative stress injuries and maintaining lipid metabolism in RPE	(379)
	SDF-1α	SDF-1α/MTDH/SREBP1 axis	C57BL/6J mice/BMECs	Promoting transport of SREBP1 from ER to Golgi and SREBP1 maturation	(380)
	Crocin	KEAP1/NRF2/HO-1	Phase II clinical trial	Preventing an increase in Fe^2+^ levels and lipid peroxidation	(381)
DR	Corilagin	Nrf2	ARPE-19/C57BL/6 mice	Activating the Nrf2 antioxidant signaling pathway	(382)
	Fer-1	Cytosolic and lipid ROS, GSH/GPX4 axis	C57BL/6 mice/HRMECs	Decreasing lipid peroxidation	(383)
	1,8-Cineole	TXNIP/PPAR-γ	ARPE-19/C57BL/6 mice	Suppression of HG-induced ferroptosis in retinal tissue	(384)
	Sestrin2	STAT3/ER	ARPE-19/C57BL/6 mice	Inhibiting STAT3 phosphorylation and ER stress	(385)
	FLOT1	Nrf2	ARPE-19	Stimulating the SLC7A11/GPX4 pathway to inhibiting ferroptosis	(386)
	Ferrostatin-1	System Xc^−^	SD rats/ARPE-19	Improving the antioxidant capacity of the system Xc^−^	(387)
	ALKBH5	m^6^A–YTHDF1–ACSL4 axis	ARPE-19/C57BL/6 mice	Reducing ferroptosis through the m^6^A–YTHDF1–ACSL4 axis	(388)
	IQC	p53	C57BL/J mouse models/HRCECs	Downregulating the p53 signaling pathway, thereby reducing ferroptosis	(389)
	RSV	Nrf2/GPX4/PTGS2	SD rats/C57BL/6 mice	Decreasing MDA and PTGS2 and increasing cell viability, GSH, Nrf2, and GPX4	(390)
	Pifithrin-α	miR-214-3p/p53/SLC7A11/GPX4 axis	C57BL/6J mice/HRCECs	Mitigating cellular damage and Fe^2+^ accumulation	(391)
	Amygdalin	Nrf2/ARE	HRCECs	Activating the NRF2/ARE signaling pathway to inhibit ferroptosis	(392)
	RES	SIRT1/HMGB1	HRCECs	Upregulating the content of GSH and the protein expression of SLC7A11 and GPX4	(393)
	THPE2-F	GSH	ARPE-19/C57BL/6 J mice	Restoring GSH levels under hyperglycemic conditions and regulating iron levels	(394)
	miR-509-3p	GPX4	HRVECs/SD rats	Upregulating GPX4 to reduce ferroptosis	(395)
RP	FSE	p53/SLC7A11	661 W cells/C57/BL6 wild-type mice	Inhibiting ferroptosis of photoreceptors following oxidative stress via the p53/SLC7A11 pathway	(247)
	QST	Nrf2/GPX4	rd10 mice	Inhibiting ferroptosis by inhibiting the NRF2/GPX4 signaling pathway	(46)
Rb	YAP	Lipid peroxidation	Rb cell lines Y79 and RB3823	Inhibiting cell proliferation and promoting lipid peroxidation induced ferroptosis in Rb	(396)
Corneal disorders	PA eye drops	Iron content	BALB/c mice	Chelating ferrous ions for ferroptosis inhibition	(293)
	Fer-1	GPX4/SLC7A11	C57BL/6 mice/CSSCs	Restoring the alterations of ROS, Fe^2+^, GPX4, and SLC7A11, enhancing the prognosis of BK	(299)
	PTEN inhibitor/miR-23a-3p	PI3K/Akt/mTOR	CEnCs	Regulating PTEN/PI3K/Akt/mTOR signaling to inhibit ferroptosis	(397)
	Lip-1	Lipid peroxidation	HCECs/C57BL/6 mice	Scavenging lipid peroxyl radicals and inhibiting ferroptosis in corneal epithelial cells	(56)
	PC-DS NE	GPX4/ACSL4	SD rats	Scavenging ROS, inhibiting the expression of inflammatory cytokines and pro-angiogenic factors, and downregulating ferroptosis	(398)
	AS	System Xc^−^/GPX4 pathway	HCE-T cells	Reducing lipid peroxidation levels in HCE-T cells under hyperosmolarity and inhibiting ferroptosis through the system Xc^−^/GPX4 pathway	(399)
	Exo-Que	GRP78–GPX4/GRP78–ACSL4	HUCMSC/HCEC/HSFC/guinea pigs	Suppressing ferroptosis by modulating GRP78–ACSL4 and GRP78–GPX4 protein interactions, thus mitigating ECM remodeling and slowing myopia progression	(400)
DED	AKR1C1	Nrf2	C57BL/6 mice/HCEC	Upregulation of NRF2 increases AKR1C1 expression, alleviating ferroptosis-induced cell damage and inflammation	(304)
	CF@SNPs	p53–SLC7A11–GSH	Phase II clinical trial	Suppressing p53–SLC7A11–GSH-dependent ferroptosis	(306)
	AST	SLC7A11/GPX4	Routine clinical use	Upregulating SLC7A11/GPX4, inhibiting ferroptosis	(401)
	QXRMY	HMOX1/HIF-1α	Phase II clinical trial	Repressing ferroptosis through inhibiting the HMOX1/HIF-1 pathway	(402)
Glaucoma	Deferiprone	Iron content	C57BL/6 mice	Chelating the abnormally elevated iron ions in the retina and inhibiting the ferroptosis of RGCs	(314)
	SB202190	SLC7A11/GPX4	SD male rats/R28 cells	Regulating ferritin light chain, SAT1, and SLC7A11/GPX4 pathways	(403)
	Fer-1	Lipid peroxidation	C57BL/6J mice	Preventing the production of mitochondrial lipid peroxides in ONC retinas	(323)
	NPsLip-1	GPX4	R28cell/C57BL/6J mice	Inhibiting ferroptosis by upregulating GPX4	(366)
	FB-NPs	Nrf2	R28cell/C57BL/6J mice	Reducing ROS accumulation and modulating key ferroptosis markers (GPX4 and ACSL4)	(404)
	H_2_S	Iron content	C57BL/6J mice	Chelating iron, regulating iron metabolism, reducing oxidative stress, and mitigating ferroptosis	(405)
	Vitamin K1	Iron content	C57BL/6J mice	Modulating microglial ferroptosis, thereby alleviating acute ocular hypertension-induced retinal inflammation	(406)
Cataract	Melatonin	SIRT6/p-Nrf2/GPX4 SIRT6/COA4/FTH1	B-3 cell/SD rats	Inhibiting ferroptosis through the SIRT6/p-Nrf2/GPX4 and SIRT6/COA4/FTH1 pathways and delaying cataract formation caused by UVB exposure	(303)
	Liproxstatin-1	p53-GPX4/SLC7A11	SD rats	Inhibiting ferroptosis and reducing the incidence of DEX-induced GIC in rats	(333)
	Emodin	p53	LECs/SD rats	Alleviating damage to LECs by interfering with the p53-mediated ferroptosis pathway, attenuating DC disease	(407)
	Asparagine	Iron content	SD rats	Cutting down the accumulation of ferrous ions caused by naphthalene, protecting against naphthalene-induced cataracts by reducing ferroptosis	(408)
	ATX	GPX4	LECs/aged C57BL/6J mice	Alleviating human LECs damage by inhibiting ferroptosis and representing a promising therapeutic approach for age-related cataract	(409)

PEDF, pigment epithelium-derived factor; FTH1, ferritin heavy chain-1; SI, sodium iodate; PI3K, phosphatidylinositol-3-kinase; MDM2, murine double minute 2; SDF-1α, stromal cell-derived factor-1α; SREBP1, sterol regulatory element binding protein 1; ER, endoplasmic reticulum; HG, high glucose; TXNIP, thioredoxin-interacting protein; PPAR-γ, peroxisome proliferator-activated receptor γ; YTHDF1, YTH N6-methyladenosine RNA binding protein 1; ALKBH5, alkylation repair homolog protein 5; ACSL4, acyl-CoA synthetase long-chain family member 4; IQC, isoquercetin; RES, resveratrol; FSE, Fructus Lycii and *Salvia miltiorrhiza* Bunge extract; QST, Qi-Shen-Tang; YAP, Yes-associated protein; PA, phytic acid; CSSCs, corneal stromal stem cells; BK, bacterial keratitis; FECD, Fuchs endothelial corneal dystrophy; PTEN, phosphatase and tensin homolog; PI3K, phosphoinositide 3-kinase; mTOR, mechanistic target of rapamycin; Lip-1, Liproxstatin-1; CNV, corneal neovascularization; PC-DS NE, proanthocyanidin–diclofenac sodium nanozyme enzyme; AS, autologous serum; Exo-Que, exosome delivery system; ECM, extracellular matrix; AST, astaxanthin; QXRMY, Qingxuan Run Mu Yin; ONC, optic nerve crush; OGD/R, oxygen-glucose deprivation/reperfusion; FB-NPs, farrerol-loaded bilirubin nanoparticles; SIRT6, sirtuin 6; FTH1, ferritin heavy chain; UVB, ultraviolet B; GIC, glucocorticoid-induced posterior subcapsular cataracts; DEX, dexamethasone; DC, diabetic cataract; ATX, astaxanthin; BMECs, bEND.3 mouse brain microvascular endothelial cell line.

Ferroptosis has been demonstrated to be an important mechanism of programmed cell death in corneal epithelial cells and lacrimal acinar cells in DED. Ferrostatin-1, when applied as an eye drop, increased corneal epithelial survival by 60%, and the levels of Fe^2+^ and MDA decreased in hyperosmolar models. The mucolipid CF@SNPs, which encapsulate Fer-1 and cyclosporine A, mitigated oxidative stress-induced corneal epithelial death, and treatment with the CF@SNPs resulted in superior therapeutic efficacy and improvement in clinical DED parameters in a mouse model of DED, including the alleviation of corneal injury, the restoration of lacrimal gland structure, and the proliferation of goblet cells. The ocular surfaces of DED patients have high ROS levels, increased cell death-related marker expression, and dry eye stress-induced p53 upregulation, further triggering corneal epithelial ferroptosis. The combined administration of CsA and Fer-1 substantially alleviated corneal injury and DED marker upregulation, and CF@SNPs provide a promising solution to corneal injury in DED through the disruption of the inflammatory cascade, the generation of ROS, and ferroptosis. In addition, DFO can reduce intracellular Fe^2+^ and ROS levels and inhibit ferroptosis (401). Astaxanthin (AST) mitigates DED via the SLC7A11/GPX4 axis in corneal epithelial cells and mouse models under hyperosmolar stress.

RB1 deletion and p53 mutation affect both Rb pathogenesis and ferroptosis. Targeting ferroptosis is a novel strategy for the treatment of Rb. The induction of ferroptosis by autophagy may lower resistance in Rb cells, whereas 4-OI triggers ferroptosis via ferritin regulation (291). After 4-OI treatment, resistant Rb cell survival was reduced by 75%, and when combined with carboplatin, the *in vivo* tumor burden was reduced by 90%. Erastin reduced the IC50 of carboplatin-resistant cell lines by eightfold, but did not increase normal retinal toxicity.

High levels of markers with ferroptosis were detected in patients with myopic cataracts, diabetic cataracts, and age-related cataracts. Therefore, targeting ferroptosis is a novel approach for cataract therapy. Ferrostatin-1 and dasatinib (a DDR2-Src-Hippo inhibitor) significantly alleviated RSL3-induced nuclear cataracts and restored lens transparency in HMCs (332). An Nrf2 activator (hydralazine), which reduces ROS production in LECs in mice and humans, provides cytoprotection and delays lens opacification caused by aging and oxidative stress (410). Resveratrol may protect human LECs from oxidative damage by activating antioxidant enzymes (411). Lleó et al. demonstrated that melatonin could protect cells from damage due to H_2_O_2_ and white light LED-induced death (412). Experimentally, thiol antioxidants have shown significant promise in safeguarding LECs against oxidative damage and inhibiting cataract formation (413).

## 7 Limitations and challenges

Most recent studies on ferroptosis in ophthalmology have focused on RPECs or RGCs, but studies on the ability of Müller cells, vascular endothelial cells, PRs, and even corneal endothelial cells to induce ferroptosis are lacking, and comprehensive single-cell maps are lacking, leaving unresolved questions like the variations in the susceptibility of various cell types to ferroptosis, which can be parallel or mutually causal with apoptosis, necrosis, and pyroptosis (414). One study revealed that erastin promotes apoptosis in cancer cells. Iron-depleting compounds trigger ER stress, resulting in alternative cell death pathways (415). Notably, ferroptosis has no specific markers identified in the eye, making it difficult to distinguish it from other types of programmed cell death. The ocular microenvironment is complex, and the immune system differs from other sites, which also poses a challenge for treating ocular diseases with ferroptosis-targeting methods.

Multiple nodes, such as TF, TFR, DMT1, FT, and FPN1, can regulate iron levels; however, these proteins exhibit distinct expression patterns across retinopathies, and it is difficult to find “universal” intervention nodes. Systemic iron chelation may increase the risk of anemia and infection, and local delivery addresses the paradox of the penetration of the blood–retinal barrier and prolonged retinal retention. Currently, only static iron deposition (MRI or tissue iron staining) has been tested and does not reflect lipid peroxidation rates or GPX4 activity fluctuations in real time. Ophthalmologic examinations, such as retinal OCT-A or autofluorescence tests, do not specifically detect ferroptosis and are difficult to use for efficacy monitoring and patient stratification. This also poses a challenge to the timing and extent of intervention with ferroptosis treatment options. If OCT is combined with targeted probes or nano-contrast agents, functional imaging is expected to be realized in the future, enabling the labeling of ferroptosis and the assessment of retinal oxidative stress distribution. Detection of apoptosing retinal cells (DARC) is a method that combines retinal imaging technology with molecular biology to identify retinal apoptosis without the need for invasive methods using advanced confocal scanning laser ophthalmoscopy (cSLO) technology. Using a similar technical approach, imaging instruments can identify ferroptosis biomarkers in living systems. This facilitates crafting customized treatment plans derived from evaluations of noted cellular events.

The currently employed validated clinical models, such as photodamage, NaIO, and STZ mouse models, cannot completely mimic the chronic, multifactorial pathogenic environment of human AMD/DR, and the extrapolation of efficacy is questionable. As of now, the study of ferroptosis in the realm of ophthalmology is primarily limited to ARPE-19 cell cultures and rodent models of acute eye diseases. Techniques like photodamage can rapidly induce retinal deterioration within a matter of hours or days, but they do not adequately replicate the prolonged, recurring mild oxidative stress and the complex interplay of metabolic, inflammatory, and genetic factors found in human AMD/DR. This disconnect between “speedy” research models and “slow-moving” diseases has left a significant translational chasm, where promising results in animals tend to fade in actual patients. Consequently, there are only a few scattered case studies on ferroptosis-focused interventions in the field of ophthalmology. What we desperately need is a two-way approach from the laboratory to the patient’s bedside, focusing on long-term, multifactorial human populations. This approach should use their extensive data to improve animal studies and, ultimately, provide patients with effective, safe, and tailored treatments for ferroptosis.

The maximum tolerated dose and retinal toxicity threshold of iron chelators (DFO and deferiprone) have not yet been established. Additionally, whether long-term inhibition of ferroptosis by inhibitors interferes with the activity of iron-dependent enzymes, such as the mitochondrial respiratory chain, still requires phase I clinical data. Ethical and safety issues still need attention. As the first ferroptosis inhibitor to enter preclinical ophthalmic development, Fer-1 eye drops were originally limited by the drug’s inherent hydrophobicity. Researchers overcame this obstacle by preparing Fer-1-loaded liposomes via thin-film hydration. The resulting formulation shows sustained-release kinetics, markedly enhanced cellular uptake, and prolonged ocular surface residence time, thereby effectively ameliorating alkali burn-induced corneal injury. Safety evaluations, both *in vitro* and *in vivo*, revealed no evident toxicity. To date, 0.1%–0.3% Fer-1 liposomal eye drops have not exhibited significant off-target effects in acute or sub-acute models. In the future, long-term safety in larger cohorts will be a key endpoint in upcoming phase I trials.

## 8 Opportunities

Targeting ferroptosis opens new avenues for the treatment of ocular diseases. Ferroptosis has been shown to be involved in almost all irreversible blinding eye diseases. Recent experiments confirm that most of the common terminal events in AMD, RP, glaucoma, and DR are RPE or RGC ferroptosis, and single-cell sequencing confirms that ferroptosis signals appear earlier than apoptosis. Corneal epithelial ferroptosis after alkali burn and chemical injury is a major cause of corneal opacity and neovascularization. This treatment of these diseases via ferroptosis targeting provides a new direction. At present, preclinical experiments have been completed with related drugs. Fer-1 liposome eye drops can inhibit corneal opacity and increase bioavailability fivefold in a rabbit corneal alkali burn model, which indicates that agents that target ferroptosis have the potential for clinical translation and the treatment of ocular diseases.

Advanced methods such as single-cell sequencing and spatial transcriptomics have been used to map detailed gene expression and pathway networks for each cell in the retinal microenvironment during the development of AMD (416). This method enables the detection of cell subsets potentially undergoing ferroptosis, understanding the state of cells, and treating the symptoms. Cutting-edge research has highlighted the successful use of groundbreaking sustained-release technology known as the port delivery system (PDS). This implantable device enables the steady release of ranibizumab directly into the vitreous humor, offering a novel treatment approach for individuals suffering from nvAMD (417). This innovative system holds promise for safeguarding retinal cells against ferroptosis.

Using a technical approach similar to DARC, imaging devices can identify ferroptosis biomarkers in living systems and enable personalized treatment planning (418). Progress in merging bioinformatics, computational biology, and Artificial Intelligence (AI) has streamlined the identification of ferroptosis therapeutics for ophthalmic applications. State-of-the-art approaches include molecular dynamics modeling, quantum chemical computations, machine learning techniques, and network analyses. It can be a useful tool for investigating lipid membrane properties and the function of lipid peroxides in ferroptosis-induced cell death (419). These methods are essential for pathway analysis and cellular interactions to aid target selection and drug screening. Advanced AI systems are now able to create tailored predictions for AMD progression. Within the intricate world of the eye, these computational image analyses are reshaping our understanding of retinal aging and ailments, offering the chance for swift, early screening and targeted intervention. AI is expected to integrate multi-omics data in the future, providing novel tools for predicting ferroptosis-related gene expression and optimizing drug delivery (420).

To date, natural compounds have been shown to intervene in ferroptosis to alleviate eye diseases, and *Polygonatum odoratum* polysaccharide can increase the GSH content and SOD activity, thus playing an antioxidant role (419). Asiaticoside suppresses IL-6, IL-1β, and TNF-α expression via the NF-κB pathway while reducing oxidative stress. Madecassoside protects ARPE-2 cells against oxidative stress caused by H_2_O_2_ through the activation of the Nrf2/HO-2 signaling cascade. Matrine has diverse pharmacological properties (421). Aloperine mitigates oxidative stress in ARPE-19 cells triggered by H_2_O_2_, and these findings highlight the therapeutic potential of aloperine in the treatment of macular degeneration.

Liu et al. reported that fucoxanthin supplements have significant potential in preventing visible light-induced retinal damage (422). The primary active ingredient in *Astragalus membranaceus* is astragaloside IV, also known as AS-IV. This compound has the remarkable ability to block the process of oxidative tissue damage. By doing so, AS-IV lessens oxidative stress-related damage and increases cell viability in rat RGCs. New research findings suggest that AS-IV may inhibit pathological processes associated with DR (423). Puerarin, a key monomer in kudzu vine root flavones, has numerous effects, including anti-inflammatory and antioxidative stress effects. It is beneficial for treating ischemia–reperfusion tissue damage. Zhang et al. demonstrated that puerarin suppresses Nrf2/ERK pathway activation, mitigating inflammation and oxidative damage. *Ginkgo biloba* is a prevalent medicinal plant in Chinese medicine and has some efficacy in scavenging oxygen free radicals (424). Li et al. reported that *G. biloba* extract (GBE) significantly protected against t-BHP-triggered oxidative injury in human io-m1 cells. GBE protects human RGCs against oxidative stress and is promising for inhibiting retinopathies, especially DR (425). Other studies have revealed that pretreatment with GBE significantly mitigated oxidative lipid damage and cell death (necrosis/ferroptosis) and decreased the viability of RPECs exposed to t-BHP (426).

HCE ameliorates radiation-induced ferroptosis in LO2 cells via the Nrf2-xCT/GPX4 pathway (427).

Delphinidin was shown to play a key role in iron regulation and the upregulation of elements linked to the system Xc^−^ pathway while also curbing lipid oxidation, sustaining the integrity of cell membranes, and robustly safeguarding RPEs (661 W cells) from degeneration through the anti-ferroptosis pathway. Blueberry anthocyanins have also been shown to scavenge hydroxyl radicals, H_2_O_2_, 1,1-diphenyl-2-picrylhydrazyl (DPPH) radicals, and Fe^3+^ (428). Anthocyanins may be applied in ophthalmic diseases such as amblyopia, strabismus, cataracts, glaucoma, and retinopathy (429). Fucoidan shows therapeutic potential for AMD. Dörschmann et al. reported that fucoidan offers some defense against cell death triggered by erastin (430). In addition, the protective effect of fucoidan is linked to the maintenance of stable levels of the GPX4 protein, which is an important regulator of ferroptosis. These findings offer novel guidelines for managing eye disorders through ferroptosis through the uptake of natural compounds.

## References

[1] Nilsson DE . The diversity of eyes and vision. Annu Rev Vis Sci. (2021) 7:19–41. doi: 10.1146/annurev-vision-121820-074736, PMID: 34086478

[2] Baroncelli L Lunghi C . Neuroplasticity of the visual cortex: in sickness and in health. Exp Neurol. (2021) 335:113515. doi: 10.1016/j.expneurol.2020.113515, PMID: 33132181

[3] Yang QH Zhang Y Zhang XM Li XR . Prevalence of diabetic retinopathy, proliferative diabetic retinopathy and non-proliferative diabetic retinopathy in asian T2dm patients: A systematic review and meta-analysis. Int J Ophthalmol. (2019) 12:302–11. doi: 10.18240/ijo.2019.02.19, PMID: 30809489 PMC6376231

[4] Yuan H Chen S Duncan MR De Rivero Vaccari JP Keane RW Dalton Dietrich W . Ic100, a humanized therapeutic monoclonal anti-asc antibody alleviates oxygen-induced retinopathy in mice. Angiogenesis. (2024) 27:423–40. doi: 10.1007/s10456-024-09917-9, PMID: 38709389 PMC11303442

[5] Shi X Zhou XZ Chen G Luo WF Zhou C He TJ . Targeting the postsynaptic scaffolding protein psd-95 enhances bdnf signaling to mitigate depression-like behaviors in mice. Sci Signaling. (2024) 17:eadn4556. doi: 10.1126/scisignal.adn4556, PMID: 38687826 PMC11223518

[6] Tang D Chen X Kang R Kroemer G . Ferroptosis: molecular mechanisms and health implications. Cell Res. (2021) 31:107–25. doi: 10.1038/s41422-020-00441-1, PMID: 33268902 PMC8026611

[7] Zhuo Cai J Lan Yu Y Biao Yang Z Xun Xu X Chun Lv G Lian Xu C . Synergistic improvement of humus formation in compost residue by fenton-like and effective microorganism composite agents. Biores Technol. (2024) 400:130703. doi: 10.1016/j.biortech.2024.130703, PMID: 38631654

[8] Jiang X Stockwell BR Conrad M . Ferroptosis: mechanisms, biology and role in disease. Nat Rev Mol Cell Biol. (2021) 22:266–82. doi: 10.1038/s41580-020-00324-8, PMID: 33495651 PMC8142022

[9] Bannai S Kitamura E . Transport interaction of L-cystine and L-glutamate in human diploid fibroblasts in culture. J Biol Chem. (1980) 255:2372–6. doi: 10.1016/S0021-9258(19)85901-X, PMID: 7358676

[10] Dolma S Lessnick SL Hahn WC Stockwell BR . Identification of genotype-selective antitumor agents using synthetic lethal chemical screening in engineered human tumor cells. Cancer Cell. (2003) 3:285–96. doi: 10.1016/s1535-6108(03)00050-3, PMID: 12676586

[11] Yang WS Stockwell BR . Synthetic lethal screening identifies compounds activating iron-dependent, nonapoptotic cell death in oncogenic-ras-harboring cancer cells. Chem Biol. (2008) 15:234–45. doi: 10.1016/j.chembiol.2008.02.010, PMID: 18355723 PMC2683762

[12] Dixon SJ Lemberg KM Lamprecht MR Skouta R Zaitsev EM Gleason CE . Ferroptosis: an iron-dependent form of nonapoptotic cell death. Cell. (2012) 149:1060–72. doi: 10.1016/j.cell.2012.03.042, PMID: 22632970 PMC3367386

[13] Yang WS SriRamaratnam R Welsch ME Shimada K Skouta R Viswanathan VS . Regulation of ferroptotic cancer cell death by gpx4. Cell. (2014) 156:317–31. doi: 10.1016/j.cell.2013.12.010, PMID: 24439385 PMC4076414

[14] Shen Q Liang M Yang F Deng YZ Naqvi NI . Ferroptosis contributes to developmental cell death in rice blast. New Phytol. (2020) 227:1831–46. doi: 10.1111/nph.16636, PMID: 32367535

[15] Jenkins NL James SA Salim A Sumardy F Speed TP Conrad M . Changes in ferrous iron and glutathione promote ferroptosis and frailty in aging caenorhabditis elegans. eLife. (2020) 9:e56580. doi: 10.7554/eLife.56580, PMID: 32690135 PMC7373428

[16] Huo G Lin Y Liu L He Y Qu Y Liu Y . Decoding ferroptosis: transforming orthopedic disease management. Front Pharmacol. (2024) 15:1509172. doi: 10.3389/fphar.2024.1509172, PMID: 39712490 PMC11659002

[17] Andrews NC . Disorders of iron metabolism. N Engl J Med. (1999) 341:1986–95. doi: 10.1056/nejm199912233412607, PMID: 10607817

[18] Wang K Lin Y Zhou D Li P Zhao X Han Z . Unveiling ferroptosis: A new frontier in skin disease research. Front Immunol. (2024) 15:1485523. doi: 10.3389/fimmu.2024.1485523, PMID: 39430757 PMC11486644

[19] Maiorino M Conrad M Ursini F . Gpx4, lipid peroxidation, and cell death: discoveries, rediscoveries, and open issues. Antioxid Redox Signal. (2018) 29:61–74. doi: 10.1089/ars.2017.7115, PMID: 28462584

[20] He Y Lin Y Song J Song M Nie X Sun H . From mechanisms to medicine: ferroptosis as a therapeutic target in liver disorders. Cell Communicat Signal: CCS. (2025) 23:125. doi: 10.1186/s12964-025-02121-2, PMID: 40055721 PMC11889974

[21] Yang Y Lin Y Han Z Wang B Zheng W Wei L . Ferroptosis: A novel mechanism of cell death in ophthalmic conditions. Front Immunol. (2024) 15:1440309. doi: 10.3389/fimmu.2024.1440309, PMID: 38994366 PMC11236620

[22] Zhao T Guo X Sun Y . Iron accumulation and lipid peroxidation in the aging retina: implication of ferroptosis in age-related macular degeneration. Aging Dis. (2021) 12:529–51. doi: 10.14336/ad.2020.0912, PMID: 33815881 PMC7990372

[23] Kajarabille N Latunde-Dada GO . Programmed cell-death by ferroptosis: antioxidants as mitigators. Int J Mol Sci. (2019) 20:4968. doi: 10.3390/ijms20194968, PMID: 31597407 PMC6801403

[24] Moiseyev G Takahashi Y Chen Y Gentleman S Redmond TM Crouch RK . Rpe65 is an iron(Ii)-dependent isomerohydrolase in the retinoid visual cycle. J Biol Chem. (2006) 281:2835–40. doi: 10.1074/jbc.M508903200, PMID: 16319067

[25] Chen H Han Z Wang Y Su J Lin Y Cheng X . Targeting ferroptosis in bone-related diseases: facts and perspectives. J Inflammation Res. (2023) 16:4661–77. doi: 10.2147/jir.S432111, PMID: 37872954 PMC10590556

[26] Picard E Daruich A Youale J Courtois Y Behar-Cohen F . From rust to quantum biology: the role of iron in retina physiopathology. Cells. (2020) 9:705. doi: 10.3390/cells9030705, PMID: 32183063 PMC7140613

[27] Han Z Luo Y Chen H Zhang G You L Zhang M . A deep insight into ferroptosis in renal disease: facts and perspectives. Kidney Dis (Basel Switzerland). (2024) 10:224–36. doi: 10.1159/000538106, PMID: 38835406 PMC11149998

[28] Liu D Liu Z Liao H Chen ZS Qin B . Ferroptosis as a potential therapeutic target for age-related macular degeneration. Drug Discov Today. (2024) 29:103920. doi: 10.1016/j.drudis.2024.103920, PMID: 38369100

[29] Totsuka K Ueta T Uchida T Roggia MF Nakagawa S Vavvas DG . Oxidative stress induces ferroptotic cell death in retinal pigment epithelial cells. Exp Eye Res. (2019) 181:316–24. doi: 10.1016/j.exer.2018.08.019, PMID: 30171859 PMC7418497

[30] Huang S Liu K Su Y Wang F Feng T . Research progress of ferroptosis in glaucoma and optic nerve damage. Mol Cell Biochem. (2023) 478:721–7. doi: 10.1007/s11010-022-04545-7, PMID: 36053395

[31] Gaschler MM Stockwell BR . Lipid peroxidation in cell death. Biochem Biophys Res Commun. (2017) 482:419–25. doi: 10.1016/j.bbrc.2016.10.086, PMID: 28212725 PMC5319403

[32] You H Wang L Bu F Meng H Huang C Fang G . Ferroptosis: shedding light on mechanisms and therapeutic opportunities in liver diseases. Cells. (2022) 11:3301. doi: 10.3390/cells11203301, PMID: 36291167 PMC9600232

[33] Tang W Guo J Liu W Ma J Xu G . Ferrostatin-1 attenuates ferroptosis and protects the retina against light-induced retinal degeneration. Biochem Biophys Res Commun. (2021) 548:27–34. doi: 10.1016/j.bbrc.2021.02.055, PMID: 33631670

[34] Zhao D Li W Han Z Wang Z Li D Li W . Exploring the role of ferroptosis in esophageal cancer: mechanisms and therapeutic implications. Cell Death Discov. (2025) 11:405. doi: 10.1038/s41420-025-02696-2, PMID: 40855044 PMC12379297

[35] Zhang HL Hu BX Li ZL Du T Shan JL Ye ZP . Pkcβii phosphorylates acsl4 to amplify lipid peroxidation to induce ferroptosis. Nat Cell Biol. (2022) 24:88–98. doi: 10.1038/s41556-021-00818-3, PMID: 35027735

[36] Chen H Han Z Su J Song X Ma Q Lin Y . Ferroptosis and hepatocellular carcinoma: the emerging role of lncrnas. Front Immunol. (2024) 15:1424954. doi: 10.3389/fimmu.2024.1424954, PMID: 38846953 PMC11153672

[37] Liu C Sun W Zhu T Shi S Zhang J Wang J . Glia maturation factor-β Induces ferroptosis by impairing chaperone-mediated autophagic degradation of acsl4 in early diabetic retinopathy. Redox Biol. (2022) 52:102292. doi: 10.1016/j.redox.2022.102292, PMID: 35325805 PMC8942824

[38] Xiao R Han Z Jia P Li P Gong M Cai Y . Ferroptosis and bone health: bridging the gap between mechanisms and therapy. Front Immunol. (2025) 16:1634516. doi: 10.3389/fimmu.2025.1634516, PMID: 40740786 PMC12307186

[39] Lapaquette P Terrat S Proukhnitzky L Martine L Grégoire S Buteau B . Long-term intake of lactobacillus helveticus enhances bioavailability of omega-3 fatty acids in the mouse retina. NPJ Biofilms Microbiomes. (2024) 10:4. doi: 10.1038/s41522-023-00474-5, PMID: 38238339 PMC10796366

[40] Perus M Courtaut F Pais De Barros JP Aires V Hermetet F Delmas D . Vegf-R2/cav-1 interaction induced by resveratrol/eicosapentaenoic acid/docosahexaenoic acid-enriched formulation through functional detergent-resistant membranes is associated with decreased vegf-a release in arpe-19 cells. Mol Nutr Food Res. (2024) 68:e2300893. doi: 10.1002/mnfr.202300893, PMID: 38763919

[41] Shao M Jiang Q Shen C Liu Z Qiu L . Sinapine induced ferroptosis in non-small cell lung cancer cells by upregulating transferrin/transferrin receptor and downregulating slc7a11. Gene. (2022) 827:146460. doi: 10.1016/j.gene.2022.146460, PMID: 35358657

[42] Xue P Zhuang H Bai T Zeng X Deng J Shao S . Iron (Ii)-based metal-organic framework nanozyme for boosting tumor ferroptosis through inhibiting DNA damage repair and system xc(). J Nanobiotechnolo. (2024) 22:228. doi: 10.1186/s12951-024-02508-2, PMID: 38715049 PMC11077818

[43] Huang X Yang X Zhang M Li T Zhu K Dong Y . Selenoi functions as a key modulator of ferroptosis pathway in colitis and colorectal cancer. Adv Sci (Weinh). (2024) 11:e2404073. doi: 10.1002/advs.202404073, PMID: 38757622 PMC11267378

[44] Wang Y Huang X Luo G Xu Y Deng X Lin Y . The aging lung: microenvironment, mechanisms, and diseases. Front Immunol. (2024) 15:1383503. doi: 10.3389/fimmu.2024.1383503, PMID: 38756780 PMC11096524

[45] Li J Cao F Yin HL Huang ZJ Lin ZT Mao N . Ferroptosis: past, present and future. Cell Death Dis. (2020) 11:88. doi: 10.1038/s41419-020-2298-2, PMID: 32015325 PMC6997353

[46] Xiong M Ou C Yu C Qiu J Lu J Fu C . Qi-shen-tang alleviates retinitis pigmentosa by inhibiting ferroptotic features via the nrf2/gpx4 signaling pathway. Heliyon. (2023) 9:e22443. doi: 10.1016/j.heliyon.2023.e22443, PMID: 38034716 PMC10687062

[47] Lu L Oveson BC Jo YJ Lauer TW Usui S Komeima K . Increased expression of glutathione peroxidase 4 strongly protects retina from oxidative damage. Antioxid Redox Signal. (2009) 11:715–24. doi: 10.1089/ars.2008.2171, PMID: 18823256 PMC2787833

[48] Azuma K Koumura T Iwamoto R Matsuoka M Terauchi R Yasuda S . Mitochondrial glutathione peroxidase 4 is indispensable for photoreceptor development and survival in mice. J Biol Chem. (2022) 298:101824. doi: 10.1016/j.jbc.2022.101824, PMID: 35288190 PMC8980337

[49] Niu T Shi X Liu X Wang H Liu K Xu Y . Porous se@Sio(2) nanospheres alleviate diabetic retinopathy by inhibiting excess lipid peroxidation and inflammation. Mol Med (Cambridge Mass). (2024) 30:24. doi: 10.1186/s10020-024-00785-z, PMID: 38321393 PMC10848509

[50] Yang N Pan X Zhou X Liu Z Yang J Zhang J . Biomimetic nanoarchitectonics with chitosan nanogels for collaborative induction of ferroptosis and anticancer immunity for cancer therapy. Adv Healthcare Mat. (2024) 13:e2302752. doi: 10.1002/adhm.202302752, PMID: 37975280

[51] Chen H Wang C Liu Z He X Tang W He L . Ferroptosis and its multifaceted role in cancer: mechanisms and therapeutic approach. Antioxid (Basel Switzerland). (2022) 11:1504. doi: 10.3390/antiox11081504, PMID: 36009223 PMC9405274

[52] Nakamura T Hipp C Santos Dias Mourão A Borggräfe J Aldrovandi M Henkelmann B . Phase separation of fsp1 promotes ferroptosis. Nature. (2023) 619:371–7. doi: 10.1038/s41586-023-06255-6, PMID: 37380771 PMC10338336

[53] Yang M Tsui MG Tsang JKW Goit RK Yao KM So KF . Involvement of fsp1-coq(10)-nadh and gsh-gpx-4 pathways in retinal pigment epithelium ferroptosis. Cell Death Dis. (2022) 13:468. doi: 10.1038/s41419-022-04924-4, PMID: 35585057 PMC9117320

[54] Liu T Zhao J Lin C . Sprouty-related proteins with evh1 domain (Spred2) prevents high-glucose induced endothelial-mesenchymal transition and endothelial injury by suppressing mapk activation. Bioengineered. (2022) 13:13882–92. doi: 10.1080/21655979.2022.2086351, PMID: 35707829 PMC9275976

[55] Mao C Liu X Zhang Y Lei G Yan Y Lee H . Dhodh-mediated ferroptosis defence is a targetable vulnerability in cancer. Nature. (2021) 593:586–90. doi: 10.1038/s41586-021-03539-7, PMID: 33981038 PMC8895686

[56] Wu MF Peng X Zhang MC Guo H Xie HT . Ferroptosis and panoptosis under hypoxia pivoting on the crosstalk between dhodh and gpx4 in corneal epithelium. Free Radic Biol Med. (2025) 228:173–82. doi: 10.1016/j.freeradbiomed.2024.12.050, PMID: 39761766

[57] Schmidl D Hommer N Kallab M Schlatter A Nadvornik C Obermayr F . Safety and tolerability of kio-101 eye drops in healthy volunteers and patients with ocular surface disease-a phase I study. Pharmaceutics. (2024) 16:367. doi: 10.3390/pharmaceutics16030367, PMID: 38543260 PMC10974994

[58] Kraft VAN Bezjian CT Pfeiffer S Ringelstetter L Müller C Zandkarimi F . Gtp cyclohydrolase 1/tetrahydrobiopterin counteract ferroptosis through lipid remodeling. ACS Cent Sci. (2020) 6:41–53. doi: 10.1021/acscentsci.9b01063, PMID: 31989025 PMC6978838

[59] Lohmann K Redin C Tönnies H Bressman SB Subero JIM Wiegers K . Complex and dynamic chromosomal rearrangements in a family with seemingly non-mendelian inheritance of dopa-responsive dystonia. JAMA Neurol. (2017) 74:806–12. doi: 10.1001/jamaneurol.2017.0666, PMID: 28558098 PMC5710536

[60] Cai B Cai JP Luo YL Chen C Zhang S . The specific roles of jak/stat signaling pathway in sepsis. Inflammation. (2015) 38:1599–608. doi: 10.1007/s10753-015-0135-z, PMID: 25676437

[61] Chen Y Fang ZM Yi X Wei X Jiang DS . The interaction between ferroptosis and inflammatory signaling pathways. Cell Death Dis. (2023) 14:205. doi: 10.1038/s41419-023-05716-0, PMID: 36944609 PMC10030804

[62] Moutsopoulos HM . Sjögren's syndrome: autoimmune epithelitis. Clin Immunol Immunopathol. (1994) 72:162–5. doi: 10.1006/clin.1994.1123, PMID: 8050187

[63] Gandolfo S Ciccia F . Jak/stat pathway targeting in primary sjögren syndrome. Rheumatol Immunol Res. (2022) 3:95–102. doi: 10.2478/rir-2022-0017, PMID: 36788973 PMC9895869

[64] Zhang Q Lenardo MJ Baltimore D . 30 years of nf-κb: A blossoming of relevance to human pathobiology. Cell. (2017) 168:37–57. doi: 10.1016/j.cell.2016.12.012, PMID: 28086098 PMC5268070

[65] Liang WJ Yang HW Liu HN Qian W Chen XL . Hmgb1 upregulates nf-kb by inhibiting ikb-α and associates with diabetic retinopathy. Life Sci. (2020) 241:117146. doi: 10.1016/j.lfs.2019.117146, PMID: 31816325

[66] Palazzo I Todd LJ Hoang TV Reh TA Blackshaw S Fischer AJ . Nfkb-signaling promotes glial reactivity and suppresses müller glia-mediated neuron regeneration in the mammalian retina. Glia. (2022) 70:1380–401. doi: 10.1002/glia.24181, PMID: 35388544 PMC9585486

[67] Jiang K Zhang F Chen Y Li X Zhao X Jiang P . Fosfenopril attenuates inflammatory response in diabetic dry eye models by inhibiting the tlr4/nf-κb/nlrp3 signaling pathway. Invest Ophthalmol Vis Sci. (2024) 65:2. doi: 10.1167/iovs.65.6.2, PMID: 38829670 PMC11156208

[68] Pan S Liu M Xu H Chuan J Yang Z . Lipopolysaccharide activating nf-kb signaling by regulates htra1 expression in human retinal pigment epithelial cells. Molecules. (2023) 28:2236. doi: 10.3390/molecules28052236, PMID: 36903482 PMC10004666

[69] Sun X Ou Z Chen R Niu X Chen D Kang R . Activation of the P62-keap1-nrf2 pathway protects against ferroptosis in hepatocellular carcinoma cells. Hepatol (Baltimore Md). (2016) 63:173–84. doi: 10.1002/hep.28251, PMID: 26403645 PMC4688087

[70] Wang D Tang L Zhang Y Ge G Jiang X Mo Y . Regulatory pathways and drugs associated with ferroptosis in tumors. Cell Death Dis. (2022) 13:544. doi: 10.1038/s41419-022-04927-1, PMID: 35688814 PMC9187756

[71] Jiang L Kon N Li T Wang SJ Su T Hibshoosh H . Ferroptosis as a P53-mediated activity during tumour suppression. Nature. (2015) 520:57–62. doi: 10.1038/nature14344, PMID: 25799988 PMC4455927

[72] Suzuki T Yamamoto M . Molecular basis of the keap1-nrf2 system. Free Radic Biol Med. (2015) 88:93–100. doi: 10.1016/j.freeradbiomed.2015.06.006, PMID: 26117331

[73] Zhang H Davies KJA Forman HJ . Oxidative stress response and nrf2 signaling in aging. Free Radic Biol Med. (2015) 88:314–36. doi: 10.1016/j.freeradbiomed.2015.05.036, PMID: 26066302 PMC4628850

[74] Xu XR Yu HT Yang Y Hang L Yang XW Ding SH . Quercetin phospholipid complex significantly protects against oxidative injury in arpe-19 cells associated with activation of nrf2 pathway. Eur J Pharmacol. (2016) 770:1–8. doi: 10.1016/j.ejphar.2015.11.050, PMID: 26643168

[75] Xu Z Wei Y Gong J Cho H Park JK Sung ER . Nrf2 plays a protective role in diabetic retinopathy in mice. Diabetologia. (2014) 57:204–13. doi: 10.1007/s00125-013-3093-8, PMID: 24186494 PMC4039644

[76] Batliwala S Xavier C Liu Y Wu H Pang IH . Involvement of nrf2 in ocular diseases. Oxid Med Cell Longev. (2017) 2017:1703810. doi: 10.1155/2017/1703810, PMID: 28473877 PMC5394909

[77] Von Otter M Landgren S Nilsson S Zetterberg M Celojevic D Bergström P . Nrf2-encoding nfe2l2 haplotypes influence disease progression but not risk in alzheimer's disease and age-related cataract. Mech Ageing Dev. (2010) 131:105–10. doi: 10.1016/j.mad.2009.12.007, PMID: 20064547

[78] Zhang YN Ouyang WJ Hu JY Liu ZG . Targeting nrf2 signaling in dry eye. Int J Ophthalmol. (2024) 17:1911–20. doi: 10.18240/ijo.2024.10.19, PMID: 39430029 PMC11422368

[79] Yuan M He Q Xiang W Deng Y Lin S Zhang R . Natural compounds efficacy in ophthalmic diseases: A new twist impacting ferroptosis. BioMed Pharmacother. (2024) 172:116230. doi: 10.1016/j.biopha.2024.116230, PMID: 38350366

[80] Chu B Kon N Chen D Li T Liu T Jiang L . Alox12 is required for P53-mediated tumour suppression through a distinct ferroptosis pathway. Nat Cell Biol. (2019) 21:579–91. doi: 10.1038/s41556-019-0305-6, PMID: 30962574 PMC6624840

[81] Galluzzi L Bravo-San Pedro JM Kroemer G . Ferroptosis in P53-dependent oncosuppression and organismal homeostasis. Cell Death Differ. (2015) 22:1237–8. doi: 10.1038/cdd.2015.54, PMID: 26143748 PMC4495364

[82] Xie T Bai Z Chen Z Liang H Liu T Lam LK . Inhibition of ferroptosis ameliorates hypertensive nephropathy through P53/nrf2/P21 pathway by taohongsiwu decoction: based on network pharmacology and experimental validation. J Ethnopharmacol. (2023) 312:116506. doi: 10.1016/j.jep.2023.116506, PMID: 37086874

[83] Wang S Zhao Y Yao F Wei P Ma L Zhang S . An anti-gd2 aptamer-based bifunctional spherical nucleic acid nanoplatform for synergistic therapy targeting mdm2 for retinoblastoma. Biomedicine Pharmacother = Biomedecine Pharmacother. (2024) 174:116437. doi: 10.1016/j.biopha.2024.116437, PMID: 38522240

[84] Chu WK Choi HL Bhat AK Jhanji V . Pterygium: new insights. Eye (Lond). (2020) 34:1047–50. doi: 10.1038/s41433-020-0786-3, PMID: 32029918 PMC7413326

[85] Cheng Y Zhang M Xu R Fu L Xue M Xu C . P53 accelerates endothelial cell senescence in diabetic retinopathy by enhancing foxo3a ubiquitylation and degradation via ube2l6. Exp Gerontol. (2024) 188:112391. doi: 10.1016/j.exger.2024.112391, PMID: 38437929

[86] Zhang S Wu J Wang L Mu L Xu X Li J . Sirt1/P53 in retinal pigment epithelial cells in diabetic retinopathy: A gene co-expression analysis and he-ying-qing-re formula treatment. Front Mol Biosci. (2024) 11:1366020. doi: 10.3389/fmolb.2024.1366020, PMID: 38633216 PMC11021775

[87] Lee H Zandkarimi F Zhang Y Meena JK Kim J Zhuang L . Energy-stress-mediated ampk activation inhibits ferroptosis. Nat Cell Biol. (2020) 22:225–34. doi: 10.1038/s41556-020-0461-8, PMID: 32029897 PMC7008777

[88] Song X Zhu S Chen P Hou W Wen Q Liu J . Ampk-mediated becn1 phosphorylation promotes ferroptosis by directly blocking system X(C)(-) activity. Curr Biol. (2018) 28:2388–99.e5. doi: 10.1016/j.cub.2018.05.094, PMID: 30057310 PMC6081251

[89] Zhang Z Yao Z Wang L Ding H Shao J Chen A . Activation of ferritinophagy is required for the rna-binding protein elavl1/hur to regulate ferroptosis in hepatic stellate cells. Autophagy. (2018) 14:2083–103. doi: 10.1080/15548627.2018.1503146, PMID: 30081711 PMC6984765

[90] Fomo KN Perumal N Manicam C Pfeiffer N Grus FH . Neuroretinal cell culture model as a tool for the development of new therapeutic approaches for oxidative stress-induced ocular diseases, with a focus on glaucoma. Cells. (2024) 13:775. doi: 10.3390/cells13090775, PMID: 38727311 PMC11083839

[91] Dieguez HH Romeo HE Alaimo A Bernal Aguirre NA Calanni JS Adán Aréan JS . Mitochondrial quality control in non-exudative age-related macular degeneration: from molecular mechanisms to structural and functional recovery. Free Radical Biol Med. (2024) 219:17–30. doi: 10.1016/j.freeradbiomed.2024.03.024, PMID: 38579938

[92] Guo Y Zhang H Zhao Z Luo X Zhang M Bu J . Hyperglycemia induces meibomian gland dysfunction. Invest Ophthalmol Vis Sci. (2022) 63:30. doi: 10.1167/iovs.63.1.30, PMID: 35072689 PMC8802017

[93] Peng F Jiang D Xu W Sun Y Zha Z Tan X . Ampk/mff activation: role in mitochondrial fission and mitophagy in dry eye. Invest Ophthalmol Vis Sci. (2022) 63:18. doi: 10.1167/iovs.63.12.18, PMID: 36374514 PMC9669805

[94] Zhang Z Jing J Ye Y Chen Z Jing Y Li S . Characterization of the dual functional effects of heat shock proteins (Hsps) in cancer hallmarks to aid development of hsp inhibitors. Genome Med. (2020) 12:101. doi: 10.1186/s13073-020-00795-6, PMID: 33225964 PMC7682077

[95] Feng Y Chen Q Jin C Ruan Y Chen Q Lin W . Microwave-activated cu-doped zirconium metal-organic framework for a highly effective combination of microwave dynamic and thermal therapy. J Controlled Release. (2023) 361:102–14. doi: 10.1016/j.jconrel.2023.07.046, PMID: 37532150

[96] Sun X Ou Z Xie M Kang R Fan Y Niu X . Hspb1 as a novel regulator of ferroptotic cancer cell death. Oncogene. (2015) 34:5617–25. doi: 10.1038/onc.2015.32, PMID: 25728673 PMC4640181

[97] Zhu S Zhang Q Sun X Zeh HJ 3rd Lotze MT Kang R . Hspa5 regulates ferroptotic cell death in cancer cells. Cancer Res. (2017) 77:2064–77. doi: 10.1158/0008-5472.Can-16-1979, PMID: 28130223 PMC5392369

[98] Zhou Y Liao J Mei Z Liu X Ge J . Insight into crosstalk between ferroptosis and necroptosis: novel therapeutics in ischemic stroke. Oxid Med Cell Longev. (2021) 2021:9991001. doi: 10.1155/2021/9991001, PMID: 34257829 PMC8257382

[99] Huang L Hong Y Fu X Tan H Chen Y Wang Y . The role of the microbiota in glaucoma. Mol Aspects Med. (2023) 94:101221. doi: 10.1016/j.mam.2023.101221, PMID: 37866106

[100] Saini C Jiang S Devlin J Pan L Tang Y Tang J . Association between hsp-specific T-cell counts and retinal nerve fiber layer thickness in patients with primary open-angle glaucoma. Ophthalmol Sci. (2023) 3:100310. doi: 10.1016/j.xops.2023.100310, PMID: 37197701 PMC10183658

[101] Geyer O Levo Y . Glaucoma is an autoimmune disease. Autoimmun Rev. (2020) 19:102535. doi: 10.1016/j.autrev.2020.102535, PMID: 32234407

[102] Tsai T Grotegut P Reinehr S Joachim SC . Role of heat shock proteins in glaucoma. Int J Mol Sci. (2019) 20:5160. doi: 10.3390/ijms20205160, PMID: 31635205 PMC6834184

[103] O'Reilly AM Currie RW Clarke DB . Hspb1 (Hsp 27) expression and neuroprotection in the retina. Mol Neurobiol. (2010) 42:124–32. doi: 10.1007/s12035-010-8143-3, PMID: 20514530

[104] Alven A Lema C Redfern RL . Impact of low humidity on damage-associated molecular patterns at the ocular surface during dry eye disease. Optom Vis Sci. (2021) 98:1231–8. doi: 10.1097/opx.0000000000001802, PMID: 34510151 PMC8585693

[105] Feng YQ Liu X Zuo N Yu MB Bian WM Han BQ . Nad(+) precursors promote the restoration of spermatogenesis in busulfan-treated mice through inhibiting sirt2-regulated ferroptosis. Theranostics. (2024) 14:2622–36. doi: 10.7150/thno.92416, PMID: 38646657 PMC11024856

[106] Bersuker K Hendricks JM Li Z Magtanong L Ford B Tang PH . The coq oxidoreductase fsp1 acts parallel to gpx4 to inhibit ferroptosis. Nature. (2019) 575:688–92. doi: 10.1038/s41586-019-1705-2, PMID: 31634900 PMC6883167

[107] Alves F Lane D Nguyen TPM Bush AI Ayton S . In defence of ferroptosis. Signal Transduct Target Ther. (2025) 10:2. doi: 10.1038/s41392-024-02088-5, PMID: 39746918 PMC11696223

[108] Liu N Wu WL Wan XR Wang J Huang JN Jiang YY . Regulation of fsp1 myristoylation by nadph: A novel mechanism for ferroptosis inhibition. Redox Biol. (2024) 73:103176. doi: 10.1016/j.redox.2024.103176, PMID: 38705094 PMC11074979

[109] Liao J Lai Z Huang G Lin J Huang W Qin Y . Setanaxib mitigates oxidative damage following retinal ischemia-reperfusion via nox1 and nox4 inhibition in retinal ganglion cells. Biomedicine Pharmacother = Biomedecine Pharmacother. (2024) 170:116042. doi: 10.1016/j.biopha.2023.116042, PMID: 38118351

[110] Guo M Liu T Miao Y Pan X Liu B . Role of nadph oxidase 4 on dry eye syndrome in mice. J Ocul Pharmacol Ther. (2024) 40:452–8. doi: 10.1089/jop.2024.0002, PMID: 38669123

[111] Shi X Li P Herb M Liu H Wang M Wang X . Pathological high intraocular pressure induces glial cell reactive proliferation contributing to neuroinflammation of the blood-retinal barrier via the nox2/et-1 axis-controlled erk1/2 pathway. J Neuroinflamm. (2024) 21:105. doi: 10.1186/s12974-024-03075-x, PMID: 38649885 PMC11034147

[112] Liu Y Wang W Li Y Xiao Y Cheng J Jia J . The 5-lipoxygenase inhibitor zileuton confers neuroprotection against glutamate oxidative damage by inhibiting ferroptosis. Biol Pharm Bull. (2015) 38:1234–9. doi: 10.1248/bpb.b15-00048, PMID: 26235588

[113] Liu T Shu J Liu Y Xie J Li T Li H . Atorvastatin attenuates ferroptosis-dependent myocardial injury and inflammation following coronary microembolization via the hif1a/ptgs2 pathway. Front Pharmacol. (2022) 13:1057583. doi: 10.3389/fphar.2022.1057583, PMID: 36569299 PMC9772535

[114] Sun Q Liu D Cui W Cheng H Huang L Zhang R . Cholesterol mediated ferroptosis suppression reveals essential roles of coenzyme Q and squalene. Commun Biol. (2023) 6:1108. doi: 10.1038/s42003-023-05477-8, PMID: 37914914 PMC10620397

[115] Eberhard Y McDermott SP Wang X Gronda M Venugopal A Wood TE . Chelation of intracellular iron with the antifungal agent ciclopirox olamine induces cell death in leukemia and myeloma cells. Blood. (2009) 114:3064–73. doi: 10.1182/blood-2009-03-209965, PMID: 19589922

[116] Zilka O Shah R Li B Friedmann Angeli JP Griesser M Conrad M . On the mechanism of cytoprotection by ferrostatin-1 and liproxstatin-1 and the role of lipid peroxidation in ferroptotic cell death. ACS Cent Sci. (2017) 3:232–43. doi: 10.1021/acscentsci.7b00028, PMID: 28386601 PMC5364454

[117] Xiang X Xu M Liu L Meng N Lei Y Feng Y . Liproxstatin-1 attenuates acute hypertriglyceridemic pancreatitis through inhibiting ferroptosis in rats. Sci Rep. (2024) 14:9548. doi: 10.1038/s41598-024-60159-7, PMID: 38664508 PMC11045844

[118] Zhou Y Hu T Zeng H Lin L Xie H Lin R . Naringenin inhibits ferroptosis in renal tubular epithelial cells of diabetic nephropathy through sirt1/foxo3a signaling pathway. Drug Dev Res. (2025) 86:e70044. doi: 10.1002/ddr.70044, PMID: 39799560

[119] Long Z Yu X Li S Cheng N Huo C Zhang X . Sakuranetin prevents acetaminophen-induced liver injury via nrf2-induced inhibition of hepatocyte ferroptosis. Drug Des Devel Ther. (2025) 19:159–71. doi: 10.2147/dddt.S497817, PMID: 39816848 PMC11733203

[120] Tuo QZ Masaldan S Southon A Mawal C Ayton S Bush AI . Characterization of selenium compounds for anti-ferroptotic activity in neuronal cells and after cerebral ischemia-reperfusion injury. Neurotherapeutics. (2021) 18:2682–91. doi: 10.1007/s13311-021-01111-9, PMID: 34498224 PMC8804037

[121] Zhang H Wang Y Wang S Xue X Huang K Xu D . Tangeretin alleviates sepsis-induced acute lung injury by inhibiting ferroptosis of macrophage via nrf2 signaling pathway. Chin Med. (2025) 20:11. doi: 10.1186/s13020-025-01063-8, PMID: 39815349 PMC11734455

[122] Zhou X Yang Y Qiu X Deng H Cao H Liao T . Antioxidant taurine inhibits chondrocyte ferroptosis through upregulation of ogt/gpx4 signaling in osteoarthritis induced by anterior cruciate ligament transection. J Adv Res. (2025) 77:551–67. doi: 10.1016/j.jare.2025.01.010, PMID: 39778769 PMC12627399

[123] Feng H Stockwell BR . Unsolved mysteries: how does lipid peroxidation cause ferroptosis? PloS Biol. (2018) 16:e2006203. doi: 10.1371/journal.pbio.2006203, PMID: 29795546 PMC5991413

[124] Luo S Zeng Y Chen B Yan J Ma F Zhuang G . Vitamin E and gpx4 cooperatively protect treg cells from ferroptosis and alleviate intestinal inflammatory damage in necrotizing enterocolitis. Redox Biol. (2024) 75:103303. doi: 10.1016/j.redox.2024.103303, PMID: 39137584 PMC11372871

[125] Wang J Liu Y Wang Y Sun L . The cross-link between ferroptosis and kidney diseases. Oxid Med Cell Longev. (2021) 2021:6654887. doi: 10.1155/2021/6654887, PMID: 34007403 PMC8110383

[126] Li Y Zhou Y Liu D Wang Z Qiu J Zhang J . Glutathione peroxidase 3 induced mitochondria-mediated apoptosis via ampk /erk1/2 pathway and resisted autophagy-related ferroptosis via ampk/mtor pathway in hyperplastic prostate. J Trans Med. (2023) 21:575. doi: 10.1186/s12967-023-04432-9, PMID: 37633909 PMC10463608

[127] Kerr JF . Shrinkage necrosis: A distinct mode of cellular death. J Pathol. (1971) 105:13–20. doi: 10.1002/path.1711050103, PMID: 4108566

[128] Liu S Yao S Yang H Liu S Wang Y . Autophagy: regulator of cell death. Cell Death Dis. (2023) 14:648. doi: 10.1038/s41419-023-06154-8, PMID: 37794028 PMC10551038

[129] Wang Z Wang X Dai X Xu T Qian X Chang M . 2d catalytic nanozyme enables cascade enzyodynamic effect-boosted and ca(2+) overload-induced synergistic ferroptosis/apoptosis in tumor. Adv Mater. (2024) 36:e2312316. doi: 10.1002/adma.202312316, PMID: 38501540

[v130] Li W Shi J Yu Z Garcia-Gabilondo M Held A Huang L . Slc22a17 as a cell death-linked regulator of tight junctions in cerebral ischemia. Stroke. (2024) 55:1650–9. doi: 10.1161/strokeaha.124.046736, PMID: 38738428

[131] Chen Z Liu B Zhou D Lei M Yang J Hu Z . Aqp4 regulates ferroptosis and oxidative stress of muller cells in diabetic retinopathy by regulating trpv4. Exp Cell Res. (2024) 439:114087. doi: 10.1016/j.yexcr.2024.114087, PMID: 38735619

[132] Qin Q Yu N Gu Y Ke W Zhang Q Liu X . Inhibiting multiple forms of cell death optimizes ganglion cells survival after retinal ischemia reperfusion injury. Cell Death Dis. (2022) 13:507. doi: 10.1038/s41419-022-04911-9, PMID: 35637215 PMC9151775

[133] Otsu W Ishida K Chinen N Nakamura S Shimazawa M Tsusaki H . Cigarette smoke extract and heated tobacco products promote ferritin cleavage and iron accumulation in human corneal epithelial cells. Sci Rep. (2021) 11:18555. doi: 10.1038/s41598-021-97956-3, PMID: 34535730 PMC8448754

[134] Newton K Strasser A Kayagaki N Dixit VM . Cell death. Cell. (2024) 187:235–56. doi: 10.1016/j.cell.2023.11.044, PMID: 38242081

[135] Rao Z Zhu Y Yang P Chen Z Xia Y Qiao C . Pyroptosis in inflammatory diseases and cancer. Theranostics. (2022) 12:4310–29. doi: 10.7150/thno.71086, PMID: 35673561 PMC9169370

[136] Zhou B Zhang JY Liu XS Chen HZ Ai YL Cheng K . Tom20 senses iron-activated ros signaling to promote melanoma cell pyroptosis. Cell Res. (2018) 28:1171–85. doi: 10.1038/s41422-018-0090-y, PMID: 30287942 PMC6274649

[137] Wang H Shu L Lv C Liu N Long Y Peng X . Brcc36 deubiquitinates hmgcr to regulate the interplay between ferroptosis and pyroptosis. Adv Sci (Weinh). (2024) 11:e2304263. doi: 10.1002/advs.202304263, PMID: 38178583 PMC10953584

[138] Huang Y Xu W Zhou R . Nlrp3 inflammasome activation and cell death. Cell Mol Immunol. (2021) 18:2114–27. doi: 10.1038/s41423-021-00740-6, PMID: 34321623 PMC8429580

[139] Meihe L Shan G Minchao K Xiaoling W Peng A Xili W . The ferroptosis-nlrp1 inflammasome: the vicious cycle of an adverse pregnancy. Front Cell Dev Biol. (2021) 9:707959. doi: 10.3389/fcell.2021.707959, PMID: 34490257 PMC8417576

[140] Zou M Ke Q Nie Q Qi R Zhu X Liu W . Inhibition of cgas-sting by jq1 alleviates oxidative stress-induced retina inflammation and degeneration. Cell Death Differ. (2022) 29:1816–33. doi: 10.1038/s41418-022-00967-4, PMID: 35347235 PMC9433402

[141] Shimada K Crother TR Karlin J Dagvadorj J Chiba N Chen S . Oxidized mitochondrial DNA activates the nlrp3 inflammasome during apoptosis. Immunity. (2012) 36:401–14. doi: 10.1016/j.immuni.2012.01.009, PMID: 22342844 PMC3312986

[142] Yan J Wan P Choksi S Liu ZG . Necroptosis and tumor progression. Trends Cancer. (2022) 8:21–7. doi: 10.1016/j.trecan.2021.09.003, PMID: 34627742 PMC8702466

[143] Tang R Xu J Zhang B Liu J Liang C Hua J . Ferroptosis, necroptosis, and pyroptosis in anticancer immunity. J Hematol Oncol. (2020) 13:110. doi: 10.1186/s13045-020-00946-7, PMID: 32778143 PMC7418434

[144] Tong X Tang R Xiao M Xu J Wang W Zhang B . Targeting cell death pathways for cancer therapy: recent developments in necroptosis, pyroptosis, ferroptosis, and cuproptosis research. J Hematol Oncol. (2022) 15:174. doi: 10.1186/s13045-022-01392-3, PMID: 36482419 PMC9733270

[145] Tong Y Wu Y Ma J Ikeda M Ide T Griffin CT . Comparative mechanistic study of rpe cell death induced by different oxidative stresses. Redox Biol. (2023) 65:102840. doi: 10.1016/j.redox.2023.102840, PMID: 37566944 PMC10440584

[146] Delehouzé C Comte A Leon-Icaza SA Cougoule C Hauteville M Goekjian P . Nigratine as dual inhibitor of necroptosis and ferroptosis regulated cell death. Sci Rep. (2022) 12:5118. doi: 10.1038/s41598-022-09019-w, PMID: 35332201 PMC8944179

[147] Ma R Li Y Dong X Zhang Y Chen X Zhang Y . Pax6/cxcl14 regulatory axis promotes the repair of corneal injury by enhancing corneal epithelial cell proliferation. J Trans Med. (2024) 22:458. doi: 10.1186/s12967-024-05270-z, PMID: 38750454 PMC11094923

[148] Sun R Zhang J Chen X Deng Y Gou J Yin T . An adaptive drug-releasing contact lens for personalized treatment of ocular infections and injuries. J Controlled Release. (2024) 369:114–27. doi: 10.1016/j.jconrel.2024.03.040, PMID: 38521167

[149] Liu Z Liu K Shi S Chen X Gu X Wang W . Alkali injury-induced pathological lymphangiogenesis in the iris facilitates the infiltration of T cells and ocular inflammation. JCI Insight. (2024) 9:e175479. doi: 10.1172/jci.insight.175479, PMID: 38587075 PMC11128208

[150] Sakai O Uchida T Imai H Ueta T . Glutathione peroxidase 4 plays an important role in oxidative homeostasis and wound repair in corneal epithelial cells. FEBS Open Bio. (2016) 6:1238–47. doi: 10.1002/2211-5463.12141, PMID: 28203523 PMC5302057

[151] Peng JJ Song WT Yao F Zhang X Peng J Luo XJ . Involvement of regulated necrosis in blinding diseases: focus on necroptosis and ferroptosis. Exp Eye Res. (2020) 191:107922. doi: 10.1016/j.exer.2020.107922, PMID: 31923413

[152] Liang N Song W Li J . Bpa promotes lung fibrosis in mice by regulating autophagy-dependent ferroptosis in alveolar epithelial cells. Ecotoxicol Environ Saf. (2024) 278:116412. doi: 10.1016/j.ecoenv.2024.116412, PMID: 38691879

[153] Sharma N Kaur M Agarwal T Sangwan VS Vajpayee RB . Treatment of acute ocular chemical burns. Surv Ophthalmol. (2018) 63:214–35. doi: 10.1016/j.survophthal.2017.09.005, PMID: 28935121

[154] Bizrah M Yusuf A Ahmad S . An update on chemical eye burns. Eye (Lond). (2019) 33:1362–77. doi: 10.1038/s41433-019-0456-5, PMID: 31086244 PMC7002428

[155] Witsberger EM Patel SV . Alkali burn over a lasik flap. Cornea. (2021) 40:907–9. doi: 10.1097/ico.0000000000002604, PMID: 33273190

[156] Zhou H Zhang W Bi M Wu J . The molecular mechanisms of action of ppar-Γ Agonists in the treatment of corneal alkali burns (Review). Int J Mol Med. (2016) 38:1003–11. doi: 10.3892/ijmm.2016.2699, PMID: 27499172 PMC5029963

[157] Welling JD Pike EC Mauger TF . Alkali burn of the ocular surface associated with a commonly used antifog agent for eyewear: two cases and a review of previous reports. Cornea. (2016) 35:289–91. doi: 10.1097/ico.0000000000000706, PMID: 26655480

[158] Liu P Wang W Li Z Li Y Yu X Tu J . Ferroptosis: A new regulatory mechanism in osteoporosis. Oxid Med Cell Longev. (2022) 2022:2634431. doi: 10.1155/2022/2634431, PMID: 35082963 PMC8786466

[159] Yang K Zeng L Yuan X Wang S Ge A Xu H . The mechanism of ferroptosis regulating oxidative stress in ischemic stroke and the regulation mechanism of natural pharmacological active components. BioMed Pharmacother. (2022) 154:113611. doi: 10.1016/j.biopha.2022.113611, PMID: 36081288

[160] Wang K Jiang L Zhong Y Zhang Y Yin Q Li S . Ferrostatin-1-loaded liposome for treatment of corneal alkali burn via targeting ferroptosis. Bioeng Transl Med. (2022) 7:e10276. doi: 10.1002/btm2.10276, PMID: 35600640 PMC9115688

[161] Wang Z Li H Zhou W Lee J Liu Z An Z . Ferrous sulfate-loaded hydrogel cures staphylococcus aureus infection via facilitating a ferroptosis-like bacterial cell death in a mouse keratitis model. Biomaterials. (2022) 290:121842. doi: 10.1016/j.biomaterials.2022.121842, PMID: 36206665

[162] Teng X Xiong X Sha X Lei Y Diao Y Liu J . Identification of hub genes and pathways of ferroptosis in fusarium keratitis by bioinformatics methods. Front Cell infection Microbiol. (2023) 13:1103471. doi: 10.3389/fcimb.2023.1103471, PMID: 36798084 PMC9927021

[163] Tavakoli A Flanagan JL . Dry eye disease: an (in)Convenient truth. Clin Exp Optom. (2022) 105:222–9. doi: 10.1080/08164622.2021.1945410, PMID: 34315353

[164] Karlen SJ Miller EB Burns ME . Microglia activation and inflammation during the death of mammalian photoreceptors. Annu Rev Vis Sci. (2020) 6:149–69. doi: 10.1146/annurev-vision-121219-081730, PMID: 32936734 PMC10135402

[165] Yumnamcha T Devi TS Singh LP . Auranofin mediates mitochondrial dysregulation and inflammatory cell death in human retinal pigment epithelial cells: implications of retinal neurodegenerative diseases. Front Neurosci. (2019) 13:1065. doi: 10.3389/fnins.2019.01065, PMID: 31649499 PMC6795687

[166] Datta S Cano M Ebrahimi K Wang L Handa JT . The impact of oxidative stress and inflammation on rpe degeneration in non-neovascular amd. Prog Retin Eye Res. (2017) 60:201–18. doi: 10.1016/j.preteyeres.2017.03.002, PMID: 28336424 PMC5600827

[167] Golberg L Martin LE Batchelor A . Biochemical changes in the tissues of animals injected with iron. 3. Lipid peroxidation. Biochem J. (1962) 83:291–8. doi: 10.1042/bj0830291, PMID: 13899654 PMC1243547

[168] Lee JJ Ishihara K Notomi S Efstathiou NE Ueta T Maidana D . Lysosome-associated membrane protein-2 deficiency increases the risk of reactive oxygen species-induced ferroptosis in retinal pigment epithelial cells. Biochem Biophys Res Commun. (2020) 521:414–9. doi: 10.1016/j.bbrc.2019.10.138, PMID: 31672277 PMC6935401

[169] Theriot CA Westby CM Morgan JLL Zwart SR Zanello SB . High dietary iron increases oxidative stress and radiosensitivity in the rat retina and vasculature after exposure to fractionated gamma radiation. NPJ Microgravity. (2016) 2:16014. doi: 10.1038/npjmgrav.2016.14, PMID: 28725729 PMC5515516

[170] Martin PM Gnana-Prakasam JP Roon P Smith RG Smith SB Ganapathy V . Expression and polarized localization of the hemochromatosis gene product hfe in retinal pigment epithelium. Invest Ophthalmol Vis Sci. (2006) 47:4238–44. doi: 10.1167/iovs.06-0026, PMID: 17003411

[171] Gnana-Prakasam JP Veeranan-Karmegam R Coothankandaswamy V Reddy SK Martin PM Thangaraju M . Loss of hfe leads to progression of tumor phenotype in primary retinal pigment epithelial cells. Invest Ophthalmol Vis Sci. (2013) 54:63–71. doi: 10.1167/iovs.12-10312, PMID: 23169885 PMC3544423

[172] Sterling J Guttha S Song Y Song D Hadziahmetovic M Dunaief JL . Iron importers zip8 and zip14 are expressed in retina and regulated by retinal iron levels. Exp Eye Res. (2017) 155:15–23. doi: 10.1016/j.exer.2016.12.008, PMID: 28057442 PMC5359041

[173] Vogt AS Arsiwala T Mohsen M Vogel M Manolova V Bachmann MF . On iron metabolism and its regulation. Int J Mol Sci. (2021) 22:4591. doi: 10.3390/ijms22094591, PMID: 33925597 PMC8123811

[174] Wysokinski D Zaras M Dorecka M Waszczyk M Szaflik J Blasiak J . An association between environmental factors and the ivs4+44c>a polymorphism of the dmt1 gene in age-related macular degeneration. Graefes Arch Clin Exp Ophthalmol. (2012) 250:1057–65. doi: 10.1007/s00417-012-1966-z, PMID: 22371024 PMC3382657

[175] Lee JJ Chang-Chien GP Lin S Hsiao YT Ke MC Chen A . 5-lipoxygenase inhibition protects retinal pigment epithelium from sodium iodate-induced ferroptosis and prevents retinal degeneration. Oxid Med Cell Longevity. (2022) 2022:1792894. doi: 10.1155/2022/1792894, PMID: 35251467 PMC8890867

[176] Hernández-Zimbrón LF Zamora-Alvarado R Ochoa-De la Paz L Velez-Montoya R Zenteno E Gulias-Cañizo R . Age-related macular degeneration: new paradigms for treatment and management of amd. Oxid Med Cell Longev. (2018) 2018:8374647. doi: 10.1155/2018/8374647, PMID: 29484106 PMC5816845

[177] Sun Y Zheng Y Wang C Liu Y . Glutathione depletion induces ferroptosis, autophagy, and premature cell senescence in retinal pigment epithelial cells. Cell Death Dis. (2018) 9:753. doi: 10.1038/s41419-018-0794-4, PMID: 29988039 PMC6037763

[178] Lei G Zhuang L Gan B . Targeting ferroptosis as a vulnerability in cancer. Nat Rev Cancer. (2022) 22:381–96. doi: 10.1038/s41568-022-00459-0, PMID: 35338310 PMC10243716

[179] Wei TT Zhang MY Zheng XH Xie TH Wang W Zou J . Interferon-Γ Induces retinal pigment epithelial cell ferroptosis by a jak1-2/stat1/slc7a11 signaling pathway in age-related macular degeneration. FEBS J. (2022) 289:1968–83. doi: 10.1111/febs.16272, PMID: 34741776

[180] Liu H Tang J Du Y Saadane A Samuels I Veenstra A . Transducin1, phototransduction and the development of early diabetic retinopathy. Invest Ophthalmol Vis Sci. (2019) 60:1538–46. doi: 10.1167/iovs.18-26433, PMID: 30994864 PMC6736377

[181] Ola MS Alhomida AS LaNoue KF . Gabapentin attenuates oxidative stress and apoptosis in the diabetic rat retina. Neurotox Res. (2019) 36:81–90. doi: 10.1007/s12640-019-00018-w, PMID: 30830678

[182] Zhang YH Wang DW Xu SF Zhang S Fan YG Yang YY . α-lipoic acid improves abnormal behavior by mitigation of oxidative stress, inflammation, ferroptosis, and tauopathy in P301s tau transgenic mice. Redox Biol. (2018) 14:535–48. doi: 10.1016/j.redox.2017.11.001, PMID: 29126071 PMC5684493

[183] Do MT Yau KW . Intrinsically photosensitive retinal ganglion cells. Physiol Rev. (2010) 90:1547–81. doi: 10.1152/physrev.00013.2010, PMID: 20959623 PMC4374737

[184] Parisi V Oddone F Ziccardi L Roberti G Coppola G Manni G . Citicoline and retinal ganglion cells: effects on morphology and function. Curr Neuropharmacol. (2018) 16:919–32. doi: 10.2174/1570159x15666170703111729, PMID: 28676014 PMC6120106

[185] Luo X Wang Y Zhu X Chen Y Xu B Bai X . Mcl attenuates atherosclerosis by suppressing macrophage ferroptosis via targeting keap1/nrf2 interaction. Redox Biol. (2024) 69:102987. doi: 10.1016/j.redox.2023.102987, PMID: 38100883 PMC10761782

[186] Shapouri-Moghaddam A Mohammadian S Vazini H Taghadosi M Esmaeili SA Mardani F . Macrophage plasticity, polarization, and function in health and disease. J Cell Physiol. (2018) 233:6425–40. doi: 10.1002/jcp.26429, PMID: 29319160 PMC13482560

[187] Yang Y Wang Y Guo L Gao W Tang TL Yan M . Interaction between macrophages and ferroptosis. Cell Death Dis. (2022) 13:355. doi: 10.1038/s41419-022-04775-z, PMID: 35429990 PMC9013379

[188] Ma J Zhang H Chen Y Liu X Tian J Shen W . The role of macrophage iron overload and ferroptosis in atherosclerosis. Biomolecules. (2022) 12:1702. doi: 10.3390/biom12111702, PMID: 36421722 PMC9688033

[189] Xu S Min J Wang F . Ferroptosis: an emerging player in immune cells. Sci Bull (Beijing). (2021) 66:2257–60. doi: 10.1016/j.scib.2021.02.026, PMID: 36654451

[190] Sheng S Zhang Y Jin L Sun W Zhu D Mei L . A ferritin-targeted biohybrid triggering ferroptosis immunotherapy via activating endogenous iron and replenishing exogenous iron simultaneously. Nat Commun. (2025) 16:6045. doi: 10.1038/s41467-025-61419-4, PMID: 40593723 PMC12217129

[191] Yang M Chen X Hu X Li H Huang H Fang Y . The nf-κb-slc7a11 axis regulates ferroptosis sensitivity in inflammatory macrophages. Cell Insight. (2025) 4:100257. doi: 10.1016/j.cellin.2025.100257, PMID: 40677785 PMC12268560

[192] Xiao H Du X Tao Z Jing N Bao S Gao WQ . Taurine inhibits ferroptosis mediated by the crosstalk between tumor cells and tumor-associated macrophages in prostate cancer. Adv Sci (Weinh). (2024) 11:e2303894. doi: 10.1002/advs.202303894, PMID: 38031260 PMC10797466

[193] Lu J Lu F Peng Z Zhang Z Jiang W Meng X . Clodronate liposome-mediated macrophage depletion ameliorates iron overload-induced dry eye disease. Exp Eye Res. (2025) 251:110204. doi: 10.1016/j.exer.2024.110204, PMID: 39662663

[194] Caligiuri MA . Human natural killer cells. Blood. (2008) 112:461–9. doi: 10.1182/blood-2007-09-077438, PMID: 18650461 PMC2481557

[195] Valipour B Velaei K Abedelahi A Karimipour M Darabi M Charoudeh HN . Nk cells: an attractive candidate for cancer therapy. J Cell Physiol. (2019) 234:19352–65. doi: 10.1002/jcp.28657, PMID: 30993712

[196] Guillerey C . Nk cells in the tumor microenvironment. Adv Exp Med Biol. (2020) 1273:69–90. doi: 10.1007/978-3-030-49270-0_4, PMID: 33119876

[197] Baker DJ Childs BG Durik M Wijers ME Sieben CJ Zhong J . Naturally occurring P16(Ink4a)-positive cells shorten healthy lifespan. Nature. (2016) 530:184–9. doi: 10.1038/nature16932, PMID: 26840489 PMC4845101

[198] Lei G Zhuang L Gan B . The roles of ferroptosis in cancer: tumor suppression, tumor microenvironment, and therapeutic interventions. Cancer Cell. (2024) 42:513–34. doi: 10.1016/j.ccell.2024.03.011, PMID: 38593779

[199] Kim R Taylor D Vonderheide RH Gabrilovich DI . Ferroptosis of immune cells in the tumor microenvironment. Trends Pharmacol Sci. (2023) 44:542–52. doi: 10.1016/j.tips.2023.06.005, PMID: 37380530

[200] Apte RS . Reducing treatment burden in amd. Cell. (2020) 180:1033. doi: 10.1016/j.cell.2020.02.028, PMID: 32200797 PMC7460805

[201] Dong X Song Y Liu Y Kou X Yang T Shi SX . Natural killer cells promote neutrophil extracellular traps and restrain macular degeneration in mice. Sci Transl Med. (2024) 16:eadi6626. doi: 10.1126/scitranslmed.adi6626, PMID: 39141700

[202] Freud AG Mundy-Bosse BL Yu J Caligiuri MA . The broad spectrum of human natural killer cell diversity. Immunity. (2017) 47:820–33. doi: 10.1016/j.immuni.2017.10.008, PMID: 29166586 PMC5728700

[203] Lanzolla G Marinò M Menconi F . Graves disease: latest understanding of pathogenesis and treatment options. Nat Rev Endocrinol. (2024) 20:647–60. doi: 10.1038/s41574-024-01016-5, PMID: 39039206

[204] Kulbay M Tanya SM Tuli N Dahoud J Dahoud A Alsaleh F . A comprehensive review of thyroid eye disease pathogenesis: from immune dysregulations to novel diagnostic and therapeutic approaches. Int J Mol Sci. (2024) 25:11628. doi: 10.3390/ijms252111628, PMID: 39519180 PMC11546489

[205] Chapman NM Boothby MR Chi H . Metabolic coordination of T cell quiescence and activation. Nat Rev Immunol. (2020) 20:55–70. doi: 10.1038/s41577-019-0203-y, PMID: 31406325

[206] Wik JA Skålhegg BS . T cell metabolism in infection. Front Immunol. (2022) 13:840610. doi: 10.3389/fimmu.2022.840610, PMID: 35359994 PMC8964062

[207] Wang W Green M Choi JE Gijón M Kennedy PD Johnson JK . Cd8(+) T cells regulate tumour ferroptosis during cancer immunotherapy. Nature. (2019) 569:270–4. doi: 10.1038/s41586-019-1170-y, PMID: 31043744 PMC6533917

[208] Xiang B Zhang M Li K Zhang Z Liu Y Gao M . The epitranscriptional factor pcif1 orchestrates cd8(+) T cell ferroptosis and activation to control antitumor immunity. Nat Immunol. (2025) 26:252–64. doi: 10.1038/s41590-024-02047-w, PMID: 39762445

[209] Bell HN Stockwell BR Zou W . Ironing out the role of ferroptosis in immunity. Immunity. (2024) 57:941–56. doi: 10.1016/j.immuni.2024.03.019, PMID: 38749397 PMC11101142

[210] Loi JK Alexandre YO Senthil K Schienstock D Sandford S Devi S . Corneal tissue-resident memory T cells form a unique immune compartment at the ocular surface. Cell Rep. (2022) 39:110852. doi: 10.1016/j.celrep.2022.110852, PMID: 35613584

[211] Bao X Zhong Y Yang C Chen Y Han Y Lin X . T-cell repertoire analysis in the conjunctiva of murine dry eye model. Invest Ophthalmol Vis Sci. (2023) 64:14. doi: 10.1167/iovs.64.3.14, PMID: 36877515 PMC10007900

[212] Peng X Li H Zhu L Zhao S Li Z Li S . Single-cell sequencing of the retina shows that ldha regulates pathogenesis of autoimmune uveitis. J Autoimmun. (2024) 143:103160. doi: 10.1016/j.jaut.2023.103160, PMID: 38160538

[213] Woo KM Mahrous MA D'Amico DJ Kiss S Kovacs KD . Prevalence of age-related macular degeneration in patients with chronic exposure to P2x7r inhibitors. Graefes Arch Clin Exp Ophthalmol. (2024) 262:3493–9. doi: 10.1007/s00417-024-06507-9, PMID: 38761206

[214] Sendecki A Ledwoń D Nycz J Wąsowska A Boguszewska-Chachulska A Mitas AW . A deep learning approach to explore the association of age-related macular degeneration polygenic risk score with retinal optical coherence tomography: A preliminary study. Acta Ophthalmol. (2024) 102:e1029–e39. doi: 10.1111/aos.16710, PMID: 38761033

[215] GBD 2019 Blindness and Vision Impairment Collaborators, Vision Loss Expert Group of the Global Burden of Disease Study . Trends in Prevalence of Blindness and Distance and near Vision Impairment over 30 Years: An Analysis for the Global Burden of Disease Study. Lancet Glob Health. (2021) 9:e130–e43. doi: 10.1016/s2214-109x(20)30425-3, PMID: 33275950 PMC7820390

[216] Wong WL Su X Li X Cheung CM Klein R Cheng CY . Global prevalence of age-related macular degeneration and disease burden projection for 2020 and 2040: A systematic review and meta-analysis. Lancet Glob Health. (2014) 2:e106–16. doi: 10.1016/s2214-109x(13)70145-1, PMID: 25104651

[217] Zarubina AV Neely DC Clark ME Huisingh CE Samuels BC Zhang Y . Prevalence of subretinal drusenoid deposits in older persons with and without age-related macular degeneration, by multimodal imaging. Ophthalmology. (2016) 123:1090–100. doi: 10.1016/j.ophtha.2015.12.034, PMID: 26875000 PMC4842107

[218] Cabral De Guimaraes TA Daich Varela M Georgiou M Michaelides M . Treatments for dry age-related macular degeneration: therapeutic avenues, clinical trials and future directions. Br J Ophthalmol. (2022) 106:297–304. doi: 10.1136/bjophthalmol-2020-318452, PMID: 33741584 PMC8867261

[219] Fleckenstein M Schmitz-Valckenberg S Chakravarthy U . Age-related macular degeneration: A review. JAMA. (2024) 331:147–57. doi: 10.1001/jama.2023.26074, PMID: 38193957 PMC12935482

[220] Adler R Curcio C Hicks D Price D Wong F . Cell death in age-related macular degeneration. Mol Vis. (1999) 5:31., PMID: 10562655

[221] Chen C Chen J Wang Y Liu Z Wu Y . Ferroptosis drives photoreceptor degeneration in mice with defects in all-trans-retinal clearance. J Biol Chem. (2021) 296:100187. doi: 10.1074/jbc.RA120.015779, PMID: 33334878 PMC7948481

[222] Xiong L Liu Y Wang Y Zhao H Song X Fan W . The protective effect of lonicera japonica thunb. Against lipopolysaccharide-induced acute lung injury in mice: modulation of inflammation, oxidative stress, and ferroptosis. J Ethnopharmacol. (2024) 331:118333. doi: 10.1016/j.jep.2024.118333, PMID: 38750986

[223] Zhao X Gao M Liang J Chen Y Wang Y Wang Y . Slc7a11 reduces laser-induced choroidal neovascularization by inhibiting rpe ferroptosis and vegf production. Front Cell Dev Biol. (2021) 9:639851. doi: 10.3389/fcell.2021.639851, PMID: 33681224 PMC7930391

[224] Martis RM Knight LJ Acosta ML Black J Ng R Ji LCL . Early onset of age-related changes in the retina of cystine/glutamate antiporter knockout mice. Exp Eye Res. (2023) 227:109364. doi: 10.1016/j.exer.2022.109364, PMID: 36586548

[225] Biesemeier A Yoeruek E Eibl O Schraermeyer U . Iron accumulation in bruch's membrane and melanosomes of donor eyes with age-related macular degeneration. Exp Eye Res. (2015) 137:39–49. doi: 10.1016/j.exer.2015.05.019, PMID: 26026877

[226] Ashok A Chaudhary S Wise AS Rana NA McDonald D Kritikos AE . Release of Iron-Loaded Ferritin in Sodium Iodate-Induced Model of Age Related Macular Degeneration: An in-Vitro and in-Vivo Study. Antioxid (Basel). (2021) 10:1253. doi: 10.3390/antiox10081253, PMID: 34439501 PMC8389213

[227] Hahn P Ying GS Beard J Dunaief JL . Iron levels in human retina: sex difference and increase with age. Neuroreport. (2006) 17:1803–6. doi: 10.1097/WNR.0b013e3280107776, PMID: 17164668

[228] Nag TC Kumar P Wadhwa S . Age related distribution of 4-hydroxy 2-nonenal immunoreactivity in human retina. Exp Eye Res. (2017) 165:125–35. doi: 10.1016/j.exer.2017.09.014, PMID: 28986146

[229] Hollyfield JG Bonilha VL Rayborn ME Yang X Shadrach KG Lu L . Oxidative damage-induced inflammation initiates age-related macular degeneration. Nat Med. (2008) 14:194–8. doi: 10.1038/nm1709, PMID: 18223656 PMC2748836

[230] Song D Song Y Hadziahmetovic M Zhong Y Dunaief JL . Systemic administration of the iron chelator deferiprone protects against light-induced photoreceptor degeneration in the mouse retina. Free Radic Biol Med. (2012) 53:64–71. doi: 10.1016/j.freeradbiomed.2012.04.020, PMID: 22579919 PMC3380452

[231] Song D Zhao L Li Y Hadziahmetovic M Song Y Connelly J . The oral iron chelator deferiprone protects against systemic iron overload-induced retinal degeneration in hepcidin knockout mice. Invest Ophthalmol Vis Sci. (2014) 55:4525–32. doi: 10.1167/iovs.14-14568, PMID: 24970260 PMC4106252

[232] Ueta T Inoue T Furukawa T Tamaki Y Nakagawa Y Imai H . Glutathione peroxidase 4 is required for maturation of photoreceptor cells. J Biol Chem. (2012) 287:7675–82. doi: 10.1074/jbc.M111.335174, PMID: 22207760 PMC3293550

[233] Feher J Kovacs I Artico M Cavallotti C Papale A Balacco Gabrieli C . Mitochondrial alterations of retinal pigment epithelium in age-related macular degeneration. Neurobiol Aging. (2006) 27:983–93. doi: 10.1016/j.neurobiolaging.2005.05.012, PMID: 15979212

[234] Karunadharma PP Nordgaard CL Olsen TW Ferrington DA . Mitochondrial DNA damage as a potential mechanism for age-related macular degeneration. Invest Ophthalmol Visual Sci. (2010) 51:5470–9. doi: 10.1167/iovs.10-5429, PMID: 20505194 PMC3061495

[235] Guo J Zhou Y Liu D Wang M Wu Y Tang D . Mitochondria as multifaceted regulators of ferroptosis. Life Metab. (2022) 1:134–48. doi: 10.1093/lifemeta/loac035, PMID: 39872359 PMC11749789

[236] Terluk MR Kapphahn RJ Soukup LM Gong H Gallardo C Montezuma SR . Investigating mitochondria as a target for treating age-related macular degeneration. J Neurosci. (2015) 35:7304–11. doi: 10.1523/jneurosci.0190-15.2015, PMID: 25948278 PMC4420790

[237] Wang S Liu Y Liu Y Li C Wan Q Yang L . Reversed senescence of retinal pigment epithelial cell by coculture with embryonic stem cell via the tgfβ and pi3k pathways. Front Cell Dev Biol. (2020) 8:588050. doi: 10.3389/fcell.2020.588050, PMID: 33324644 PMC7726211

[238] Kim MH Kwon SY Woo SY Seo WD Kim DY . Antioxidative effects of chrysoeriol via activation of the nrf2 signaling pathway and modulation of mitochondrial function. Molecules. (2021) 26:313. doi: 10.3390/molecules26020313, PMID: 33435366 PMC7826659

[239] Tan W Zou J Yoshida S Jiang B Zhou Y . The role of inflammation in age-related macular degeneration. Int J Biol Sci. (2020) 16:2989–3001. doi: 10.7150/ijbs.49890, PMID: 33061811 PMC7545698

[240] Ananth S Gnana-Prakasam JP Bhutia YD Veeranan-Karmegam R Martin PM Smith SB . Regulation of the cholesterol efflux transporters abca1 and abcg1 in retina in hemochromatosis and by the endogenous siderophore 2,5-dihydroxybenzoic acid. Biochim Biophys Acta. (2014) 1842:603–12. doi: 10.1016/j.bbadis.2014.01.010, PMID: 24462739 PMC4289134

[241] Gupta U Ghosh S Wallace CT Shang P Xin Y Nair AP . Increased lcn2 (Lipocalin 2) in the rpe decreases autophagy and activates inflammasome-ferroptosis processes in a mouse model of dry amd. Autophagy. (2023) 19:92–111. doi: 10.1080/15548627.2022.2062887, PMID: 35473441 PMC9809950

[242] Li Y Song D Song Y Zhao L Wolkow N Tobias JW . Iron-induced local complement component 3 (C3) up-regulation via non-canonical transforming growth factor (Tgf)-β Signaling in the retinal pigment epithelium. J Biol Chem. (2015) 290:11918–34. doi: 10.1074/jbc.M115.645903, PMID: 25802332 PMC4424331

[243] Song D Kanu LN Li Y Kelly KL Bhuyan RK Aleman T . Amd-like retinopathy associated with intravenous iron. Exp Eye Res. (2016) 151:122–33. doi: 10.1016/j.exer.2016.08.008, PMID: 27565570 PMC5045814

[244] Liu B Wang W Shah A Yu M Liu Y He L . Sodium iodate induces ferroptosis in human retinal pigment epithelium arpe-19 cells. Cell Death Dis. (2021) 12:230. doi: 10.1038/s41419-021-03520-2, PMID: 33658488 PMC7930128

[245] Tang Z Ju Y Dai X Ni N Liu Y Zhang D . Ho-1-mediated ferroptosis as a target for protection against retinal pigment epithelium degeneration. Redox Biol. (2021) 43:101971. doi: 10.1016/j.redox.2021.101971, PMID: 33895485 PMC8099560

[246] Wei H Chen C Di F Sun C Wang X Sun M . Pm(2.5)-induced ferroptosis by nrf2/hmox1 signaling pathway led to inflammation in microglia. Environ pollut. (2024) 352:124130. doi: 10.1016/j.envpol.2024.124130, PMID: 38729511

[247] Yang Y Wang Y Deng Y Lu J Xiao L Li J . Fructus lycii and salvia miltiorrhiza bunge extract attenuate oxidative stress-induced photoreceptor ferroptosis in retinitis pigmentosa. BioMed Pharmacother. (2023) 167:115547. doi: 10.1016/j.biopha.2023.115547, PMID: 37741257

[248] Sohns C Zabel M . Current role of amiodarone in antiarrhythmic therapy. Herzschrittmachertherapie Elektrophysiologie. (2010) 21:239–43. doi: 10.1007/s00399-010-0091-0, PMID: 21104260

[249] Chen Y Okano K Maeda T Chauhan V Golczak M Maeda A . Mechanism of all-trans-retinal toxicity with implications for stargardt disease and age-related macular degeneration. J Biol Chem. (2012) 287:5059–69. doi: 10.1074/jbc.M111.315432, PMID: 22184108 PMC3281612

[250] Chen Z Zhu X Lu MM Ou Q Wang X Zhao Z . Phospho1 suppresses ferroptosis in retinal pigment epithelial cells by reducing the levels of phosphatidylethanolamine molecular species. Adv Sci (Weinh). (2025) 12:e2505359. doi: 10.1002/advs.202505359, PMID: 40396905 PMC12302541

[251] Su W Gao Y Jia X Chen X Wu J Wen Y . Single-cell transcriptome atlas of spontaneous dry age-related macular degeneration in macaques. Fundam Res. (2025) 5:1034–46. doi: 10.1016/j.fmre.2023.02.028, PMID: 40528953 PMC12167874

[252] Chaudhary K Promsote W Ananth S Veeranan-Karmegam R Tawfik A Arjunan P . Iron overload accelerates the progression of diabetic retinopathy in association with increased retinal renin expression. Sci Rep. (2018) 8:3025. doi: 10.1038/s41598-018-21276-2, PMID: 29445185 PMC5813018

[253] López IM Díez A Velilla S Rueda A Alvarez A Pastor CJ . Prevalence of diabetic retinopathy and eye care in a rural area of Spain. Ophthalmic Epidemiol. (2002) 9:205–14. doi: 10.1076/opep.9.3.205.1516, PMID: 12045887

[254] Gerhardinger C Costa MB Coulombe MC Toth I Hoehn T Grosu P . Expression of acute-phase response proteins in retinal müller cells in diabetes. Invest Ophthalmol Vis Sci. (2005) 46:349–57. doi: 10.1167/iovs.04-0860, PMID: 15623795

[255] Konerirajapuram NS Coral K Punitham R Sharma T Kasinathan N Sivaramakrishnan R . Trace elements iron, copper and zinc in vitreous of patients with various vitreoretinal diseases. Indian J Ophthalmol. (2004) 52:145–8. 15283220

[256] Zhang D Lv FL Wang GH . Effects of hif-1α on diabetic retinopathy angiogenesis and vegf expression. Eur Rev Med Pharmacol Sci. (2018) 22:5071–6. doi: 10.26355/eurrev_201808_15699, PMID: 30178824

[257] Yang X Huo F Liu B Liu J Chen T Li J . Crocin inhibits oxidative stress and pro-inflammatory response of microglial cells associated with diabetic retinopathy through the activation of pi3k/akt signaling pathway. J Mol Neurosci. (2017) 61:581–9. doi: 10.1007/s12031-017-0899-8, PMID: 28238066

[258] Peters J . Cytosolic (Pro)Renin and the matter of intracellular renin actions. Front Biosci (Schol Ed). (2013) 5:198–205. doi: 10.2741/s366, PMID: 23277045

[259] Abcouwer SF . Angiogenic factors and cytokines in diabetic retinopathy. J Clin Cell Immunol. (2013) 1:1–12. doi: 10.4172/2155-9899, PMID: 24319628 PMC3852182

[260] Saxena R Madhu SV Shukla R Prabhu KM Gambhir JK . Postprandial hypertriglyceridemia and oxidative stress in patients of type 2 diabetes mellitus with macrovascular complications. Clinica Chimica Acta; Int J Clin Chem. (2005) 359:101–8. doi: 10.1016/j.cccn.2005.03.036, PMID: 15893742

[261] Bone RN Oyebamiji O Talware S Selvaraj S Krishnan P Syed F . A computational approach for defining a signature of β-cell golgi stress in diabetes. Diabetes. (2020) 69:2364–76. doi: 10.2337/db20-0636, PMID: 32820009 PMC7576569

[262] Zhang J Qiu Q Wang H Chen C Luo D . Trim46 contributes to high glucose-induced ferroptosis and cell growth inhibition in human retinal capillary endothelial cells by facilitating gpx4 ubiquitination. Exp Cell Res. (2021) 407:112800. doi: 10.1016/j.yexcr.2021.112800, PMID: 34487731

[263] Mu L Wang D Dong Z Wu J Wu X Su J . Abnormal levels of serum ferroptosis-related biomarkers in diabetic retinopathy. J Ophthalmol. (2022) 2022:3353740. doi: 10.1155/2022/3353740, PMID: 36620526 PMC9822742

[264] Yang J Liu Z . Mechanistic pathogenesis of endothelial dysfunction in diabetic nephropathy and retinopathy. Front Endocrinol (Lausanne). (2022) 13:816400. doi: 10.3389/fendo.2022.816400, PMID: 35692405 PMC9174994

[265] Fan X Xu M Ren Q Fan Y Liu B Chen J . Downregulation of fatty acid binding protein 4 alleviates lipid peroxidation and oxidative stress in diabetic retinopathy by regulating peroxisome proliferator-activated receptor Γ-mediated ferroptosis. Bioengineered. (2022) 13:10540–51. doi: 10.1080/21655979.2022.2062533, PMID: 35441580 PMC9161966

[266] Cheloni R Gandolfi SA Signorelli C Odone A . Global prevalence of diabetic retinopathy: protocol for a systematic review and meta-analysis. BMJ Open. (2019) 9:e022188. doi: 10.1136/bmjopen-2018-022188, PMID: 30833309 PMC6443069

[267] Oskarsson ME Paulsson JF Schultz SW Ingelsson M Westermark P Westermark GT . *In vivo* seeding and cross-seeding of localized amyloidosis: A molecular link between type 2 diabetes and alzheimer disease. Am J Pathol. (2015) 185:834–46. doi: 10.1016/j.ajpath.2014.11.016, PMID: 25700985

[268] Zhang K Wang T Sun GF Xiao JX Jiang LP Tou FF . Metformin Protects against Retinal Ischemia/Reperfusion Injury through Ampk-Mediated Mitochondrial Fusion. Free Radic Biol Med. (2023) 205:47–61. doi: 10.1016/j.freeradbiomed.2023.05.019, PMID: 37253410

[269] Chandrasekaran PR Madanagopalan VG . Role of curcumin in retinal diseases-a review. Graefes Arch Clin Exp Ophthalmol. (2022) 260:1457–73. doi: 10.1007/s00417-021-05542-0, PMID: 35015114 PMC8748528

[270] Mathew B Ravindran S Liu X Torres L Chennakesavalu M Huang CC . Mesenchymal stem cell-derived extracellular vesicles and retinal ischemia-reperfusion. Biomaterials. (2019) 197:146–60. doi: 10.1016/j.biomaterials.2019.01.016, PMID: 30654160 PMC6425741

[271] Rahimi M Leahy S Matei N Burford J Blair NP Shahidi M . Impairments of retinal hemodynamics and oxygen metrics in ocular hypertension-induced ischemia-reperfusion. Exp Eye Res. (2022) 225:109278. doi: 10.1016/j.exer.2022.109278, PMID: 36252653 PMC10985794

[272] Lin LT Chen JT Tai MC Chen YH Chen CL Pao SI . Protective effects of hypercapnic acidosis on ischemia-reperfusion-induced retinal injury. PloS One. (2019) 14:e0211185. doi: 10.1371/journal.pone.0211185, PMID: 30682118 PMC6347245

[273] Abcouwer SF Shanmugam S Muthusamy A Lin CM Kong D Hager H . Inflammatory resolution and vascular barrier restoration after retinal ischemia reperfusion injury. J Neuroinflamm. (2021) 18:186. doi: 10.1186/s12974-021-02237-5, PMID: 34446062 PMC8394696

[274] Wang X Li M Diao K Wang Y Chen H Zhao Z . Deferoxamine attenuates visual impairment in retinal ischemia–Reperfusion via inhibiting ferroptosis. Sci Rep. (2023) 13:20145. doi: 10.1038/s41598-023-46104-0, PMID: 37978208 PMC10656451

[275] Chen B Caballero S Seo S Grant MB Lewin AS . Delivery of antioxidant enzyme genes to protect against ischemia/reperfusion-induced injury to retinal microvasculature. Invest Ophthalmol Vis Sci. (2009) 50:5587–95. doi: 10.1167/iovs.09-3633, PMID: 19628743 PMC3756491

[276] Gehlbach P Purple RL . Enhancement of retinal recovery by conjugated deferoxamine after ischemia-reperfusion. Invest Ophthalmol Vis Sci. (1994) 35:669–76., PMID: 7509327

[277] Balla A Tran B Valtari A Steven P Scarpellini C Augustyns K . A novel ferroptosis inhibitor uamc-3203, a potential treatment for corneal epithelial wound. Pharmaceutics. (2022) 15:118. doi: 10.3390/pharmaceutics15010118, PMID: 36678747 PMC9863691

[278] Li Y Wen Y Liu X Li Z Lin B Deng C . Single-cell rna sequencing reveals a landscape and targeted treatment of ferroptosis in retinal ischemia/reperfusion injury. J Neuroinflamm. (2022) 19:261. doi: 10.1186/s12974-022-02621-9, PMID: 36289494 PMC9597965

[279] Lu Y Zhu F Zhou X Li Y Rong G Liu N . A supramolecular deferoxamine-crisaborole nanoparticle targets ferroptosis, inflammation, and oxidative stress in the treatment of retinal ischemia/reperfusion injury. Nano Lett. (2025) 25:1058–66. doi: 10.1021/acs.nanolett.4c05012, PMID: 39670541

[280] Ni Y Hu Y Zhu L Jiang X Zhang H Liu J . Lycium barbarum polysaccharide-derived nanoparticles protect visual function by inhibiting rgc ferroptosis and microglial activation in retinal ischemia–Reperfusion mice. Adv Healthc Mater. (2024) 13:e2304285. doi: 10.1002/adhm.202304285, PMID: 38994661

[281] Deleon E Lederman M Berenstein E Meir T Chevion M Chowers I . Alteration in iron metabolism during retinal degeneration in rd10 mouse. Invest Ophthalmol Vis Sci. (2009) 50:1360–5. doi: 10.1167/iovs.08-1856, PMID: 18997094

[282] Yefimova MG Jeanny JC Keller N Sergeant C Guillonneau X Beaumont C . Impaired retinal iron homeostasis associated with defective phagocytosis in royal college of surgeons rats. Invest Ophthalmol Vis Sci. (2002) 43:537–45., PMID: 11818402

[283] Obolensky A Berenshtein E Lederman M Bulvik B Alper-Pinus R Yaul R . Zinc-desferrioxamine attenuates retinal degeneration in the rd10 mouse model of retinitis pigmentosa. Free Radic Biol Med. (2011) 51:1482–91. doi: 10.1016/j.freeradbiomed.2011.07.014, PMID: 21824515

[284] Wang K Peng B Xiao J Weinreb O Youdim MBH Lin B . Iron-chelating drugs enhance cone photoreceptor survival in a mouse model of retinitis pigmentosa. Invest Ophthalmol Vis Sci. (2017) 58:5287–97. doi: 10.1167/iovs.17-22096, PMID: 29049732

[285] Rogers BS Symons RC Komeima K Shen J Xiao W Swaim ME . Differential sensitivity of cones to iron-mediated oxidative damage. Invest Ophthalmol Vis Sci. (2007) 48:438–45. doi: 10.1167/iovs.06-0528, PMID: 17197565

[286] Zeng Z Gao ZL Zhang ZP Jiang HB Yang CQ Yang J . Downregulation of cks1b restrains the proliferation, migration, invasion and angiogenesis of retinoblastoma cells through the mek/erk signaling pathway. Int J Mol Med. (2024) 54:58. doi: 10.3892/ijmm.2024.5382, PMID: 38757359 PMC11188980

[287] Ancona-Lezama D Dalvin LA Shields CL . Modern treatment of retinoblastoma: A 2020 review. Indian J Ophthalmol. (2020) 68:2356–65. doi: 10.4103/ijo.IJO_721_20, PMID: 33120616 PMC7774148

[288] Peeler CE Gonzalez E . Retinoblastoma. N Engl J Med. (2022) 386:2412. doi: 10.1056/NEJMicm2118356, PMID: 35731655

[289] Kuganesan N Dlamini S Tillekeratne LMV Taylor WR . Tumor suppressor P53 promotes ferroptosis in oxidative stress conditions independent of modulation of ferroptosis by P21, cdks, rb, and E2f. J Biol Chem. (2021) 297:101365. doi: 10.1016/j.jbc.2021.101365, PMID: 34728216 PMC8661017

[290] Liu K Huang J Liu J Klionsky DJ Kang R Tang D . Induction of autophagy-dependent ferroptosis to eliminate drug-tolerant human retinoblastoma cells. Cell Death Dis. (2022) 13:521. doi: 10.1038/s41419-022-04974-8, PMID: 35654783 PMC9163041

[291] He R Liu B Xiong R Geng B Meng H Lin W . Itaconate inhibits ferroptosis of macrophage via nrf2 pathways against sepsis-induced acute lung injury. Cell Death Discov. (2022) 8:43. doi: 10.1038/s41420-021-00807-3, PMID: 35110526 PMC8810876

[292] Liu H Gan Q Lai Y Pan Z Jin Q Li J . Usp14 increases the sensitivity of retinoblastoma to cisplatin by mediating the ferroptosis. Naunyn Schmiedebergs Arch Pharmacol. (2024) 397:8671–80. doi: 10.1007/s00210-024-03174-9, PMID: 38819674 PMC11522062

[293] Gong D Wu N Chen H Zhang W Yan C Zhang C . Phytic acid-loaded polyvinyl alcohol hydrogel promotes wound healing of injured corneal epithelium through inhibiting ferroptosis. Redox Biol. (2024) 76:103354. doi: 10.1016/j.redox.2024.103354, PMID: 39298836 PMC11426138

[294] Gomes JA Tan D Rapuano CJ Belin MW Ambrósio R Jr. Guell JL . Global consensus on keratoconus and ectatic diseases. Cornea. (2015) 34:359–69. doi: 10.1097/ico.0000000000000408, PMID: 25738235

[295] Jones-Jordan LA Walline JJ Sinnott LT Kymes SM Zadnik K . Asymmetry in keratoconus and vision-related quality of life. Cornea. (2013) 32:267–72. doi: 10.1097/ICO.0b013e31825697c4, PMID: 22825402 PMC3482277

[296] Cai Y Zhou T Cai X Shi W Sun H Fu Y . Deciphering mitochondrial dysfunction in keratoconus: insights into acsl4 from machine learning-based bulk and single-cell transcriptome analyses and experimental validation. Comput Struct Biotechnol J. (2025) 27:1962–74. doi: 10.1016/j.csbj.2025.05.013, PMID: 40496889 PMC12149552

[297] Wang H Song F Feng J Qi X Ma L Xie L . Tannin coordinated nanozyme composite-based hybrid hydrogel eye drops for prophylactic treatment of multidrug-resistant pseudomonas aeruginosa keratitis. J Nanobiotechnolo. (2022) 20:445. doi: 10.1186/s12951-022-01653-w, PMID: 36242070 PMC9563483

[298] Jadi PK Dave A Issa R Tabbasum K Okurowska K Samarth A . Tetraspanin cd9-derived peptides inhibit pseudomonas aeruginosa corneal infection and aid in wound healing of corneal epithelial cells. Ocul Surf. (2024) 32:211–8. doi: 10.1016/j.jtos.2023.07.001, PMID: 37406881

[299] Chen Q Wang L Wei Y Xu X Guo X Liang Q . Ferroptosis as a potential therapeutic target for reducing inflammation and corneal scarring in bacterial keratitis. Invest Ophthalmol Vis Sci. (2024) 65:29. doi: 10.1167/iovs.65.2.29, PMID: 38381413 PMC10893897

[300] Craig JP Nichols KK Akpek EK Caffery B Dua HS Joo CK . Tfos dews ii definition and classification report. Ocul Surf. (2017) 15:276–83. doi: 10.1016/j.jtos.2017.05.008, PMID: 28736335

[301] Jones L Downie LE Korb D Benitez-Del-Castillo JM Dana R Deng SX . Tfos dews ii management and therapy report. Ocul Surf. (2017) 15:575–628. doi: 10.1016/j.jtos.2017.05.006, PMID: 28736343

[302] Bron AJ De Paiva CS Chauhan SK Bonini S Gabison EE Jain S . Tfos dews ii pathophysiology report. Ocul Surf. (2017) 15:438–510. doi: 10.1016/j.jtos.2017.05.011, PMID: 28736340

[303] Mi Y Wei C Sun L Liu H Zhang J Luo J . Melatonin inhibits ferroptosis and delays age-related cataract by regulating sirt6/P-nrf2/gpx4 and sirt6/ncoa4/fth1 pathways. BioMed Pharmacother. (2023) 157:114048. doi: 10.1016/j.biopha.2022.114048, PMID: 36463827

[304] Zuo X Zeng H Wang B Yang X He D Wang L . Akr1c1 protects corneal epithelial cells against oxidative stress-mediated ferroptosis in dry eye. Invest Ophthalmol Vis Sci. (2022) 63:3. doi: 10.1167/iovs.63.10.3, PMID: 36066316 PMC9463717

[305] Cheng YH Huang HP Chen HH . Mucoadhesive phenylboronic acid-grafted carboxymethyl cellulose hydrogels containing glutathione for treatment of corneal epithelial cells exposed to benzalkonium chloride. Colloids Surf B Biointerfaces. (2024) 238:113884. doi: 10.1016/j.colsurfb.2024.113884, PMID: 38565006

[306] Zhang Y Zhou T Wang K Luo C Chen D Lv Z . Corneal mucin-targeting liposome nanoplatforms enable effective treatment of dry eye diseases by integrated regulation of ferroptosis and inflammation. Adv Sci (Weinh). (2025) 12:e2411172. doi: 10.1002/advs.202411172, PMID: 39605017 PMC11744570

[307] Wang B Zeng H Zhao X Zuo X Yang X Wang L . D609-polymer-based delivery strategy targeting ferroptosis in treatment of dry eye disease. Chem Eng J. (2025) 503:158050. doi: 10.1016/j.cej.2024.158050

[308] Jayaram H Kolko M Friedman DS Gazzard G . Glaucoma: now and beyond. Lancet. (2023) 402:1788–801. doi: 10.1016/s0140-6736(23)01289-8, PMID: 37742700

[309] Weinreb RN Aung T Medeiros FA . The pathophysiology and treatment of glaucoma: A review. JAMA. (2014) 311:1901–11. doi: 10.1001/jama.2014.3192, PMID: 24825645 PMC4523637

[310] Kang JM Tanna AP . Glaucoma. Med Clin North Am. (2021) 105:493–510. doi: 10.1016/j.mcna.2021.01.004, PMID: 33926643

[311] Youale J Bigot K Kodati B Jaworski T Fan Y Nsiah NY . Neuroprotective effects of transferrin in experimental glaucoma models. Int J Mol Sci. (2022) 23:12753. doi: 10.3390/ijms232112753, PMID: 36361544 PMC9659282

[312] Ramdas WD . The relation between dietary intake and glaucoma: A systematic review. Acta Ophthalmol. (2018) 96:550–6. doi: 10.1111/aos.13662, PMID: 29461678

[313] Stein JD Khawaja AP Weizer JS . Glaucoma in adults-screening, diagnosis, and management: A review. JAMA. (2021) 325:164–74. doi: 10.1001/jama.2020.21899, PMID: 33433580

[314] Yao F Peng J Zhang E Ji D Gao Z Tang Y . Pathologically high intraocular pressure disturbs normal iron homeostasis and leads to retinal ganglion cell ferroptosis in glaucoma. Cell Death Differ. (2023) 30:69–81. doi: 10.1038/s41418-022-01046-4, PMID: 35933500 PMC9883496

[315] Hoelzgen F Nguyen TTP Klukin E Boumaiza M Srivastava AK Kim EY . Structural basis for the intracellular regulation of ferritin degradation. Nat Commun. (2024) 15:3802. doi: 10.1038/s41467-024-48151-1, PMID: 38714719 PMC11076521

[316] Chen Y Khan RS Cwanger A Song Y Steenstra C Bang S . Dexras1, a small gtpase, is required for glutamate-nmda neurotoxicity. J Neurosci. (2013) 33:3582–7. doi: 10.1523/jneurosci.1497-12.2013, PMID: 23426685 PMC3711661

[317] Cheah JH Kim SF Hester LD Clancy KW Patterson SE 3rd Papadopoulos V . Nmda receptor-nitric oxide transmission mediates neuronal iron homeostasis via the gtpase dexras1. Neuron. (2006) 51:431–40. doi: 10.1016/j.neuron.2006.07.011, PMID: 16908409 PMC3150500

[318] Sakamoto K Suzuki T Takahashi K Koguchi T Hirayama T Mori A . Iron-chelating agents attenuate nmda-induced neuronal injury via reduction of oxidative stress in the rat retina. Exp Eye Res. (2018) 171:30–6. doi: 10.1016/j.exer.2018.03.008, PMID: 29530811

[319] Cui QN Bargoud AR Ross AG Song Y Dunaief JL . Oral administration of the iron chelator deferiprone protects against loss of retinal ganglion cells in a mouse model of glaucoma. Exp Eye Res. (2020) 193:107961. doi: 10.1016/j.exer.2020.107961, PMID: 32045598 PMC7584350

[320] Ashok A Singh N Chaudhary S Bellamkonda V Kritikos AE Wise AS . Retinal degeneration and alzheimer's disease: an evolving link. Int J Mol Sci. (2020) 21:7290. doi: 10.3390/ijms21197290, PMID: 33023198 PMC7582766

[321] Liu HT Zhang Q Jiang ZX Xu YX Wan QQ Tao LM . Efficacy and safety of high-dose ultrasound cyclo-plasty procedure in refractory glaucoma. Int J Ophthalmol. (2020) 13:1391–6. doi: 10.18240/ijo.2020.09.09, PMID: 32953577 PMC7459215

[322] Tang J Zhuo Y Li Y . Effects of iron and zinc on mitochondria: potential mechanisms of glaucomatous injury. Front Cell Dev Biol. (2021) 9:720288. doi: 10.3389/fcell.2021.720288, PMID: 34447755 PMC8383321

[323] Guo M Zhu Y Shi Y Meng X Dong X Zhang H . Inhibition of ferroptosis promotes retina ganglion cell survival in experimental optic neuropathies. Redox Biol. (2022) 58:102541. doi: 10.1016/j.redox.2022.102541, PMID: 36413918 PMC9679710

[324] Thompson J Lakhani N . Cataracts. Prim Care. (2015) 42:409–23. doi: 10.1016/j.pop.2015.05.012, PMID: 26319346

[325] Asbell PA Dualan I Mindel J Brocks D Ahmad M Epstein S . Age-related cataract. Lancet (London England). (2005) 365:599–609. doi: 10.1016/s0140-6736(05)17911-2, PMID: 15708105

[326] Bhuyan KC Master RW Coles RS Bhuyan DK . Molecular mechanisms of cataractogenesis: iv. Evidence of phospholipid. Malondialdehyde adduct in human senile cataract. Mech Ageing Dev. (1986) 34:289–96. doi: 10.1016/0047-6374(86)90080-1, PMID: 3724254

[327] Huang L Estrada R Yappert MC Borchman D . Oxidation-induced changes in human lens epithelial cells. 1. Phospholipids. Free Radic Biol Med. (2006) 41:1425–32. doi: 10.1016/j.freeradbiomed.2006.07.022, PMID: 17023269

[328] Reddy VN Giblin FJ Lin LR Dang L Unakar NJ Musch DC . Glutathione peroxidase-1 deficiency leads to increased nuclear light scattering, membrane damage, and cataract formation in gene-knockout mice. Invest Ophthalmol Vis Sci. (2001) 42:3247–55., PMID: 11726630

[329] Wei Z Hao C Huangfu J Srinivasagan R Zhang X Fan X . Aging lens epithelium is susceptible to ferroptosis. Free Radic Biol Med. (2021) 167:94–108. doi: 10.1016/j.freeradbiomed.2021.02.010, PMID: 33722625 PMC8096685

[330] Babizhayev MA . Failure to withstand oxidative stress induced by phospholipid hydroperoxides as a possible cause of the lens opacities in systemic diseases and ageing. Biochim Biophys Acta. (1996) 1315:87–99. doi: 10.1016/0925-4439(95)00091-7, PMID: 8608175

[331] Hope-Ross M Mahon GJ Johnston PB . Ocular siderosis. Eye (Lond). (1993) 7:419–25. doi: 10.1038/eye.1993.83, PMID: 8224298

[332] Guo D Du Y Liu X Li D Wei L Zhu X . Enhanced ferroptosis sensitivity promotes the formation of highly myopic cataract via the ddr2-hippo pathway. Cell Death Dis. (2025) 16:64. doi: 10.1038/s41419-025-07384-8, PMID: 39900894 PMC11790942

[333] Zhang Y Si W Mao Y Xu S Li F Liu J . Upregulation of ferroptosis in glucocorticoids-induced posterior subcapsular cataracts. Commun Biol. (2025) 8:613. doi: 10.1038/s42003-025-08067-y, PMID: 40234585 PMC12000516

[334] Fang S Lu Y Huang Y Zhou H Fan X . Mechanisms that underly T cell immunity in graves' Orbitopathy. Front Endocrinol (Lausanne). (2021) 12:648732. doi: 10.3389/fendo.2021.648732, PMID: 33868176 PMC8049604

[335] Mishra S Maurya VK Kumar S Ankita Kaur A Saxena SK . Clinical management and therapeutic strategies for the thyroid-associated ophthalmopathy: current and future perspectives. Curr Eye Res. (2020) 45:1325–41. doi: 10.1080/02713683.2020.1776331, PMID: 32567373

[336] Bartalena L Piantanida E Gallo D Lai A Tanda ML . Epidemiology, natural history, risk factors, and prevention of graves' Orbitopathy. Front Endocrinol (Lausanne). (2020) 11:615993. doi: 10.3389/fendo.2020.615993, PMID: 33329408 PMC7734282

[337] Tian X Li N Su R Dai C Zhang R . Selenium supplementation may decrease thyroid peroxidase antibody titer via reducing oxidative stress in euthyroid patients with autoimmune thyroiditis. Int J Endocrinol. (2020) 2020:9210572. doi: 10.1155/2020/9210572, PMID: 32676110 PMC7345605

[338] Negro R Hegedüs L Attanasio R Papini E . Winther KH. A 2018 european thyroid association survey on the use of selenium supplementation in graves' Hyperthyroidism and graves' Orbitopathy. Eur Thyroid J. (2019) 8:7–15. doi: 10.1159/000494837, PMID: 30800636 PMC6381891

[339] Douglas RS Kahaly GJ Patel A Sile S Thompson EHZ Perdok R . Teprotumumab for the treatment of active thyroid eye disease. N Engl J Med. (2020) 382:341–52. doi: 10.1056/NEJMoa1910434, PMID: 31971679

[340] Tsai CC Wu SB Cheng CY Kao SC Kau HC Lee SM . Increased response to oxidative stress challenge in graves' Ophthalmopathy orbital fibroblasts. Mol Vis. (2011) 17:2782–8. PMC3209425 22065933

[341] Ma C Li H Liu W Lu S Li X Chen J . Therapeutic effect of gypenosides on antioxidant stress injury in orbital fibroblasts of graves' Orbitopathy. J Immunol Res. (2022) 2022:4432584. doi: 10.1155/2022/4432584, PMID: 36157877 PMC9499793

[342] Gao Y Li W . Mechanisms of immune-related differentially expressed genes in thyroid-associated ophthalmopathy based on the geo database. Ann Transl Med. (2022) 10:926. doi: 10.21037/atm-22-3470, PMID: 36172114 PMC9511181

[343] Shin HR Cho WK Baek IC Lee NY Lee YJ Kim SK . Polymorphisms of irak1 gene on X chromosome is associated with hashimoto thyroiditis in korean children. Endocrinology. (2020) 161:bqaa088. doi: 10.1210/endocr/bqaa088, PMID: 32498091

[344] Li K Li H Xu W Liu W Du Y He JF . Research on the potential mechanism of gypenosides on treating thyroid-associated ophthalmopathy based on network pharmacology. Med Sci Monit. (2019) 25:4923–32. doi: 10.12659/msm.917299, PMID: 31268042 PMC6621796

[345] Luo P Liu D Zhang Q Yang F Wong YK Xia F . Celastrol induces ferroptosis in activated hscs to ameliorate hepatic fibrosis via targeting peroxiredoxins and ho-1. Acta Pharm Sin B. (2022) 12:2300–14. doi: 10.1016/j.apsb.2021.12.007, PMID: 35646542 PMC9136576

[346] Yu Y Jiang L Wang H Shen Z Cheng Q Zhang P . Hepatic transferrin plays a role in systemic iron homeostasis and liver ferroptosis. Blood. (2020) 136:726–39. doi: 10.1182/blood.2019002907, PMID: 32374849 PMC7414596

[347] Neag EJ Smith TJ . 2021 Update on thyroid-Associated ophthalmopathy. J Endocrinol Invest. (2022) 45:235–59. doi: 10.1007/s40618-021-01663-9, PMID: 34417736 PMC9455782

[348] Su Y Liu X Fang S Huang Y Li Y Zhong S . Age-related difference in extraocular muscles and its relation to clinical manifestations in an ethnically homogenous group of patients with graves' Orbitopathy. Graefes Arch Clin Exp Ophthalmol. (2022) 260:583–9. doi: 10.1007/s00417-021-05377-9, PMID: 34477926

[349] Huang Y Wu Y Zhang S Lu Y Wang Y Liu X . Immunophenotype of lacrimal glands in graves orbitopathy: implications for the pathogenesis of th1 and th17 immunity. Thyroid. (2022) 32:949–61. doi: 10.1089/thy.2021.0671, PMID: 35469435

[350] Liu X Cui Z Chen X Li Y Qiu J Huang Y . Ferroptosis in the lacrimal gland is involved in dry eye syndrome induced by corneal nerve severing. Invest Ophthalmol Vis Sci. (2023) 64:27. doi: 10.1167/iovs.64.7.27, PMID: 37326593 PMC10281063

[351] SanGiovanni JP Chew EY Clemons TE Davis MD Ferris FL 3rd Gensler GR . The relationship of dietary lipid intake and age-related macular degeneration in a case-control study: areds report no. 20. Arch Ophthalmol. (2007) 125:671–9. doi: 10.1001/archopht.125.5.671, PMID: 17502507

[352] Zhu M Yu J . Salidroside alleviates ferroptosis in fac-induced age-related macular degeneration models by activating nrf2/slc7a11/gpx4 axis. Int Immunopharmacol. (2024) 142:113041. doi: 10.1016/j.intimp.2024.113041, PMID: 39260309

[353] Huang K Deng H Wang S Zhang F Huang G Wang L . Melanin-like nanomedicine functions as a novel rpe ferroptosis inhibitor to ameliorate retinal degeneration and visual impairment in dry age-related macular degeneration. Adv Healthc Mater. (2024) 13:e2401613. doi: 10.1002/adhm.202401613, PMID: 39129350

[354] Ouyang J Zhou L Wang Q . Spotlight on iron and ferroptosis: research progress in diabetic retinopathy. Front Endocrinol (Lausanne). (2023) 14:1234824. doi: 10.3389/fendo.2023.1234824, PMID: 37772084 PMC10525335

[355] Singh J Sharma M Jain N Aftab I Vikram N Singh TP . Lactoferrin and its nano-formulations in rare eye diseases. Indian J Ophthalmol. (2022) 70:2328–34. doi: 10.4103/ijo.IJO_303_22, PMID: 35791114 PMC9426081

[356] Chan TC Wilkinson Berka JL Deliyanti D Hunter D Fung A Liew G . The role of reactive oxygen species in the pathogenesis and treatment of retinal diseases. Exp Eye Res. (2020) 201:108255. doi: 10.1016/j.exer.2020.108255, PMID: 32971094

[357] Moos WH Faller DV Glavas IP Harpp DN Kamperi N Kanara I . Treatment and prevention of pathological mitochondrial dysfunction in retinal degeneration and in photoreceptor injury. Biochem Pharmacol. (2022) 203:115168. doi: 10.1016/j.bcp.2022.115168, PMID: 35835206

[358] Zhuang S Ma Y Zeng Y Lu C Yang F Jiang N . Mettl14 promotes doxorubicin-induced cardiomyocyte ferroptosis by regulating the kcnq1ot1-mir-7-5p-tfrc axis. Cell Biol Toxicol. (2023) 39:1015–35. doi: 10.1007/s10565-021-09660-7, PMID: 34648132

[359] Millán I Desco MDC Torres-Cuevas I Pérez S Pulido I Mena-Mollá S . Pterostilbene prevents early diabetic retinopathy alterations in a rabbit experimental model. Nutrients. (2019) 12:82. doi: 10.3390/nu12010082, PMID: 31892189 PMC7019414

[360] Wang W Zhao H Chen B . Dj-1 Protects Retinal Pericytes against High Glucose-Induced Oxidative Stress through the Nrf2 Signaling Pathway. Sci Rep. (2020) 10:2477. doi: 10.1038/s41598-020-59408-2, PMID: 32051471 PMC7016111

[361] Wang J Zhang Q Chen E Zhao P Xu Y . Elabela promotes the retinal angiogenesis by inhibiting ferroptosis during the vaso-obliteration phase in mouse oxygen-induced retinopathy model. FASEB J. (2022) 36:e22257. doi: 10.1096/fj.202101785RRR, PMID: 35471770

[362] Mazzoli V Zhong LH Dang VT Shi Y Werstuck GH . Characterization of retinal microvascular complications and the effects of endoplasmic reticulum stress in mouse models of diabetic atherosclerosis. Invest Ophthalmol Vis Sci. (2020) 61:49. doi: 10.1167/iovs.61.10.49, PMID: 32852545 PMC7452854

[363] Wang C Wang K Li P . Blueberry anthocyanins extract attenuated diabetic retinopathy by inhibiting endoplasmic reticulum stress via the mir-182/ogg1 axis. J Pharmacol Sci. (2022) 150:31–40. doi: 10.1016/j.jphs.2022.06.004, PMID: 35926946

[364] Rani EA Janani R Chonche MJ Vallikannan B . Lactucaxanthin regulates the cascade of retinal oxidative stress, endoplasmic reticulum stress and inflammatory signaling in diabetic rats. Ocul Immunol Inflammation. (2023) 31:320–8. doi: 10.1080/09273948.2022.2027464, PMID: 35081014

[365] Tang X Li X Zhang D Han W . Astragaloside-iv alleviates high glucose-induced ferroptosis in retinal pigment epithelial cells by disrupting the expression of mir-138-5p/sirt1/nrf2. Bioengineered. (2022) 13:8240–54. doi: 10.1080/21655979.2022.2049471, PMID: 35302431 PMC9162003

[366] Zhang Y Zhou X Liang G Cui M Qiu Z Xu J . Iron-chelating and ros-scavenging polymers with thioketal and thioether bonds delivering ferroptosis inhibitor lip-1 provide a triple therapeutic strategy for retina ganglion cells in acute glaucoma. Adv Mater. (2025) 37:e2507526. doi: 10.1002/adma.202507526, PMID: 40641252

[367] Wang Z Wu S Zhu C Shen J . The role of ferroptosis in esophageal cancer. Cancer Cell Int. (2022) 22:266. doi: 10.1186/s12935-022-02685-w, PMID: 35999642 PMC9396912

[368] Boskovic O Medenica S Radojevic N Zarkovic M . Etanercept in the treatment of graves' Ophthalmopathy with primary hypothyroidism and rheumatoid arthritis. Cent Eur J Immunol. (2019) 44:463–5. doi: 10.5114/ceji.2019.92803, PMID: 32140060 PMC7050053

[369] Conrad M Proneth B . Selenium: tracing another essential element of ferroptotic cell death. Cell Chem Biol. (2020) 27:409–19. doi: 10.1016/j.chembiol.2020.03.012, PMID: 32275866

[370] D'Aprile S Denaro S Pavone AM Giallongo S Giallongo C Distefano A . Anaplastic thyroid cancer cells reduce cd71 levels to increase iron overload tolerance. J Transl Med. (2023) 21:780. doi: 10.1186/s12967-023-04664-9, PMID: 37924062 PMC10625232

[371] Ma R Gan L Guo J Peng Z Wu J Harrison AR . Insights into ferroptosis: targeting glycolysis to treat graves' Orbitopathy. J Clin Endocrinol Metab. (2022) 107:1994–2003. doi: 10.1210/clinem/dgac163, PMID: 35303084

[372] Yang Z Huang R Wang Y Guan Q Li D Wu Y . Sirt6 drives sensitivity to ferroptosis in anaplastic thyroid cancer through ncoa4-dependent autophagy. Am J Cancer Res. (2023) 13:464–74. doi: 10.62393/ajcr2023.464, PMID: 36895980 PMC9989618

[373] Chen S Diao J Yue Z Wei R . Identification and validation of ferroptosis-related genes and immune cell infiltration in thyroid associated ophthalmopathy. Front Genet. (2023) 14:1118391. doi: 10.3389/fgene.2023.1118391, PMID: 37021001 PMC10067720

[374] Liu L Lian N Shi L Hao Z Chen K . Ferroptosis: mechanism and connections with cutaneous diseases. Front Cell Dev Biol. (2022) 10:1079548. doi: 10.3389/fcell.2022.1079548, PMID: 36684424 PMC9846271

[375] Zhang F Lin B Huang S Wu P Zhou M Zhao J . Melatonin alleviates retinal ischemia-reperfusion injury by inhibiting P53-mediated ferroptosis. Antioxid (Basel). (2023) 12:1173. doi: 10.3390/antiox12061173, PMID: 37371903 PMC10295547

[376] Xiang W Li L Zhao Q Zeng Y Shi J Chen Z . PEDF protects retinal pigment epithelium from ferroptosis and ameliorates dry AMD-like pathology in a murine model. Geroscience. (2024) 46:2697–714. doi: 10.1007/s11357-023-01038-3, PMID: 38153666 PMC10828283

[377] Wu P Zhao L Du Y Lu J He Y Shu Q . Melatonin protects retinal pigment epithelium cells against ferroptosis in AMD via the PI3K/AKT/MDM2/P53 pathway. Front Pharmacol. (2025) 16:1543575. doi: 10.3389/fphar.2025.1543575, PMID: 40083383 PMC11903707

[378] Yang B Yang K Chen Y Li Q Chen J Li S . Exposure of A2E to blue light promotes ferroptosis in the retinal pigment epithelium. Cell Mol Biol Lett. (2025) 30:22. doi: 10.1186/s11658-025-00700-2, PMID: 39984833 PMC11846388

[379] Chen X Wang Y Wang JN Cao QC Sun RX Zhu HJ . m(6)A modification of circSPECC1 suppresses RPE oxidative damage and maintains retinal homeostasis. Cell Rep. (2022) 41:111671. doi: 10.1016/j.celrep.2022.111671, PMID: 36384115

[380] Zou R Zhang X Dai X Yuan Y Dai J Yuan F . The SDF-1α/MTDH axis inhibits ferroptosis and promotes the formation of anti-VEGF-resistant choroidal neovascularization by facilitating the nuclear translocation of SREBP1. Cell Biol Toxicol. (2025) 41:118. doi: 10.1007/s10565-025-10066-y, PMID: 40670757 PMC12267381

[381] Yang B Yang K Chen J Wu Y . Crocin protects the 661W murine photoreceptor cell line against the toxic effects of all-trans-retinal. Int J Mol Sci. (2024) 25:10124. doi: 10.3390/ijms251810124, PMID: 39337609 PMC11432120

[382] Shi W Dong Y Liu S Li F Zhu C . Corilagin alleviates ferroptosis in diabetic retinopathy by activating the Nrf2 signaling pathway. BioMed Pharmacother. (2024) 179:117409. doi: 10.1016/j.biopha.2024.117409, PMID: 39243434

[383] Liu Q Liu CQ Yi WZ Ouyang PW Yang BF Liu Q . Ferroptosis contributes to microvascular dysfunction in diabetic retinopathy. Am J Pathol. (2024) 194:1078–89. doi: 10.1016/j.ajpath.2024.01.019, PMID: 38417697

[384] Liu Z Gan S Fu L Xu Y Wang S Zhang G . 1,8-Cineole ameliorates diabetic retinopathy by inhibiting retinal pigment epithelium ferroptosis via PPAR-γ/TXNIP pathways. BioMed Pharmacother. (2023) 164:114978. doi: 10.1016/j.biopha.2023.114978, PMID: 37271074

[385] Xi X Chen Q Ma J Wang X Zhang J Li Y . Sestrin2 ameliorates diabetic retinopathy by regulating autophagy and ferroptosis. J Mol Histol. (2024) 55:169–84. doi: 10.1007/s10735-023-10180-3, PMID: 38165565 PMC10991044

[386] Zhang J Chang K Shangguan Y Luo R Bi Y Yu Z . Flotillin- 1 ameliorates experimental diabetic retinopathy by inhibiting ferroptosis in blood-retinal barrier. J Mol Med (Berl). (2025) 103:671–85. doi: 10.1007/s00109-025-02544-x, PMID: 40198383

[387] Shao J Bai Z Zhang L Zhang F . Ferrostatin-1 alleviates tissue and cell damage in diabetic retinopathy by improving the antioxidant capacity of the Xc(-)-GPX4 system. Cell Death Discov. (2022) 8:426. doi: 10.1038/s41420-022-01141-y, PMID: 36284090 PMC9596714

[388] Liao Q Li Y Cui M Liu M . m6A demethylase ALKBH5 reduces ferroptosis in diabetic retinopathy through the m6A-YTHDF1-ACSL4 axis. Diabetes Med. (2025) 42:e70033. doi: 10.1111/dme.70033, PMID: 40210448

[389] Cai Y Peng S Duan B Shao Y Li X Zou H . Isoquercetin alleviates diabetic retinopathy via inhibiting p53-mediated ferroptosis. Cell Biol Int. (2025) 49:852–64. doi: 10.1002/cbin.70027, PMID: 40329699

[390] Wang Y Song SY Song Y Wang Y Wan ZW Sun P . Resveratrol protects müller cells against ferroptosis in the early stage of diabetic retinopathy by regulating the nrf2/GPx4/PTGS2 pathway. Mol Neurobiol. (2025) 62:3412–27. doi: 10.1007/s12035-024-04496-8, PMID: 39292340

[391] Yuan F Han S Li Y Li S Li D Tian Q . miR-214-3p attenuates ferroptosis-induced cellular damage in a mouse model of diabetic retinopathy through the p53/SLC7A11/GPX4 axis. Exp Eye Res. (2025) 253:110299. doi: 10.1016/j.exer.2025.110299, PMID: 39978746

[392] Li S Lu S Wang L Liu S Zhang L Du J . Effects of amygdalin on ferroptosis and oxidative stress in diabetic retinopathy progression via the NRF2/ARE signaling pathway. Exp Eye Res. (2023) 234:109569. doi: 10.1016/j.exer.2023.109569, PMID: 37422064

[393] Peng Y Hu L Xu H Fang J Zhong H . Resveratrol alleviates reactive oxygen species and inflammation in diabetic retinopathy via SIRT1/HMGB1 pathway-mediated ferroptosis. Toxicol Appl Pharmacol. (2025) 495:117214. doi: 10.1016/j.taap.2024.117214, PMID: 39719253

[394] Zhou J Mei X Zhan L Liu X Wang B Xu B . Structural characterization and protective effects of a glucuronogalactomannan from Tetrastigma hemsleyanum against diabetic retinopathy via targeting ferroptosis pathway. Carbohydr Polym. (2025) 366:124059. doi: 10.1016/j.carbpol.2025.124059, PMID: 40733853

[395] Ren M Xu Q Luan J Ni Y Xie B . Mir-509-3p targets SLC25A13 to regulate ferroptosis and protect retinal endothelial cells in diabetic retinopathy. Acta Diabetol. (2025) 62:831–44. doi: 10.1007/s00592-024-02400-3, PMID: 39508857

[396] Zhong L Meng X Huang J Hao W Zuo Y . Expression of YAP suppresses cell proliferation and elevates the sensitivity of chemotherapy in retinoblastoma cells through lipid-peroxidation induced ferroptosis. Chin Clin Oncol. (2023) 12:52. doi: 10.21037/cco-23-97, PMID: 37964544

[397] Chi M Zhao Y Yuan B Qiu Z Peng R Hong J . MiR-23a-3p targets PTEN as a novel anti-ferroptosis regulator in Fuchs endothelial corneal dystrophy. Exp Eye Res. (2025) 250:110180. doi: 10.1016/j.exer.2024.110180, PMID: 39581360

[398] Wang H Yang Y Yu H Ma L Qi X Qu J . Self-cascade API nanozyme for synergistic anti-inflammatory, antioxidant, and ferroptosis modulation in the treatment of corneal neovascularization. Small. (2025) 21:e2407751. doi: 10.1002/smll.202407751, PMID: 39648573

[399] Chu G Wang J Wang Z Cai X Shi S Qing Q . Autologous serum from sjögren's syndrome patients mitigates ferroptosis in hyperosmolarity-induced corneal epitheliopathy. J Ocul Pharmacol Ther. (2025) 41:397–407. doi: 10.1089/jop.2025.0025, PMID: 40495717

[400] Zhao L Dong X Guo B Song J Bi H . Quercetin-loaded exosomes delivery system prevents myopia progression by targeting endoplasmic reticulum stress and ferroptosis in scleral fibroblasts. Mater Today Bio. (2025) 32:101896. doi: 10.1016/j.mtbio.2025.101896, PMID: 40520556 PMC12167070

[401] Hou C Xiao J Wang Y Pan X Liu K Lu K . Astaxanthin activated the slc7a11/gpx4 pathway to inhibit ferroptosis and enhance autophagy, ameliorating dry eye disease. Front Pharmacol. (2024) 15:1407659. doi: 10.3389/fphar.2024.1407659, PMID: 39224780 PMC11366873

[402] Wang J Liu Y Zong B Zhao S Li Y Zhang Z . Qingxuan Runmu Yin alleviates dry eye disease via inhibition of the HMOX1/HIF-1 pathway affecting ferroptosis. Front Pharmacol. (2024) 15:1391946. doi: 10.3389/fphar.2024.1391946, PMID: 39329129 PMC11425584

[403] Feng L Wang C Zhang C Zhang W Zhu W He Y . p38 MAPK inhibitor SB202190 suppresses ferroptosis in the glutamate-induced retinal excitotoxicity glaucoma model. Neural Regener Res. (2024) 19:2299–309. doi: 10.4103/1673-5374.391193, PMID: 38488564 PMC11034608

[404] Wang J Liu Y Rong R Xia X . Bilirubin-polymer nanocarriers enable targeted farrerol delivery for glaucoma neuroprotection via Nrf2-mediated ferroptosis/apoptosis inhibition. Mater Today Bio. (2025) 35:102304. doi: 10.1016/j.mtbio.2025.102304, PMID: 41050099 PMC12489836

[405] Feng Y Wang X Li P Shi X Prokosch V Liu H . Exogenous hydrogen sulfide and NOX2 inhibition mitigate ferroptosis in pressure-induced retinal ganglion cell damage. Biochim Biophys Acta Mol Basis Dis. (2025) 1871:167705. doi: 10.1016/j.bbadis.2025.167705, PMID: 39914725

[406] Chen X Rong Y Jiang Y Zhang Q Xiang S Chen Z . Vitamin K1 alleviates retinal inflammation following acute ocular hypertension by modulating microglial ferroptosis. Invest Ophthalmol Vis Sci. (2025) 66:46. doi: 10.1167/iovs.66.4.46, PMID: 40244608 PMC12013678

[407] Zuo X Wang X Xie J Jia Y . Emodin alleviates the damage to lens epithelial cells in diabetic cataract by repressing the p53-mediated ferroptosis pathway. Int Ophthalmol. (2025) 45:141. doi: 10.1007/s10792-025-03513-6, PMID: 40175804

[408] Cui T Liu Y Gao F Wang J Lu L Zhang J . Asparagine alleviates naphthalene-induced lens opacity by suppressing ferroptosis. Exp Eye Res. (2025) 255:110362. doi: 10.1016/j.exer.2025.110362, PMID: 40147683

[409] Kong D Liu Y Li L Wang H Li K Zheng G . Astaxanthin ameliorates oxidative stress in lens epithelial cells by regulating GPX4 and ferroptosis. Chem Biol Interact. (2023) 383:110684. doi: 10.1016/j.cbi.2023.110684, PMID: 37648051

[410] Chhunchha B Kubo E Krueger RR Singh DP . Hydralazine revives cellular and ocular lens health-span by ameliorating the aging and oxidative-dependent loss of the nrf2-activated cellular stress response. Antioxid (Basel). (2023) 12:140. doi: 10.3390/antiox12010140, PMID: 36671002 PMC9854670

[411] Zheng Y Liu Y Ge J Wang X Liu L Bu Z . Resveratrol protects human lens epithelial cells against H2o2-induced oxidative stress by increasing catalase, sod-1, and ho-1 expression. Mol Vis. (2010) 16:1467–74., PMID: 20806083 PMC2925910

[412] Lledó VE Alkozi HA Sánchez-Naves J Fernandez-Torres MA Guzman-Aranguez A . Melatonin counteracts oxidative damage in lens by regulation of nrf2 and nlrp3 inflammasome activity. Exp Eye Res. (2022) 215:108912. doi: 10.1016/j.exer.2021.108912, PMID: 34965405

[413] Babizhayev MA Burke L Micans P Richer SP . N-acetylcarnosine sustained drug delivery eye drops to control the signs of ageless vision: glare sensitivity, cataract amelioration and quality of vision currently available treatment for the challenging 50,000-patient population. Clin Interv Aging. (2009) 4:31–50., PMID: 19503764 PMC2685223

[414] Yang M So KF Lam WC Lo ACY . Novel programmed cell death as therapeutic targets in age-related macular degeneration? Int J Mol Sci. (2020) 21:7279. doi: 10.3390/ijms21197279, PMID: 33019767 PMC7582463

[415] Dou J Liu X Yang L Huang D Tan X . Ferroptosis interaction with inflammatory microenvironments: mechanism, biology, and treatment. BioMed Pharmacother. (2022) 155:113711. doi: 10.1016/j.biopha.2022.113711, PMID: 36126457

[416] Tan Y Huang J Li D Zou C Liu D Qin B . Single-cell rna sequencing in dissecting microenvironment of age-related macular degeneration: challenges and perspectives. Ageing Res Rev. (2023) 90:102030. doi: 10.1016/j.arr.2023.102030, PMID: 37549871

[417] Campochiaro PA Marcus DM Awh CC Regillo C Adamis AP Bantseev V . The port delivery system with ranibizumab for neovascular age-related macular degeneration: results from the randomized phase 2 ladder clinical trial. Ophthalmology. (2019) 126:1141–54. doi: 10.1016/j.ophtha.2019.03.036, PMID: 30946888

[418] Chen X Comish PB Tang D Kang R . Characteristics and biomarkers of ferroptosis. Front Cell Dev Biol. (2021) 9:637162. doi: 10.3389/fcell.2021.637162, PMID: 33553189 PMC7859349

[419] Agmon E Solon J Bassereau P Stockwell BR . Modeling the effects of lipid peroxidation during ferroptosis on membrane properties. Sci Rep. (2018) 8:5155. doi: 10.1038/s41598-018-23408-0, PMID: 29581451 PMC5979948

[420] Liu X Gong T . Artificial intelligence and evidence-based research will promote the development of traditional medicine. Acupuncture Herbal Med. (2024) 4:134–5. doi: 10.1097/HM9.0000000000000100

[421] Zhou H Li J Sun F Wang F Li M Dong Y . A review on recent advances in aloperine research: pharmacological activities and underlying biological mechanisms. Front Pharmacol. (2020) 11:538137. doi: 10.3389/fphar.2020.538137, PMID: 33536900 PMC7849205

[422] Liu M Li W Chen Y Wan X Wang J . Fucoxanthin: A promising compound for human inflammation-related diseases. Life Sci. (2020) 255:117850. doi: 10.1016/j.lfs.2020.117850, PMID: 32470447

[423] Hao M Liu Y Chen P Jiang H Kuang HY . Astragaloside iv protects rgc-5 cells against oxidative stress. Neural Regener Res. (2018) 13:1081–6. doi: 10.4103/1673-5374.233452, PMID: 29926836 PMC6022471

[424] Meng F Guo B Ma YQ Li KW Niu FJ . Puerarin: A review of its mechanisms of action and clinical studies in ophthalmology. Phytomedicine. (2022) 107:154465. doi: 10.1016/j.phymed.2022.154465, PMID: 36166943

[425] Boateng ID . Potentialities of ginkgo extract on toxicants, toxins, and radiation: A critical review. Food Funct. (2022) 13:7960–83. doi: 10.1039/d2fo01298g, PMID: 35801619

[426] Li Y Wang K Zhu X Cheng Z Zhu L Murray M . Ginkgo biloba extracts protect human retinal müller glial cells from T-bhp induced oxidative damage by activating the ampk-nrf2-nqo-1 axis. J Pharm Pharmacol. (2023) 75:385–96. doi: 10.1093/jpp/rgac095, PMID: 36583518

[427] Zhu Z Wang Y Deng Z Lei P Liu Q Guo J . The hemerocallis citrinaExtracts ameliorate radiation-induced ferroptosis in lo2 cells through the nrf2-xct/gpx4 pathway. Acupuncture Herbal Med. (2024) 4:513–24. doi: 10.1097/HM9.0000000000000120

[428] Husain A Chanana H Khan SA Dhanalekshmi UM Ali M Alghamdi AA . Chemistry and pharmacological actions of delphinidin, a dietary purple pigment in anthocyanidin and anthocyanin forms. Front Nutr. (2022) 9:746881. doi: 10.3389/fnut.2022.746881, PMID: 35369062 PMC8969030

[429] Ngamsamer C Sirivarasai J Sutjarit N . The benefits of anthocyanins against obesity-induced inflammation. Biomolecules. (2022) 12:852. doi: 10.3390/biom12060852, PMID: 35740977 PMC9230453

[430] Dörschmann P Klettner A . Fucoidans as potential therapeutics for age-related macular degeneration-current evidence from *in vitro* research. Int J Mol Sci. (2020) 21:9272. doi: 10.3390/ijms21239272, PMID: 33291752 PMC7729934

